# Community Profiling of *Fusarium* in Combination with Other Plant-Associated Fungi in Different Crop Species Using SMRT Sequencing

**DOI:** 10.3389/fpls.2017.02019

**Published:** 2017-11-28

**Authors:** Florian Walder, Klaus Schlaeppi, Raphaël Wittwer, Alain Y. Held, Susanne Vogelgsang, Marcel G. A. van der Heijden

**Affiliations:** ^1^Plant-Soil Interactions, Research Division Agroecology and Environment, Agroscope, Zurich, Switzerland; ^2^Ecology of Noxious and Beneficial Organisms, Research Division Plant Protection, Agroscope, Zurich, Switzerland; ^3^Department of Evolutionary Biology and Environmental Studies, University of Zürich, Zurich, Switzerland; ^4^Plant-Microbe Interactions, Institute of Environmental Biology, Faculty of Science, Utrecht University, Utrecht, Netherlands

**Keywords:** *Fusarium* community analysis, plant-associated fungi, single molecule real-time (SMRT) sequencing, PacBio, Mycobiome

## Abstract

Fusarium head blight, caused by fungi from the genus *Fusarium*, is one of the most harmful cereal diseases, resulting not only in severe yield losses but also in mycotoxin contaminated and health-threatening grains. Fusarium head blight is caused by a diverse set of species that have different host ranges, mycotoxin profiles and responses to agricultural practices. Thus, understanding the composition of *Fusarium* communities in the field is crucial for estimating their impact and also for the development of effective control measures. Up to now, most molecular tools that monitor *Fusarium* communities on plants are limited to certain species and do not distinguish other plant associated fungi. To close these gaps, we developed a sequencing-based community profiling methodology for crop-associated fungi with a focus on the genus *Fusarium*. By analyzing a 1600 bp long amplicon spanning the highly variable segments ITS and D1–D3 of the ribosomal operon by PacBio SMRT sequencing, we were able to robustly quantify *Fusarium* down to species level through clustering against reference sequences. The newly developed methodology was successfully validated in mock communities and provided similar results as the culture-based assessment of *Fusarium* communities by seed health tests in grain samples from different crop species. Finally, we exemplified the newly developed methodology in a field experiment with a wheat-maize crop sequence under different cover crop and tillage regimes. We analyzed wheat straw residues, cover crop shoots and maize grains and we could reveal that the cover crop hairy vetch (*Vicia villosa*) acts as a potent alternative host for *Fusarium* (OTU *F.ave/tri*) showing an eightfold higher relative abundance compared with other cover crop treatments. Moreover, as the newly developed methodology also allows to trace other crop-associated fungi, we found that vetch and green fallow hosted further fungal plant pathogens including *Zymoseptoria tritici*. Thus, besides their beneficial traits, cover crops can also entail phytopathological risks by acting as alternative hosts for *Fusarium* and other noxious plant pathogens. The newly developed sequencing based methodology is a powerful diagnostic tool to trace *Fusarium* in combination with other fungi associated to different crop species.

## Introduction

*Fusarium* represents a complex fungal genus inhabiting a vast range of different ecological niches and is often found in associations with plants (Summerell et al., 2010). A set of plant-associated *Fusarium* species include some of the most important plant pathogens worldwide causing the disease complexes Fusarium ear rot (FER) in maize (*Zea mays*) and Fusarium head blight (FHB) in several small-grain cereals, such as wheat (*Triticum aestivum*) and barley (*Hordeum vulgare*) (Parry et al., 1995; Desjardins, 2003; Schöneberg et al., 2016). The danger of both FER and FHB epidemics is not only due to the severe yield losses they cause but also because of the contamination of grains with harmful mycotoxins, which might devastate entire harvests (Bottalico and Perrone, 2002; Nganje et al., 2004; Reddy et al., 2010; Vogelgsang et al., 2011). Hence, *Fusarium* constitutes not only a severe threat to plant health but also to food and feed safety and sustainable agricultural production.

For several reasons, it is of great agronomical importance to understand the structure of *Fusarium* communities. First, the FER and FHB disease complexes are caused by 15–20 different species, where usually more than one species are simultaneously found in individual grain samples (Xu et al., 2005; Xu and Nicholson, 2009). Important causal species of FER in maize are *Fusarium graminearum, F. verticillioides, F. proliferatum*, and *F. subglutinans* (Desjardins, 2003; Dorn et al., 2009; Summerell et al., 2010), while the main causal species for FHB include *F. graminearum*, *F. culmorum*, *F. poae* and *F. avenaceum* (Parry et al., 1995; Xu et al., 2005). Additionally, it is important to note that different *Fusarium* species differ significantly in their response to diverse environmetal conditions and to particular agricultural practices (Edwards, 2004; Xu and Nicholson, 2009). Finally, disease severity and also the type and amount of mycotoxin production depends on the identity of *Fusarium* involved, as species vary substantially in their aggressiveness, host range and mycotoxin proflies (Bottalico and Perrone, 2002; Uhlig et al., 2006; Stȩpień et al., 2011). Thus, revealing the composition of the *Fusarium* community is important to understand its agro-ecological impact and finally to develop effective control measures (Xu and Nicholson, 2009).

The genetic diversity within the *Fusarium* genus is complex, exhibiting high genetic variablity within morphologically defined species (Leslie et al., 2007; O’Donnell et al., 2009). A comprehensive molecular study revealed that most of the species within this genus could be resolved into monophyletic species complexes that consist of multiple morphological cryptic species (O’Donnell et al., 2013). Hence, the phylogenetic relationship among *Fusarium* species remains unclear and the construction of a reliable taxonomic system based on the combination of morphological, molecular, toxicological, and biological traits is needed (Leslie and Bowden, 2008; Watanabe, 2013). In the present study, we are using a species definition which is based on the morphological species concept. Thus, *Fusarium* species are defined in a broader sense and we do not differentiate between cryptic species within given species complexes. We therefore refer in the following to species as *sensu lato* (*s.l.*).

Suitable tools to identify and quantify *Fusarium* communities are limited. The assessment of *Fusarium* is traditionally performed employing so-called seed health tests (SHTs; Dorn et al., 2011; Vogelgsang et al., 2011; Infantino et al., 2012), which are cultivation-based assays on agar media making use of species-specific morphological characteristics. However, this approach is laborious and only applicable if highly experienced and specialized personnel is available (Summerell and Leslie, 2011). On the other hand, molecular detection of *Fusarium* is often based on species-specific real-time quantitative polymerase chain reactions (qPCR; Gao et al., 2004; Waalwijk et al., 2004; Hogg et al., 2007; Edwards et al., 2012). qPCR assays allow a precise quantification of *Fusarium*, but are restricted to a limited number of species. The state-of-the-art technique for molecular identification of fungal communities is high-throughput amplicon sequencing providing relative abundances of operational taxonomical units (OTUs; Lindahl et al., 2013). A major drawback of high throughput sequencing approaches is that they often fail to resolve microbes on species levels (Eren et al., 2013). So far, only one high-throughput sequencing approach is available able to resolve *Fusarium* fungi down to morphological species level (Karlsson et al., 2016). However, this approach is restricted to the genus *Fusarium* because it uses taxon-specific primers. The use of universal fungal primers has many benefits over taxa specific approaches because other fungi (including a wide range of fungal pathogens) can be detected at the same time providing important additional information. Also, commensal plant-associated fungal communities can interact with pathogens, thus determining their infection success (Berendsen et al., 2012; Agler et al., 2016). Hence, sequencing tools that simultaneously capture pathogens (e.g., *Fusarium*) and non-pathogenic fungi can provide additional information and might thus contribute to the search for beneficial plant-associated fungi with potential bio-control activity.

In the present work, we are aiming to develop a sequencing based community profiling methodology enabling the detection of *Fusarium* down to morphological species level in the frame of a universal fungal approach. Most promising loci for a sequence-based identification of *Fusarium* taxa are the translation elongation factor 1α, β-tubulin, and the largest subunit of RNA polymerase (Balajee et al., 2009; O’Donnell et al., 2015). However, these loci are less practical for a universal approach as available primers for such markers usually amplify a narrow taxonomic range (Schoch et al., 2012). More suitable for a universal fungal target locus appears to be the nuclear ribosomal operon, particularly its internal transcribed spacer (ITS; Schoch et al., 2012; Lindahl et al., 2013). Although the ITS segment is highly variable, it lacks species-level resolution in some fungal groups including *Fusarium* (O’Donnell et al., 1998; Yli-Mattila et al., 2004; Santamaria et al., 2009). In addition, paralogous sequences for the ITS segment were found within *Fusarium* species, with the consequence that the ITS segment is not always reflecting the *Fusarium* phylogeny (Waalwijk et al., 1996; O’Donnell and Cigelnik, 1997). The segment at the 3′-prime end of the large subunit (LSU) of the ribosomal operon including the highly variable segments D1 and D2 (D1–D2) (Nilsson et al., 2013) provide a valid alternative. The D1–D2 segment was also used to distinguish among *Fusarium* species (Guadet et al., 1989; Hennequin et al., 1999). However, it has been shown that the D1–D2 segment on its own was not able to resolve *Fusarium* taxa down to species or isolate level (O’Donnell et al., 2008b). The combination of the highly variable ITS and D1–D2 segments has been tested as well, but showed an inferior performance than other loci (O’Donnell et al., 2008a, 2009).

Nonetheless, as the ribosomal operon offers some indisputable advantages for the sequencing based assessment of fungi over the whole kingdom, we aimed to explore this possibility based on the analysis of a large amplicon spanning the entire ITS and the D1–D2 segment of the ribosomal operon using single molecule real time (SMRT) sequencing methodology. SMRT sequencing presents a valid technology for high-resolution community sequencing of bacteria and fungi (Franzén et al., 2015; Schlaeppi et al., 2016; Schloss et al., 2016; Singer et al., 2016; Tedersoo et al., 2017). We hypothesized that such an approach will permit the description of *Fusarium* communities down to morphological species level.

The goal of this study was the development of a sequencing-based methodology that robustly quantifies known *Fusarium* in the phyllosphere, including shoots and grains, of different plant species. To achieve this aim, we first examined the ribosomal operon for a suitable marker region to discriminate *Fusarium* identities based on a set of morphologically validated species and isolates. We then evaluated four PCR primer combinations to capture the taxonomic breath of *Fusarium* taxa and how accurately they quantify them. Subsequently, we successfully validated the diagnostic power of the new methodology in several samples of different crops in comparison to sequencing-independent and cultivation-based SHTs. Finally, we applied the new sequencing-based methodology to a test case under field conditions. We monitored *Fusarium* species in a field experiment investigating the effect of different cover crops and tillage regimes to get insights on the dispersal of FHB causing species along a wheat-maize crop sequence. Both tillage and cover crops can have a substantial impact on plant yield (Wittwer et al., 2017) and on the dispersal of *Fusarium* species (Dill-Macky and Jones, 2000; Edwards, 2004; Friberg et al., 2009). Thus, the potential agronomic benefits of cover crops and reduced tillage systems can only become effective if their phytopathological risks for crop sequences are thoroughly assessed.

## Materials and Methods

### Analysis of Ribosomal Operon Sequences of Swiss *Fusarium* Isolates

Forty-four isolates belonging to 14 different *Fusarium* species based on morphological characterization reflecting the diversity of FHB causing species in Switzerland were selected to survey the genetic variability of the ribosomal operon (ITS, LSU) among the genus *Fusarium* (Supplementary Table S1). The single conidia isolates were collected in the frame of a Swiss *Fusarium* monitoring effort (Dorn et al., 2009; Vogelgsang et al., 2009; Schöneberg et al., 2016). Fungal mycelium was used for DNA extraction (see Supplementary Methods for details). Ribosomal operon sequences of the 44 *Fusarium* isolates were obtained by amplification with the PCR primer pair *ITS1F* and *LR6* (Supplementary Table S2 for references), spanning the entire ITS region and the highly variable segments D1, D2 and D3 of the LSU, and subsequent Sanger sequencing (Microsynth AG, Balgach, Switzerland; see Supplementary Methods for details).

**FIGURE 1:**
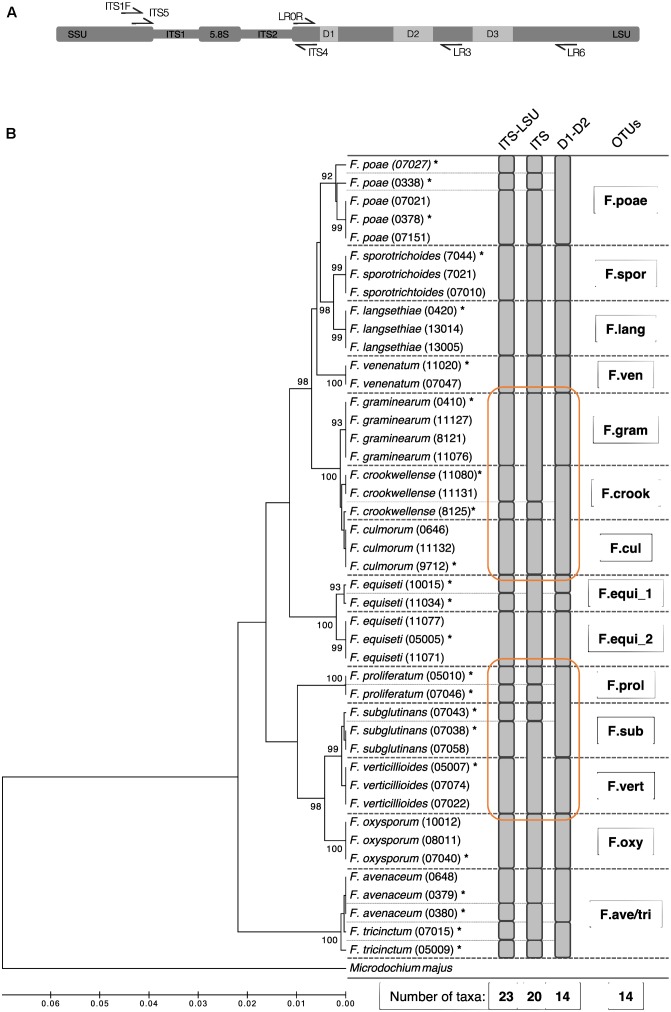
Phylogenetic diversity and taxonomic clustering of 44 Swiss *Fusarium* isolates. **(A)** Primer binding at the ribosomal operon used to defined different segments: *ITS1F* and *LR6* (used in this study) for the ITS-LSU, *ITS5* and *ITS4* for the ITS, and *LR0R* and *LR3* for the D1–D2 segment. **(B)** The tree was constructed using the UPGMA method based on the partial sequences of the ribosomal operon amplified by the primer pair ITS1F and LR6 (∼1.6 kb). The percentage of replicate trees in which the associated taxa clustered together in the bootstrap test (500 replicates) is shown next to the branches. The distances were computed using the Jukes-Cantor method in MEGA7 (Tamura et al., 2013). *Microdochium majus* was used as outgroup. Fine dashed lines indicate sequence differences among isolates. Gray bars indicate clusters computed by USEARCH with a similarity threshold of 100% using different segments of the ribosomal operon (ITS-LSU, ITS, LSU). Stars above isolate names mark sequences which were considered for the *Fusarium* sequence reference dataset. Bold dashed lines indicate isolates binned into the same *Fusarium* OTU by the method introduced in the present study. Orange circles highlight superior clustering of the ITS-LSU segment compared to ITS and LSU on its own.

Based on these Sanger sequences, we evaluated the entire amplicon of *ITS1F* and *LR6* region and additionally the ITS and the D1–D2 segment on its own for their suitability to discriminate the *Fusarium* isolates at the morphological species level (**Figure 1A**). The different segments of the ribosomal operon were extracted *in silico* with flexbar (Dodt et al., 2012) using for the ITS segment the PCR primers *ITS5* and *ITS4*, and for the D1–D2 segment of the LSU region the primers *LR0R* and *LR3* (see Supplementary Table S2 for details and references, **Figure 1A**). We clustered the sequences for each operon segment and defined OTUs at similarity levels of 100% using USEARCH (see below). For each segment, we report the taxonomic resolution among the 44 isolate sequences (**Figure 1B**). Utilizing the full-length operon, we found 23 unique sequences among the 44 tested isolates. The sequences determined in this study were deposited in GenBank (Accession Numbers: MG274294-MG274317).

### Performance of Different Primer Pairs

Initially, different primer pairs were tested for their *Fusarium* community profiling performance on three different test samples. The three test samples included maize grains infected by *Fusarium graminearum*, shoot material of winter wheat and a composite sample of co-occurring weeds sampled on the OSCAR field experiment (see below for details). Maize grains were dried with forced air at 32°C for 3 days and ground to a fine powder with a sample mill (Cyclotec, 1093, IG AG, Zurich, Switzerland). Leaf samples were washed after harvest with a PBS-S buffer (130 mM NaCl, 7 mM Na_2_HPO_4_, 3 mM NaH_2_PO_4_, pH 7.0, 0.02% Tween 20), two times for 20 min on a platform shaker at 120 rpm to remove microbes that are weakly associated. Samples were dried with laboratory grade tissue papers, lyophilized for 48 h and ground in a ball mill (Retsch MM200 mixer mill, Retsch GmbH, Hann, Germany) for 1.5 min with 30 Hz. DNA was extracted from the three different samples using Nucleo spin lysis buffer PL1 for 15 min at 65°C followed by the Nucleo spin plant II extraction kit protocol (Macherey & Nagel, Düren, Germany), and was used as template for PCR with the following primer combinations; *ITS1f:LR5, ITS1f:LR6, fITS7:LR5 and fITS7:LR6* (Supplementary Table S2). Amplicons were analyzed with PacBio SMRT sequencing (details are described below).

### DNA Extraction, PCR Cycling and SMRT Sequencing for Community Profiling

Fungal or plant material (∼20 mg dry weight) was used for DNA extraction (Nucleo spin plant II extraction kit, Macherey & Nagel, Düren, Germany). The DNA samples were amplified by different primers targeting the ITS region and the 5′-end of the LSU as indicated above. The forward and reverse primers were synthesized with a 5-nucleotide-long padding sequence followed by barcode tags at the 5′-end to allow multiplexing of samples within a single sequencing run. Details of PCR and purification are described in Supplementary Methods. Library preparation and SMRT sequencing were conducted at the Functional Genomics Centre Zurich^1^ on the PacBio^®^ RS II Instrument (PacBio, San Diego, CA, United States).

### Sequence Data Processing

The sequence data processing was mainly conducted following the pipeline established by Schlaeppi et al. (2016) and is described in detail in the Supplementary Methods. In brief, the SMRT Portal was used to extract from the raw data (available from the European Nucleotide Archive, study accession number: PRJEB23144) the circular consensus sequences (CCS) of at least five passes yielding in similar error rates as 454 or MiSeq sequencing platforms (Schlaeppi et al., 2016). Subsequently, the CCS reads were quality filtered in mothur (v.1.35.0; Schloss et al., 2009). Quality reads were demultiplexed based on the barcode-primer sequences using fexbar (Dodt et al., 2012).

QIIME 1.8 (Caporaso et al., 2010) was used for further downstream read processing. *De novo* chimera detection was performed on quality reads using UCHIME (Edgar et al., 2011). Reads were clustered into OTUs according to the open reference protocol using UCLUST (Edgar, 2010) within QIIME applying a sequence similarity threshold of 98%. This involves first clustering reads against the hand-curated *Fusarium* reference sequence dataset composed of the 23 unique *Fusarium* full-length amplicon sequences (see above). The reads clustered to the *Fusarium* reference dataset were subsequently binned to *Fusarium* OTUs with a custom R code (**Figure 1B**; see Supplementary Data). Reads that failed to assign to the reference dataset were clustered *de novo* into OTUs with UCLUST. OTUs of low abundance (less than 0.1% global abundance and less than 0.5% abundance within a specific sample) were removed from the data set yielding in a data set of abundant community members only. *Fusarium* and *de novo* OTUs were classified taxonomically against the UNITE database (Kõljalg et al., 2013) and against the fungal LSU training set of the Ribosomal Database Project (RDP) (Cole et al., 2014). The OTU and taxonomy tables were filtered to exclude OTUs classified as non-fungal. Results of mock communities were used to define optimal settings for quality filtering, demulitplexing and cut-off for low abundant OTUs (Bokulich et al., 2012).

### Evaluation of *Fusarium* Community Profile Performance

The sequence based *Fusarium* community profiling method was evaluated by three different approaches; (i) by determining artificial *Fusarium* mock communities, and by comparing sequence-based profiling to the assessment of *Fusarium* communities with the cultivation-based seed health test (SHT) in different field samples of (ii) Swiss *Fusarium* monitoring and (iii) of a *Fusarium*-inoculation field experiment.

Mock communities were composed of ten *Fusarium* species *s.l.* and isolates: *F. avenaceum* (0379), *F. crookwellense* (0825), *F. culmorum* (11132), *F. equiseti* (00505), *F. equiseti* (10015), *F. graminearum* (0410), *F. langsethiae* (0420), *F. poae* (0338), *F. sporotrichioides* (07010) and *F. verticillioides* (05007; Supplementary Table S1). *Fusarium* isolates were chosen to represent the expected phylogenetic diversity of *Fusarium* fungi associated to small-grain cereals and maize in Swiss agro-ecosystems (Dorn et al., 2009; Vogelgsang et al., 2011). Additionally, closely related isolates [e.g., *F. equiseti* (05005) and *F. equiseti* (10015), or *F. crookwellense* (0825) and *F. culmorum* (11132), **Figure 1B**] were included to test for resolution power. Two different types of mock communities were assembled; (i) all species were added at equal concentrations resulting in the equal mock community, or (ii) species were added in three difference concentration ratios, i.e., *F. culmorum* (11132) and *F. langsethiae* (0420) were ten times higher, and *F. equiseti* (10015) and *F. graminearum* (0410) were ten times lower concentrated as the remaining six isolates in the staggered mock community.

In SHTs, *Fusarium* species *s.l.* from grain samples are determined based on species-specific morphological characteristics (see details in Supplementary Methods). Briefly, 100 surface-sterilized grains were plated on potato dextrose agar, after 7 days of incubation at 19 ± 1°C with a photoperiod of 12 h dark/12 h near-UV light, *Fusarium* species colonizing the grains were identified using morphological characteristics according to Nelson et al. (1983) and Leslie and Summerell (2008). Results are presented as incidence of each *Fusarium* species expressed in percentage.

To evaluate the community profile methodology in an experimental environment, we re-assessed *Fusarium* communities previously profiled with SHT in a winter wheat field experiment where plots were or were not artificially inoculated with *F. poae* conidia. Treatments consisted of inoculations with a water-based conidial suspension of *F. poae* with Tween 20^®^ or with Tween 20^®^ alone, serving as the control treatment. Each treatment was replicated four times. Plots were combine-harvested and the grains were passed through a grain cleaning machine. To obtain a representative subsample, grains for the STHs were passed through a mechanical grain divider (detailed information regarding the *Fusarium* inoculation field experiment is available in the Supplementary Methods).

Finally, we used 15 additional samples of winter wheat, maize and barley to compare the *Fusarium* communities assessed by SHT to the community profile based on sequencing (Supplementary Table S3). Winter wheat and barley samples originate from commercial samples of Swiss farmers taken in the frame of the Swiss *Fusarium* monitoring. Maize samples originate from a maize variety trial at Agroscope. Grains of all three crops were passed through a grain-cleaning machine and randomized using a grain divider.

### *Fusarium* Diversity and Distribution along a Wheat-Maize Crop Sequence

The investigations on diversity and distribution of *Fusarium* along the crop sequence were within an OSCAR field trial during the growing years 2014 and 2015 (details on the OSCAR field trial is available in the Supplementary Methods). The field experiment exhibiting a wheat-maize crop sequence was arranged in a strip-split-plot design with four replicates. On the main plots, two different tillage intensities were applied: (i) conventional inversion tillage by mouldboard plowing (ct) and (ii) no-tillage (nt), both employed before maize sowing in the second year of each experiment. In addition, each main plot was divided into four sub plots where the cover crop treatments were applied: (i) subterranean clover (*Trifolium subterraneum*) undersown in winter wheat and re-sown after wheat harvest (“clover”), (ii) hairy vetch (*Vicia villosa*) as legume cover crop (“vetch”), (iii) oilseed radish (*Raphanus sativus*) as non-legume cover crop (“radish”), and (iv) fallow (“control”).

Winter wheat (*Triticum aestivum*) was sown either as pure crop (vetch, radish and control cover crop treatments) or intercropped with subterranean clover (clover treatment). After wheat harvest, all three winter cover crops (clover, vetch and radish) were sown. In the next spring (end of April/begin of May), cover crops were either terminated by tillage (ct) or by applying glyphosate in the nt treatment. Maize (*Zea mays*) was then sowed in the end of May.

For winter wheat residues and cover crop shoots, five subsamples were taken on 16 plots (in a W-scheme) and pooled in April 2015. Cover crop samples were composed of shoot material of the cover species (clover, vetch or radish) or of a composite sample of weeds present on the control plot. All cover crop samples were washed with PBS-S buffer as described above. Residue and cover crop samples were lyophilized for 48 h and ground in a ball mill for 1.5 min with 30 Hz for DNA extraction. Maize grains were harvested in two rows along 7 m length each with an adapted plot-sized combine harvesters. Maize grains were dried at 60°C, grinded and used for DNA extraction. The DNA samples served as templates for PCR with the primer pair *ITS1f:LR6* (Supplementary Table S2). Amplicons were subsequently analyzed with SMRT sequencing as described above.

### Statistical Analysis

All analyses were performed using R (R Core Team, 2016), and the specific R and Bioconductor packages (as indicated below). Abundance of individual taxa was normalized by the sampling depth of each sample and expressed as percentage relative abundance. The correlation between *Fusarium* communities determined by sequencing and by SHT was tested using Spearman rank order correlation.

Rarefaction analysis was performed in QIIME on the by abundance filtered OTU table (exported from R for this purpose) and on the original OTU table (Supplementary Figure S1). The analysis of α- and β-diversity along the crop rotation in the OSCAR field trial were performed on rarefied samples using the Bioconductor package phyloseq (McMurdie and Holmes, 2013). Differences in α-diversity measures (OTU richness and Shannon Index) were tested using One- or Two-way ANOVA implementing the factors *sample type*, *cover crop* and *tillage* if applicable for the group of samples (the first only for comparison over all sample types, the latter only for maize grain samples). To quantify the major variance factors of β-diversity along the crop rotation, we performed a multivariate analysis of fungal diversity including a testing for the experimental factors by permutational analysis of variance (PERMANOVA) followed in case of significant effects by a constrained canonical analysis of principal coordinates (CAP; Anderson and Willis, 2003). Finally, a characterization of the taxa responsible for the multivariate patterns based indicator species analysis was performed (De Cáceres and Legendre, 2009). All *p*-values were adjusted for multiple testing with the FDR correction using the Benjamin–Hochberg method (Benjamini and Hochberg, 1995).

## Results

### Taxonomic Resolution among *Fusarium* Species Based on the Ribosomal Operon

In a first step, we evaluated *in silico* different segments of the ribosomal operon to define the marker region for best possible discrimination among *Fusarium* isolates at species level. The combined use of ITS and LSU offered a higher resolution compared to single segments (**Figure 1B**). The ca. 1.6 kb long segment spanning the entire ITS segment and the highly variable segments D1, D2, and D3 of the LSU could separate 23 unique sequences and differentiate 13 out of 14 *Fusarium* species *s.l.* tested. Only the species *F. avenaceum* and *F. tricinctum* were not discriminated properly and thus clustered into the same *Fusarium* OTU (referred to as ‘*F.ave/tri*’). Moreover, we identified two distinct sequence groups in various isolates of *F. equiseti* that are all classified as one species using morphological characteristics. Overall, the 44 tested Swiss *Fusarium* isolates clustered into 14 distinct *Fusarium* OTUs with the new methodology. While the combined ITS-LSU segment discriminated *Fusarium* species correctly to morphological assessed species boundaries, the single ITS or D1–D2 segments were insufficient to resolve on this level (**Figure 1B**). The ITS segment failed to separate *F. graminearum* and *F. culmorum*, and *F. subglutinans* and *F. verticillioides* isolates. The D1–D2 segment failed to discriminate *F. crookwellense* and *F. culmorum*, and *F. proliferatum* and *F. subglutinans*. Moreover, the ITS segment on its own showed clusters not adherent to morphological assessed species boundaries and thus failed to group all isolates of a single species into the same OTU (e.g., see clustering of *F. graminearum and F. crookwellense* or of *F. subglutinans* and *F. verticillioides*; **Figure 1B**). Based on these results, we decided to move forward with an amplicon spanning the entire ITS region and highly variable parts of the LSU (D1, D2, and D3) to profile *Fusarium* communities with the highest resolution based on the ribosomal operon segments considered.

### Validation of *Fusarium* Abundance As Revealed by Community Quantification

We tested four different PCR primer pairs for the development of a sequencing-based method to measure *Fusarium* communities. We describe the corresponding sequencing effort in Supplementary Table S4 (Library Mthd1). We compared the primers for their specificity to amplify fungi and their coverage of the genus *Fusarium* on three different sample types; maize grains heavily infected by *Fusarium*, shoot material of winter wheat and a composite sample of co-occurring weeds. The primer’s specificity to amplify fungal signals was highly dependent on the forward primer and sample origin (Supplementary Figure S2A). In the maize grain and wheat shoot samples, all reads were assigned to the fungal kingdom by both forward primer, however, in the composite weed sample a substantial number of plant sequences were detected. Regardless the reverse primer used, pairs including *ITS1F* amplified less than 2.5% plant reads, whereas primer pairs including *fITS7* amplified up to 44% plant reads (Supplementary Figure S2A). The taxonomic analysis at genus level revealed differences between tested primer pairs in their coverage of the genus *Fusarium* (dark red; Supplementary Figure S2B). In the weed composite sample, only primer pairs including the reverse primer *LR6* were able to detect *Fusarium* OTUs. Similarly, in the winter wheat sample, only the primer pair *ITS1f:LR6* was able to detect *Fusarium* OTUs (dark red; Supplementary Figure S2B). Overall, the primer pair *ITS1f:LR6* showed highest fungal specificity in combination with the best coverage of *Fusarium* taxa and was therefore chosen for further analysis.

**FIGURE 2:**
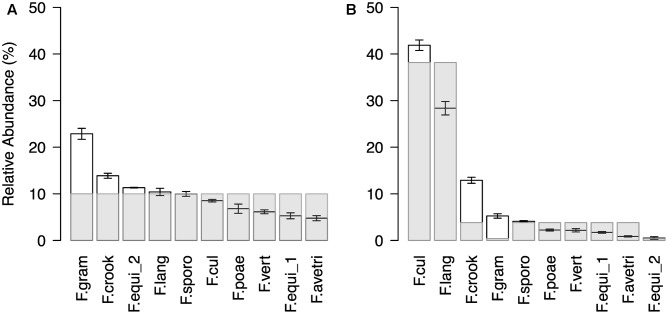
Community profile of *Fusarium* mock communities. Ranked mean OTU abundances (±SE, *n* = 3) of **(A)** even and **(B)** staggered mock communities composed of ten different isolates (*F. avenaceum, F. crookwellense, F. culmorum, F. equiseti* (two isolates), *F. graminearum, F. langsethiae, F. poae, F. sporotrichioides, F. verticillioides;* Abbreviation of *Fusarium* OTUs as described in **Figure 1**). In the even mock community, the *Fusarium* isolates were equally represented in the template mixture. In the staggered mock community, the isolate of the species *F. culmorum* (*F.cul*) and *F. langsethiae* (*F.lang*) were ten times higher concentrated and an isolate of *F. equiseti* (*F.equi_2*) and *F. graminearum* (*F.gram*) were ten times lower concentrated as the remaining six isolates. Light gray bars indicated expected abundances.

In a next step, we confirmed the method’s technical reproducibility by comparing three replicated profiles of the same sample (see Supplementary Results for details; Supplementary Figure S3) and assessed accuracy in reproducing pre-defined *Fusarium* mock communities (**Figure 2**). The artificial mock communities consisted of DNA mixtures of ten *Fusarium* isolates, which were either assembled in even ratios or with a staggered distribution. In general, the sequencing profiles of the tested mock communities reflected well the expected abundances of the mixed *Fusarium* (**Figure 2**). For the even mock community, an average OTU abundance of ten per cent would be expected for each of the ten *Fusarium* isolates (**Figure 2A**). The measured mean OTU abundances did not differ more than around twofold from the expected values (*rho* = 0.7, *p* = 0.02). The OTU *F.gram* was consistently overrepresented, whereas the OTUs *F.vert, F.poae, F.equi_1*, and *F.ave/tri* tended to be underrepresented. In the staggered mock community, the two most abundant OTUs *F.cul* and *F.lang* corresponded to the enriched DNA templates, while only one of the two OTUs (*F.equi_2*) with a reduced DNA concentration belonged to the two rarest OTUs (*rho* = 0.61, *p* = 0.06; **Figure 2B**). We noted again that the OTU *F.gram* was overrepresented compared to the expected value and this was also true for the OTU *F.crook*. Overall, the test revealed a reliable display of the artificial *Fusarium* communities.

**FIGURE 3:**
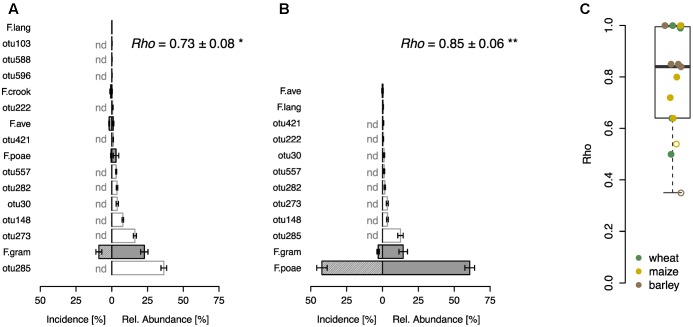
Comparison of community profiling based on sequencing and cultivation-based seed health test on different grain samples. Community profiling on a wheat field trial where plots were either not inoculated **(A)** or inoculated with *F. poae* **(B)**. Bars represent ranked mean OTU abundances determined by sequencing based community profiling (±SE, *n* = 4; gray bars indicate *Fusarium* OTUs abbreviated as described in **Figure 1**, white bars represent other fungal OTUs). Hatched bars represent incidence of *Fusarium* species determined by cultivation-based seed health test (±SE, *n* = 4), other fungal taxa could not be detected (nd). Spearman correlation index (*rho-*values) are given in the respective upper right corner demonstrating the statistical dependence between the ranking of *Fusarium* taxa of the two compared methods (^∗^*p* < 0.05, ^∗∗^*p* < 0.01). **(C)** Spearman correlation analysis (*rho-*values) of *Fusarium* taxa on wheat, maize and barley samples (*n* = 15). Closed circles indicate a significant correlation (*p* < 0.05), open circles are not significant.

In a further step, we compared the performance of the sequence-based *Fusarium* profiling with the cultivation-dependent SHT. To this end a total of 23 field samples were assessed with both methods. Eight of these samples originated from a field experiment with wheat, where *F. poae* was inoculated and we found highly significant correlations between the abundance of *Fusarium* taxa assessed by the two different methods in control and inoculated treatments (**Figures 3A,B**). Both methods revealed that in the control treatment *F. graminearum* was the most abundant *Fusarium* taxon detected, although its incidence was rather low, and other fungal OTUs showed higher relative abundances (**Figure 3A**). In the inoculated samples, both methods determined a heavy infection with *F. poae* as reflected in a high incidence score and high relative abundance of the OTU *F.poae* (**Figure 3B**). For the other tested samples, including grain samples from barley, maize and wheat, we also found sound correlations between *Fusarium* taxa determined by SHT or sequencing with a mean Spearman’s *rho* of 0.78 ± 0.05 (mean ± SE, *n* = 15; **Figure 3C**). Only two of the 15 analyzed monitoring samples exhibited non-significant relationships between the two methods.

### *Fusarium* Abundance and Distribution along a Wheat-Maize Crop Sequence

**FIGURE 4:**
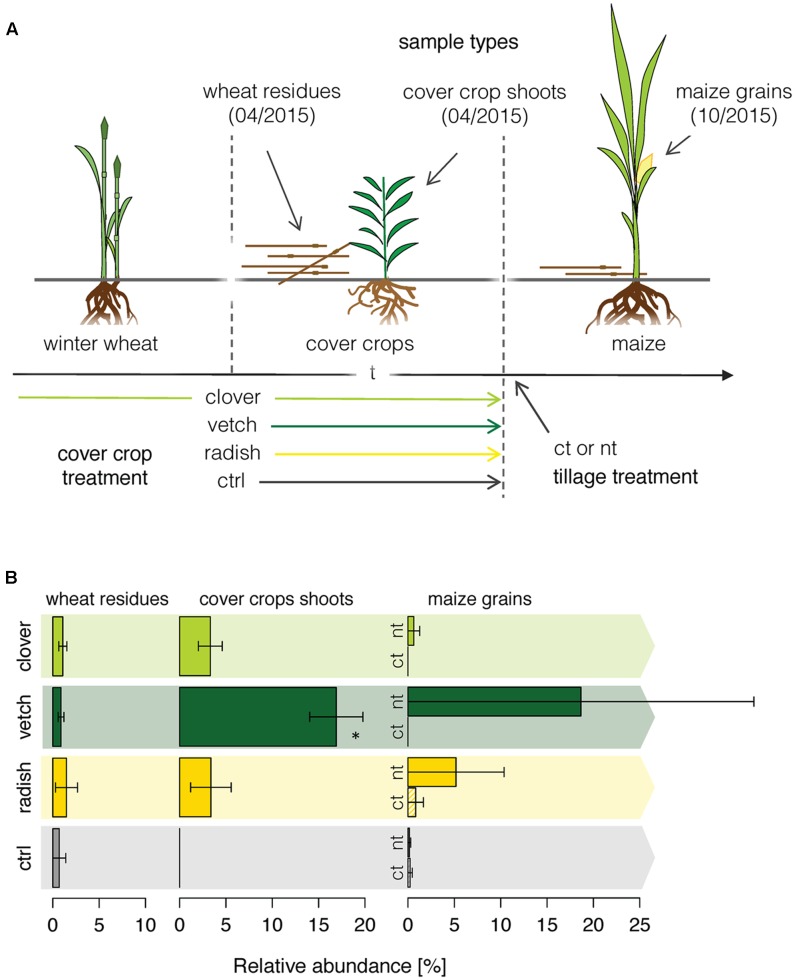
OSCAR field experiment investigating the effect of cover crop and tillage treatments on the diversity and dispersal of *Fusarium* fungi along a wheat-maize crop sequence. **(A)** Cover crops were applied either as undersown subterranean clover in winter wheat and re-sown after wheat harvest (clover), as leguminous (vetch) or non-leguminous (radish) cover crop both sown after wheat, and green fallow as control (ctrl). Tillage treatments were conventional inversion tillage (ct) and no-tillage (nt) applied before maize in the second year of the experiment. Each treatment was replicated 4 times. **(B)** Dispersal of the *Fusarium* OTU *F.ave/tri* along the crop rotation. Relative abundance of the OTU *F.ave/tri* (±SE, *n* = 4) in winter wheat residues, cover crop shoots and maize grains as affected by cover crop and tillage (tillage effect only in maize) treatments. Stars above bars indicate a significant difference within a sample type.

Finally, we applied the newly developed methodology to a field experiment, where we traced the *Fusarium* community composition in the phyllosphere of different crops along a wheat-maize crop sequence. We investigated how the different cover crop and tillage treatments affected the *Fusarium* abundance along a wheat-maize sequence to get insights in the dispersal of specific *Fusarium* taxa (**Figure 4A**). *Fusarium* communities were analyzed in the wheat residues and in the different cover crop species before maize was sown, and finally in the grains of the subsequent maize crop. Overall, the relative abundances of *Fusarium* taxa were low and the fungal communities were dominated by other OTUs (Supplementary Figure S5). In wheat residues and cover crop shoots, only the *Fusarium* OTU *F.ave/tri* was detected, whereas in maize nine different *Fusarium* OTUs were detected of which *F.ave/tri*, *F.prol*, *F.gram, F.cul*, and *F.oxy* were among the 25 most abundant fungal OTUs (Supplementary Figure S5C). However, no significant difference in *Fusarium* taxa richness between different treatments (cover crop and tillage) was found within maize grains (data not shown).

*F.ave/tri* was the only *Fusarium* OTU that was detected throughout the wheat-maize crop sequence and thus only its dispersal could be analyzed for effects of cover crop or tillage treatments. While the relative abundance of the OTU *F.ave/tri* was rather low and similar among all treatments in wheat residues, it was substantially affected by the treatments in cover crops exhibiting a more than eightfold increase in vetch compared to the other treatments (**Figure 4B**). In maize grains, the abundance of *F.ave/tri* was highly variable and not even detected in some of the samples, therefore, treatment related patterns could not be statistically confirmed. Nonetheless, means of all no-tillage samples tended to be higher than under plowed conditions, particularly, the combination of no-tillage and vetch as cover crop exhibited the highest mean abundance (**Figure 4B**).

### Fungal Community Dynamics along a Wheat-Maize Crop Sequence

**FIGURE 5:**
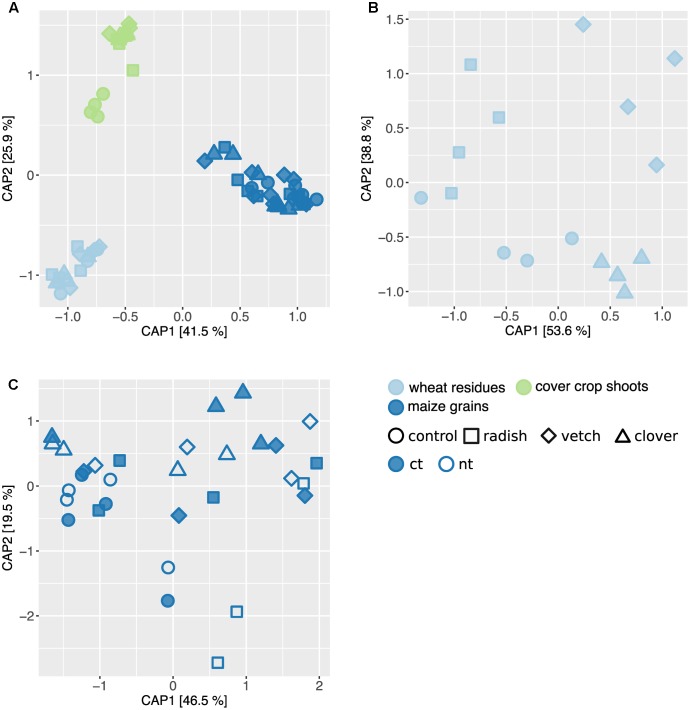
Effect of sample type and treatments on fungal community structure along a wheat-maize cropping sequence. CAP ordinations of Bray–Curtis similarities calculated based on relative OTU abundances showing major differences among fungal communities induced by sample types and different treatments. **(A)** The constrained factors (sample type ^∗^ cover crop treatment) explain 58% of variance (*p* = 0.001) between the fungal communities along the cropping sequence. **(B)** In wheat residues, the constrained factors (cover crop treatment, conditioned for block effect) explain 26% of variance (*p* = 0.029) between the fungal communities. **(C)** In maize grains, the constrained factors (cover crop and tillage treatment) explain 23% of variance (*p* = 0.49) between the fungal communities. Percentages indicated for each axis referring to their contribution to the constrained variation.

**FIGURE 6:**
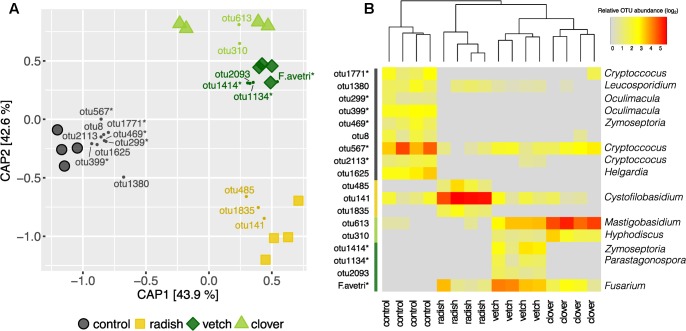
Effect of cover crops on its associated fungal communities. **(A)** CAP ordination of Bray–Curtis similarities calculated based on relative OTU abundances showing major differences among fungal communities induced by different cover crop treatments. The constrained factors (cover crop treatment) explain 77% of variance (*p* = 0.001) between the fungal communities. Percentages indicated for each axis referring to their contribution to the constrained variation. Cover crop sensitive OTUs are displayed in the corresponding color (clover: light green, vetch: dark green, radish: yellow and control: black). **(B)** Relative abundances of cover crop sensitive OTUs. Dendrogram is based upon Bray–Curtis distances among fungal communities in cover crop samples. Stars next to OTU names indicate potential plant pathogenic phylogenetical background. Taxonomical assignment (genus) of cover crop sensitive OTUs is indicated on the right if available.

In addition to *Fusarium*, we assessed the overall composition of fungal communities by means of the universal fungal approach (see Supplementary Results for further information) using SMRT sequencing. We found substantial differences in fungal community composition along the wheat-maize sequence, mainly between the different sample types (wheat residues, cover crop shoots and maize grains; **Figure 5A**). Further, we investigated the effect of cover crop and tillage treatments (the latter only for maize) on fungal communities separately for the different sample types. In wheat residues collected at the end of cover cropping period, the fungal communities were only slightly affected by cover crop treatments (**Figure 5B**). In contrast, the analysis of the cover crop shoots revealed species-specific fungal communities in the phyllosphere of different cover crops (**Figure 6A**). The influence of cover crop treatments to β-diversity was large (CAP revealed 77% of variance is explained by the constrained factor “cover crop treatment”; **Figure 6A**) and all four treatments harbored significantly different fungal communities based on pair-wise comparison (Supplementary Table S7). Finally, we did not find an effect of the factors cover crop nor tillage to have an influence on the fungal communities in maize grain samples (**Figure 5C**).

Complementary to the examination of diversity patterns, we also identified treatment-sensitive OTUs based on indicator statistics (see Supplementary Results for further information). However, we found only treatment-sensitive taxa in the cover crop samples (**Figure 6B** and **Table 1**). Remarkably, vetch and weeds sampled on the control plots harbored several treatment-sensitive OTUs linked to plant pathogens. One of these is the *Fusarium* OTU *F.ave/tri* only characteristically associated to vetch. Moreover, OTUs assigned to the wheat pathogens *Zymoseptoria tritici* and *Parastagonospora nodorum*, both causing wheat leaf blotch, were more abundant in vetch samples (**Figure 6B** and **Table 1**). In addition, the control plots harbored also several potential plant pathogens including OTUs assigned to *Z. tritici* and *Oculimacula yallundae* (**Figure 6B** and **Table 1**). Taken together, different cover crop practices exhibited distinct fungal communities of which vetch and weeds on the control plots harbored the highest phytopathological risk.

**Table 1:** Taxonomic assignments of treatment sensitive taxa in cover crops.

	Phylum	Class	Order	Family	Genus	Species	OTU	Plant association
Clover	Ascomycota	Leotiomycetes	Helotiales	Hyaloscyphaceae	*Hyphodiscus*	*H. hymeniophilus*	otu310^∗^	–
	Basidiomycota	Microbotryomycetes	Leucosporidiales	Leucosporidiaceae	*Mastigobasidium*	*M. intermedium*	otu613	Epiphyte^1^
								
Vetch	Ascomycota	Dothideomycetes	Capnodiales	Mycosphaerellaceae	*Zymoseptoria*	*Z. tritici*	otu1414^∗^	Pathogen (wheat)^2^
	Ascomycota	Dothideomycetes	Pleosporales	Phaeosphaeriaceae	*Parastagonospora*	*P. nodorum*	otu1134^∗^	Pathogen (wheat)^3^
	Ascomycota	Sordariomycetes	Glomerellales				otu2093^∗^	–
	Ascomycota	Sordariomycetes	Hypocreales	Nectriaceae	*Fusarium*	*F. tricinctum*	*F.ave/tri*	Pathogen (cereals)^4^
								
Radish	Ascomycota	Dothideomycetes	Pleosporales	Pleosporaceae			otu485^∗^	–
	Ascomycota	Dothideomycetes	Pleosporales	Pleosporaceae			otu1835^∗^	–
	Basidiomycota	Tremellomycetes	Tremellales	Cystofilobasidiaceae	*Cystofilobasidium*	*C. capitatum*	otu141^∗^	Epiphyte^5^
								
Control	Ascomycota	Dothideomycetes	Capnodiales	Mycosphaerellaceae	*Zymoseptoria*	*Z. tritici*	otu469^∗^	Pathogen (wheat)^2^
	Ascomycota	Dothideomycetes	Pleosporales				otu8	–
	Ascomycota	Leotiomycetes	Helotiales	Incertae sedis	*Oculimacula*	*O. yallundae*	otu299	Pathogen (wheat)^6^
							otu399	
	Ascomycota	Leotiomycetes	Helotiales	Incertae sedis	*Helgardia*		otu1625	
	Basidiomycota	Microbotryomycetes	Leucosporidiales	Leucosporidiaceae	*Leucosporidium*	*L. golubevii*	otu1380	Epipyte^7^
	Basidiomycota	Tremellomycetes	Tremellales	Tremellaceae	*Cryptococcus*		otu1771	–
	Basidiomycota	Tremellomycetes	Tremellales	Tremellaceae	*Cryptococcus*	*C. victoriae*	otu567	Epipyte^8^
	Basidiomycota	Tremellomycetes	Tremellales	Tremellaceae	*Cryptococcus*	*C. neoformans*	otu2113^∗^	Pathogen (Arabidopsis)^9^


*Taxonomy was assigned by making use of the UNITE or RDP (indicated with ^∗^) database (for details see Material and Methods). Indications on the ecology of plant association was based on the following references; Nakase and Suzuki, 1987^1^; King et al., 1983^2^; Solomon et al., 2006^3^; Parry et al., 1995^4^; Glushakova and Chernov, 2010^5^; Crous et al., 2003^6^; Sampaio et al., 2003^7^; Montes et al., 1999^8^; Xue et al., 2007^9^, “–”: no plant association reported.*

## Discussion

### Suitability of the Ribosomal Operon to Discriminate among *Fusarium* Species

The first objective of this study was the development of a sequencing-based methodology that robustly quantifies *Fusarium* taxa in shoot and grain samples. In addition, we aimed to develop an approach allowing to focus on *Fusarium*, but also assessing other fungi associated to crops. By making use of a long amplicon spanning the entire ITS and the 3′-end of the LSU including the highly variable segments D1, D2, and D3 in combination with clustering against a comprehensive reference sequence database, we were able to determine *Fusarium* beyond the morphological species resolution based on the prime target region for fungal barcoding, the ribosomal operon (Seifert, 2009). Moreover, the present study represents the first attempt to combine the highly variable segments ITS and D1–D3 in a long amplicon for fungal profiling in high resolution, both displaying most favored segments of the ribosomal operon for fungal barcoding (Schoch et al., 2012; Nilsson et al., 2013). The combination of these two segments generates an additive value for fungal community profiling as we could demonstrate for the genus *Fusarium*. The combined segment allowed to discriminate between the tested *Fusarium* taxa, while the two segments on their own were less powerful in terms of resolution, and moreover, in terms of species assignments of individual isolates. The D1–D2 segment revealed mainly a poor taxonomic resolution as reported earlier (O’Donnell et al., 2008b). In contrast, the ITS segment on its own revealed weaknesses in clustering isolates to the corresponding *Fusarium* species. This might be caused by paralogous sequences in the ITS segment known to occur within *Fusarium* species and making taxonomic assignments based on the ITS segment difficult (Waalwijk et al., 1996; O’Donnell and Cigelnik, 1997).

However, using the sequence combining the ITS and D1–D3 segment, only the two of the tested *Fusarium* species *s.l.*, *F. avenaceum* and *F. tricinctum*, have not been separated properly. This is not surprising as both belong to a large species complex known to be not easily distinguishable with ribosomal tools (Yli-Mattila et al., 2002). In contrast, the species *F. equiseti* is separated into two clusters based on our analysis. This agrees with previous studies on the phylogeny of *F. equiseti* revealing high genetic diversity and two distinct clusters within this species (O’Donnell et al., 2009; Marín et al., 2012).

In conclusion, the newly developed methodology is able to identify *Fusarium* down to morphological species level or even beyond. This ability displays an important feature for a diagnostic tool as conclusions on the phytopathological risk of *Fusarium* taxa can only be drawn on species level, because pathogenicity as well as mycotoxin production largely differs among *Fusarium* species and even among isolates (Appel and Gordon, 1996; Bottalico and Perrone, 2002). For instance, among isolates of the species *F. graminearum* and *F. poae*, different chemotypes exhibiting distinct mycotoxin profiles have been identified (Jennings et al., 2004; Stenglein, 2009; Stȩpień et al., 2011; Pasquali et al., 2016). Moreover, Marín et al. (2012) found different mycotoxin profiles among two isolate clusters of *F. equiseti* which may be coherent with the two distinct *F. equiseti* OTUs we found in the present study (**Figure 1B**). However, recent development on *Fusarium* phylogeny has revealed that the morphological species concept misses parts of the phylogenetic diversity of this genus (O’Donnell et al., 2013). Hence, the taxonomical resolution assessed with the methodology introduced in this study might partially underrepresent the *Fusarium* diversity. Nevertheless, other recent high-throughput approaches did not exceed the taxonomical resolution of our study and also resolve within morphological species boundaries (Karlsson et al., 2016).

### Validation of the Diagnostic Power of the Newly Developed Methodology

Besides its resolution, the newly developed methodology showed its diagnostic power in the validation based on mock communities and in the comparison to the assessment of *Fusarium* communities with the cultivation-based SHT. In the mock communities, we found a twofold difference between the most and the least abundant species in the equal community displaying a similar accuracy as in other sequencing-based *Fusarium* profiling methods (Karlsson et al., 2016). Particularly, *F. graminearum* was overrepresented in the mock communities. It is important to note that the mock communities were just equilibrated by mixing equal amounts of genomic DNA, and thus, differences in copy numbers of the ribosomal operon (Rodland and Russell, 1982), and in addition, differences in genome size could have resulted in various ratios of the ribosomal operon to total DNA concentration. For example, the genome of *F. graminearum* has been estimated to be relatively small compared to *F. oxysporum* and *F. verticillioides* (Ma et al., 2010), thus *F. graminearum* exhibits relative higher number of copies of the genome with equal DNA amount. Overall, however, the quantitative relationship between species was well displayed with the newly developed methodology.

Similarly, the comparison with SHT revealed a high correlation between *Fusarium* communities assessed with the two different approaches. This is somewhat surprising considering the inherent differences between cultivation- and sequence-based approaches, but also demonstrates the robustness of both methods. More importantly, both approaches were able to detect the epidemic threat of *F. poae* in the inoculation field experiment, thus demonstrating the usability of both approaches to track taxa-specific FHB causing species in grain samples. In addition, the newly developed methodology allows a perspective beyond *Fusarium* fungi as other fungal species are also assessed, which can reveal further phytopathological risks as revealed for the OSCAR field experiment in the current study.

A major drawback of the newly developed methodology is its dependency on a suitable reference sequence database to reach highest taxonomical resolution. We established a comprehensive reference sequence database starting with 44 *Fusarium* isolates collected in Swiss agro-ecosystems. This resulted in a reference sequence database of 23 unique sequences covering a large range of *Fusarium* species *s.l*. occurring in Swiss cereal and maize fields (Dorn et al., 2009; Vogelgsang et al., 2011). In the herein presented test case, the OSCAR field experiment, all OTUs assigned to the genus *Fusarium* were OTUs clustering to the reference sequence database. This illustrates that the *Fusarium* reference sequence database used covers the major diversity of *Fusarium* taxa in Swiss cereal and maize fields. Temporarily, the newly developed methodology is relying on hand-curated reference sequence database spanning the ITS and D1–D3. The current fungal sequence databases comprise the ITS (UNITE; Kõljalg et al., 2013), or just the D1-D2 segment alone (RDP; Cole et al., 2014), but there is not yet a sequence database available combining these two potent target regions. Nevertheless, due to the increased use of SMRT sequencing and the improved throughput of Pacific Bioscience with the newly developed ‘SEQUEL’ system^2^, more reference sequence databases comprising longer sequences will probably be developed in the near future, which will help to overcome the limitation of the newly developed methodology.

### *Fusarium* Diversity and Distribution along the Wheat-Maize Crop Sequence

The focus of the herein introduced methodology lies on *Fusarium* fungi causing one of the globally most noxious plant diseases, particularly in small grain cereals and maize (Parry et al., 1995; Desjardins, 2003). Overall, the abundance of *Fusarium* was rather low and other taxa dominated the fungal communities. Only in maize, a considerable *Fusarium* diversity comprised of nine different species could be detected. This is comparable to the species richness defined in grain samples in another sequence-based *Fusarium* community study in winter wheat (Karlsson et al., 2017). However, earlier studies have found up to 20 species to be associated with cereals (Xu et al., 2005; Bourdages et al., 2006; Dorn et al., 2009). In contrast to maize, only a single *Fusarium* OTU, *F.ave/tri*, was detected in wheat residues and shoots of cover crops and weeds. This very low species richness is unexpected as most of the *Fusarium* species found in maize grains are also able to colonize crop residues (Munkvold, 2003; Xu and Nicholson, 2009), and have been isolated from a wide range of other plants including different weed species (Jenkinson and Parry, 1994; Lager and Wallenhammar, 2003). Generally, however, it is important to note that the low extent of *Fusarium* fungi found in the investigated field experiment is probably related to the dry weather in 2014/2015 that resulted in an overall low incidence of FHB in Switzerland (Schöneberg et al., 2016).

The dispersal of only *Fusarium* OTU *F.ave/tri* could be analyzed along the crop sequence in this study. This OTU comprises the *Fusarium* species *F. avenaceum*, which is along with *F. graminearum* one of the predominant pathogens of FHB (Parry et al., 1995; Xu and Nicholson, 2009). The abundance of the *Fusarium* OTU *F.ave/tri* revealed interesting effects of cover crop and tillage treatments on its dispersal along the crop sequence. While its abundance was not affected by any treatment in wheat residues, a substantial higher abundance of *F.ave/tri* was found in vetch compared with the other cover crop species and weeds of the control treatment. This interesting pattern was less explicit in maize grains, where the abundance of *F.ave/tri* was highly variable. Nonetheless, the reported mean abundances of *F.ave/tri* in maize indicate that the cover crop vetch in combination with no tillage has the potential to increase the dispersal along the crop sequence. This finding is supported by an earlier study showing that *F. avenaceum* was more frequently associated with cereals in sequence with leguminous crops under reduced tillage (Fernandez et al., 2007). It is generally known that prevalence of *Fusarium* is greatest for no-tillage and reduced under conventional tillage (Bateman et al., 2007; Dill-Macky, 2008). Up to now, this was mainly attributed to the fact that most of *Fusarium* species depend on proliferation on crop residues, which serve as inoculum for the following crop (Xu and Nicholson, 2009). However, we demonstrate here that certain cover crops act as alternative hosts of *Fusarium* and hence could represent another inoculum source for FHB in a crop sequence.

### Distinct Fungal Communities along a Wheat-Maize Crop Sequence

The application of the newly developed methodology to track crop-associated fungi along a wheat-maize sequence depicted at first sight that the three sample types, wheat residues, cover crop shoots and maize grains, harbored structurally highly distinct fungal communities. This is not surprising as the sample types represented different substrates, including plant debris and living plants, and additionally originate from different plant species. It has been broadly shown that the species of a plant displays a major driver shaping microbial communities inhabiting its phyllosphere (Redford et al., 2010; Rastogi et al., 2012; Kembel and Mueller, 2014). Moreover, the herein reported impact of sample type has also a temporal component as the sample types have not been harvested at the same time. Temporal effects have also shown to significantly affect microbial community structures in agro-ecosystems (Lukow et al., 2000; Lauber et al., 2013).

While we found substantial differences between sampling types, the impact of the different cropping measures on fungal communities was not apparent throughout the crop sequence. The cover crop treatment was only a significant driver of fungal community structure in cover crop shoot samples. It is important to note that in the sample type cover crop shoots, different plant species have been sampled in the different treatments, which can be a major driver of the microbial community structure of the phyllosphere as we just have observed (see above). The fungal communities of the leguminous cover crops, clover and vetch, clustered closer together compared to the other two treatments hosting phylogenetical more distant plant species. This finding is supported by earlier studies indicating that phylogenetic distance of host plants contributes to diversification of associated microbial communities (Leff and Fierer, 2013; Schlaeppi et al., 2014).

Although we could report substantial differences in the fungal communities of cover crops, this had no impact on the fungal communities in the subsequent crop maize. Fungal communities of maize grains were not affected by cover crop or tillage treatments. Hence, we must conclude that the distinct fungal communities associated to cover crops have not been spread or established in the subsequent maize under the investigated condition and that the phyllosphere of previous crops may not be the main source for the recruitment of the phyllosphere community of the current crop. Indeed, so far it is still unclear how microbial communities in the phyllosphere establish. Further studies have to elucidate how the recruitment *via* other plants, soil or air contribute to the establishment of phyllosphere communities (Vorholt, 2012).

### Phytopathological Risks of Different Cover Crop Treatments

Several cover crop treatment-sensitive fungal taxa were identified. The fungal communities of vetch and weeds sampled in the control treatment were characterized by several OTUs assigned to plant pathogens, while clover and radish treatments did harbor commensal plant epiphytes. Particularly vetch revealed a high potential to act as alternative host for noxious plant pathogens featuring besides the *Fusarium* OTU *F.ave/tri* also an OTU assigned to *Zymoseptoria tritici*, a major fungal plant pathogen (Dean et al., 2012). The weed species of the control treatment hosted also plant pathogens, particularly of wheat (*Z. tritici* and *Oculimacula yallundae*). It is important to note that weed samples also include volunteer wheat regrown on the control plots (up to 30% of surface cover; data not shown), which may explain the prominent role of wheat pathogens in the weed sample. In summary, the distinct fungal communities found in cover crops feature diverging phytopathological risks for a crop sequence by acting as alternative host of noxious plant pathogens. However, the phytopathological risks found in cover crops were not apparent in the subsequent crop maize of the current experiment (see above).

## Conclusion

We tested and developed a new sequencing based methodology for fungal community profiling that allows to detect fungal pathogens from the genus *Fusarium* beyond the morphological species level based on the highly variable segments of the ribosomal operon. We demonstrate that the herein introduced method is a powerful diagnostic tool to track *Fusarium* communities in combination with other fungal pathogens and non-pathogenic fungi associated to crop species. By analyzing a field experiment, we revealed that cover crops differ in their potential to act as alternative hosts for *Fusarium*, and moreover to host further fungal plant pathogens including *Z. tritici*. Hence, the choice of cover crops with respect to their phytopathological risk displays an important feature to improve the control of FER and FHB epidemics along a crop sequence.

## Author Contributions

FW and MvdH designed the study. SV contributed selected *Fusarium* isolates and information of the respective cropping histories. FW and RW collected the data. RW was responsible for the OSCAR field experiment. FW and AH performed the molecular work. FW and KS analyzed the data. FW wrote the manuscript and all co-authors contributed substantially to revisions.

## Conflict of Interest Statement

The authors declare that the research was conducted in the absence of any commercial or financial relationships that could be construed as a potential conflict of interest.

## Footnotes

1 http://www.fgcz.ch

2 http://www.pacb.com/products-and-services/pacbio-systems/sequel/

## References

[B1] Agler M. T. Ruhe J. Kroll S. Morhenn C. Kim S. T. Weigel D. (2016). Microbial hub taxa link host and abiotic factors to plant microbiome mariation. *PLOS Biol.* 14:e1002352. 10.1371/journal.pbio.1002352 26788878 PMC4720289

[B2] Anderson M. J. Willis T. J. (2003). Canonical analysis of principal coordinates: a useful method of constrained ordination for ecology. *Ecology* 84 511–525. 10.1890/0012-9658(2003)084[0511:CAOPCA]2.0.CO;2

[B3] Appel D. J. Gordon T. R. (1996). Relationships among pathogenic and nonpathogenic isolates of *Fusarium oxysporum* based on the partial sequence of the intergenic spacer region of the ribosomal DNA. *Mol. Plant Microbe Interact.* 9 125–138. 10.1094/MPMI-9-0125 8820752

[B4] Balajee S. A. Borman A. M. Brandt M. E. Cano J. Cuenca-Estrella M. Dannaoui E. (2009). Sequence-based identification of *Aspergillus*, *Fusarium*, and Mucorales species in the clinical mycology laboratory: where are we and where should we go from here? *J. Clin. Microbiol.* 47 877–884. 10.1128/JCM.01685-08 19073865 PMC2668331

[B5] Bateman G. L. Gutteridge R. J. Gherbawy Y. Thomsett M. A. Nicholson P. (2007). Infection of stem bases and grains of winter wheat by *Fusarium culmorum* and *F. graminearum* and effects of tillage method and maize-stalk residues. *Plant Pathol.* 56 604–615. 10.1111/j.1365-3059.2007.01577.x

[B6] Benjamini Y. Hochberg Y. (1995). Controlling the false discovery rate: a practical and powerful approach to multiple testing. *J. R. Stat. Soc. Ser. B* 57 289–300.

[B7] Berendsen R. L. Pieterse C. M. J. Bakker P. A. H. M. (2012). The rhizosphere microbiome and plant health. *Trends Plant Sci.* 17 478–486. 10.1016/j.tplants.2012.04.001 22564542

[B8] Bokulich N. A. Subramanian S. Faith J. J. Gevers D. Gordon J. I. Knight R. (2012). Quality-filtering vastly improves diversity estimates from Illumina amplicon sequencing. *Nat. Methods* 10 57–59. 10.1038/nmeth.2276 23202435 PMC3531572

[B9] Bottalico A. Perrone G. (2002). Toxigenic *Fusarium* species and mycotoxins associated with head blight in small-grain cereals in Europe. *Eur. J. Plant Pathol.* 108 611–624. 10.1023/A:1020635214971 15645174

[B10] Bourdages J. V. Marchand S. Belzile F. J. Rioux S. (2006). Diversity and prevalence of *Fusarium* species from Quebec barley fields. *Can. J. Plant Pathol.* 28 419–425. 10.1080/07060660609507315

[B11] Caporaso J. G. Kuczynski J. Stombaugh J. Bittinger K. Bushman F. D. Costello E. K. (2010). QIIME allows analysis of high-throughput community sequencing data. *Nat. Methods* 7 335–336. 10.1038/nmeth.f.303 20383131 PMC3156573

[B12] Cole J. R. Wang Q. Fish J. A. Chai B. McGarrell D. M. Sun Y. (2014). Ribosomal database project: data and tools for high throughput rRNA analysis. *Nucleic Acids Res.* 42 633–642. 10.1093/nar/gkt1244 24288368 PMC3965039

[B13] Crous P. W. Groenewald J. Z. E. Gams W. (2003). Eyespot of cereals revisited: ITS phylogeny reveals new species relationships. *Eur. J. Plant Pathol.* 109 841–850. 10.1023/A:1026111030426

[B14] De Cáceres M. Legendre P. (2009). Associations between species and groups of sites: indices and statistical inference. *Ecology* 90 3566–3574. 10.1890/08-1823.1 20120823

[B15] Dean R. Van Kan J. A. L. Pretorius Z. A. Hammond-Kosack K. E. Di Pietro A. Spanu P. D. (2012). The top 10 fungal pathogens in molecular plant pathology. *Mol. Plant Pathol.* 13 414–430. 10.1111/j.1364-3703.2011.00783.x 22471698 PMC6638784

[B16] Desjardins A. E. (2003). Giberella from (Avenaceae) to (Zeae). *Annu. Rev. Phytopathol.* 41 177–198. 10.1146/annurev.phyto.41.011703.115501 12651961

[B17] Dill-Macky R. (2008). Cultural control practices for Fusarium head blight: problems and solutions. *Cereal Res. Commun.* 36 653–657. 10.1556/CRC.36.2008.Suppl.B.55

[B18] Dill-Macky R. Jones R. K. (2000). The effect of previous crop residues and tillage on Fusarium head blight of wheat. *Plant Dis.* 84 71–76. 10.1094/PDIS.2000.84.1.71 30841225

[B19] Dodt M. Roehr J. T. Ahmed R. Dieterich C. (2012). FLEXBAR- Flexible barcode and adapter processing for next-generation sequencing platforms. *Biology* 1 895–905. 10.3390/biology1030895 24832523 PMC4009805

[B20] Dorn B. Forrer H. R. Jenny E. Wettstein F. E. Bucheli T. D. Vogelgsang S. (2011). *Fusarium* species complex and mycotoxins in grain maize from maize hybrid trials and from grower’s fields. *J. Appl. Microbiol.* 111 693–706. 10.1111/j.1365-2672.2011.05091.x 21714835

[B21] Dorn B. Forrer H. R. Schürch S. Vogelgsang S. (2009). *Fusarium* species complex on maize in Switzerland: occurrence, prevalence, impact and mycotoxins in commercial hybrids under natural infection. *Eur. J. Plant Pathol.* 125 51–61. 10.1007/s10658-009-9457-8

[B22] Edgar R. C. (2010). Search and clustering orders of magnitude faster than BLAST. *Bioinformatics* 26 2460–2461. 10.1093/bioinformatics/btq461 20709691

[B23] Edgar R. C. Haas B. J. Clemente J. C. Quince C. Knight R. (2011). UCHIME improves sensitivity and speed of chimera detection. *Bioinformatics* 27 2194–2200. 10.1093/bioinformatics/btr381 21700674 PMC3150044

[B24] Edwards S. G. (2004). Influence of agricultural practices on *Fusarium* infection of cereals and subsequent contamination of grain by trichothecene mycotoxins. *Toxicol. Lett.* 153 29–35. 10.1016/j.toxlet.2004.04.022 15342078

[B25] Edwards S. G. Imathiu S. M. Ray R. V. Back M. Hare M. C. (2012). Molecular studies to identify the *Fusarium* species responsible for HT-2 and T-2 mycotoxins in UK oats. *Int. J. Food Microbiol.* 156 168–175. 10.1016/j.ijfoodmicro.2012.03.020 22521800

[B26] Eren A. M. Maignien L. Sul W. J. Murphy L. G. Grim S. L. Morrison H. G. (2013). Oligotyping: differentiating between closely related microbial taxa using 16S rRNA gene data. *Methods Ecol. Evol.* 4 1111–1119. 10.1111/2041-210X.12114 24358444 PMC3864673

[B27] Fernandez M. R. Ulrich D. Sproule L. Brandt S. A. Thomas A. G. Olfert O. (2007). “Impact of crop management systems on diseases of spring wheat on the Canadian Prairies,” in *Wheat Production in Stressed Environments*, eds Buck H. T. Nisi J. E. Salomon N. (Berlin: Springer), 265–271.

[B28] Franzén O. Hu J. Bao X. Itzkowitz S. H. Peter I. Bashir A. (2015). Improved OTU-picking using long-read 16S rRNA gene amplicon sequencing and generic hierarchical clustering. *Microbiome* 3:43. 10.1186/s40168-015-0105-6 26434730 PMC4593230

[B29] Friberg H. Edel-Hermann V. Faivre C. Gautheron N. Fayolle L. Faloya V. (2009). Cause and duration of mustard incorporation effects on soil-borne plant pathogenic fungi. *Soil Biol. Biochem.* 41 2075–2084. 10.1016/j.soilbio.2009.07.017

[B30] Gao X. Jackson T. A. Lambert K. N. Li S. Hartman G. L. (2004). Detection and quantification of *Fusarium solani* f. sp. *glycines* in soybean roots with real-time quantitative polymerase chain reaction. *Plant Dis.* 88 1372–1380. 10.1094/PDIS.2004.88.12.1372 30795200

[B31] Glushakova A. M. Chernov I. Y. (2010). Seasonal dynamics of the structure of epiphytic yeast communities. *Microbiology* 79 830–839. 10.1134/S0026261710060160 21774169

[B32] Guadet J. Julien J. Lafay J. F. Brygoo Y. (1989). Phylogeny of some *Fusarium* species, as determined by large-subunit rRNA sequence comparison. *Mol. Biol. Evol.* 6 227–242. 2622333 10.1093/oxfordjournals.molbev.a040548

[B33] Hennequin C. Abachin E. Symoens F. Lavarde V. Reboux G. Nolard N. (1999). Identification of *Fusarium* species involved in human infections by 28S rRNA gene sequencing. *J. Clin. Microbiol.* 37 3586–3589. 10523557 10.1128/jcm.37.11.3586-3589.1999 PMC85699

[B34] Hogg A. C. Johnston R. H. Dyer A. T. (2007). Applying real-time quantitative PCR to Fusarium crown rot of wheat. *Plant Dis.* 91 1021–1028. 10.1094/PDIS-91-8-1021 30780437

[B35] Infantino A. Santori A. Shah D. A. (2012). Community structure of the *Fusarium* complex on wheat seed in Italy. *Eur. J. Plant Pathol.* 132 499–510. 10.1007/s10658-011-9892-1

[B36] Jenkinson P. Parry D. W. (1994). Isolation of *Fusarium* species from common broad-leaved weeds their pathogenicity to winter wheat. *Mycol. Res.* 98 776–780. 10.1016/S0953-7562(09)81054-X

[B37] Jennings P. Coates M. E. Walsh K. Turner J. A. Nicholson P. (2004). Determination of deoxynivalenol-and nivalenol-producing chemotypes of *Fusarium graminearum* isolated from wheat crops in England and Wales. *Plant Pathol.* 53 643–652. 10.1111/j.0032-0862.2004.01061.x

[B38] Karlsson I. Edel-Hermann V. Gautheron N. Durling M. B. Kolseth A. K. Steinberg C. (2016). Genus-specific primers for study of *Fusarium* communities in field samples. *Appl. Environ. Microbiol.* 82 491–501. 10.1128/AEM.02748-15 26519387 PMC4711133

[B39] Karlsson I. Friberg H. Kolseth A.-K. Steinberg C. Persson P. (2017). Agricultural factors affecting *Fusarium* communities in wheat kernels. *Int. J. Food Microbiol.* 252 53–60. 10.1016/j.ijfoodmicro.2017.04.011 28463719

[B40] Kembel S. W. Mueller R. C. (2014). Plant traits and taxonomy drive host associations in tropical phyllosphere fungal communities. *Botany* 92 303–311. 10.1139/cjb-2013-0194

[B41] King J. E. Cook R. J. Melville S. C. (1983). A review of *Septoria* diseases of wheat and barley. *Ann. Appl. Biol.* 103 345–373. 10.1111/j.1744-7348.1983.tb02773.x

[B42] Kõljalg U. Nilsson R. H. Abarenkov K. Tedersoo L. Taylor A. F. S. Bahram M. (2013). Towards a unified paradigm for sequence-based identification of fungi. *Mol. Ecol.* 22 5271–5277. 10.1111/mec.12481 24112409

[B43] Lager J. Wallenhammar A.-C. (2003). Crop loss from soil-borne pathogens in white clover stands assessed by chemical treatments. *J. Plant Dis. Prot.* 110 120–128.

[B44] Lauber C. L. Ramirez K. S. Aanderud Z. Lennon J. Fierer N. (2013). Temporal variability in soil microbial communities across land-use types. *ISME J.* 7 1641–1650. 10.1038/ismej.2013.50 23552625 PMC3721119

[B45] Leff J. W. Fierer N. (2013). Bacterial communities associated with the surfaces of fresh fruits and vegetables. *PLOS ONE* 8:e59310. 10.1371/journal.pone.0059310 23544058 PMC3609859

[B46] Leslie J. Bowden R. (2008). *Fusarium graminearum*: when species concepts collide. *Cereal Res. Commun.* 36 609–615. 10.1556/CRC.36.2008.Suppl.B.50

[B47] Leslie J. F. Anderson L. L. Bowden R. L. Lee Y. W. (2007). Inter- and intra-specific genetic variation in *Fusarium*. *Int. J. Food Microbiol.* 119 25–32. 10.1016/j.ijfoodmicro.2007.07.059 17854936

[B48] Leslie J. F. Summerell B. A. (2008). *The Fusarium Laboratory Manual.* Hoboken, NJ: John Wiley & Sons.

[B49] Lindahl B. D. Nilsson R. H. Tedersoo L. Abarenkov K. Carlsen T. Kjøller R. (2013). Fungal community analysis by high-throughput sequencing of amplified markers-a user’s guide. *New Phytol.* 199 288–299. 10.1111/nph.12243 23534863 PMC3712477

[B50] Lukow T. Dunfield P. Liesack W. (2000). Use of the T-RFLP technique to assess spatial and temporal changes in the bacterial community structure within an agricultural soil planted with transgenic. *FEMS Microbiol. Ecol.* 32 241–247. 10.1111/j.1574-6941.2000.tb00717.x/full 10858583

[B51] Ma L.-J. van der Does H. C. Borkovich K. A. Coleman J. J. Daboussi M.-J. Di Pietro A. (2010). Comparative genomics reveals mobile pathogenicity chromosomes in *Fusarium*. *Nature* 464 367–373. 10.1038/nature08850 20237561 PMC3048781

[B52] Marín P. Moretti A. Ritieni A. Jurado M. Vázquez C. González-Jaén M. T. (2012). Phylogenetic analyses and toxigenic profiles of *Fusarium equiseti* and *Fusarium acuminatum* isolated from cereals from Southern Europe. *Food Microbiol.* 31 229–237. 10.1016/j.fm.2012.03.014 22608228

[B53] McMurdie P. J. Holmes S. (2013). Phyloseq: an R package for reproducible interactive analysis and graphics of microbiome census data. *PLOS ONE* 8:e61217. 10.1371/journal.pone.0061217 23630581 PMC3632530

[B54] Montes M. J. Belloch C. Galiana M. Garcia M. D. Andrés C. Ferrer S. (1999). Polyphasic taxonomy of a novel yeast isolated from antarctic environment; description of *Cryptococcus victoriae* sp. nov. *Syst. Appl. Microbiol.* 22 97–105. 10.1016/S0723-2020(99)80032-0 10188283

[B55] Munkvold G. P. (2003). Epidemiology of *Fusarium* diseases and their mycotoxins in maize ears. *Eur. J. Plant Pathol.* 109 705–713. 10.1023/A:1026078324268

[B56] Nakase T. Suzuki M. (1987). Studies on ballistospore-forming dead leaves of *Miscanthus sinensis* of the new species *Sporobolomyces miscanthi*, *Sporobolomyces subroseus*, and *Sporobolomyces weijmani*. *J. Gen. Appl. Microbiol.* 33 177–196. 10.2323/jgam.33.177

[B57] Nelson P. E. Toussoun T. A. Marasas W. F. O. (1983). *Fusarium Species: An Illustrated Manual for Identification.* University Park, PA: Penn State University Press.

[B58] Nganje W. E. Bangsund D. A. Leistritz F. L. Wilson W. W. Tiapo N. M. (2004). Regional economic impacts of Fusarium head blight in wheat and barley. *Rev. Agric. Econ.* 26 332–347. 10.1111/j.1467-9353.2004.00183.x

[B59] Nilsson R. H. Tedersoo L. Abarenkov K. Carlsen T. Pennanen T. Stenlid J. (2013). Methods fungal community analysis by high-throughput sequencing of amplified markers – a user’s guide. *New Phytol.* 199 288–299. 10.1111/nph.12243 23534863 PMC3712477

[B60] O’Donnell K. O. Cigelnik E. (1997). Two divergent intragenomic rDNA ITS2 types within a monophyletic lineage of the fungus *Fusarium* are nonorthologous. *Mol. Phylogenet. Evol.* 7 103–116. 10.1006/mpev.1996.0376 9007025

[B61] O’Donnell K. O. Cigelnik E. Nirenberg H. I. (1998). Molecular systematics and phylogeography of the Gibberella fujikuroi species complex. *Mycologia* 90 465–493. 10.2307/3761407

[B62] O’Donnell K. O. Rooney A. P. Proctor R. H. Brown D. W. McCormick S. P. Ward T. J. (2013). Phylogenetic analyses of RPB1 and RPB2 support a middle Cretaceous origin for a clade comprising all agriculturally and medically important fusaria. *Fungal Genet. Biol.* 52 20–31. 10.1016/j.fgb.2012.12.004 23357352

[B63] O’Donnell K. O. Sutton D. A. Fothergill A. McCarthy D. Rinaldi M. G. Brandt M. E. (2008a). Molecular phylogenetic diversity, multilocus haplotype nomenclature, and in vitro antifungal resistance within the *Fusarium solani* species complex. *J. Clin. Microbiol.* 46 2477–2490. 10.1128/JCM.02371-07 18524963 PMC2519483

[B64] O’Donnell K. O. Sutton D. A. Rinaldi M. G. Gueidan C. Crous P. W. Geiser D. M. (2009). Novel multilocus sequence typing scheme reveals high genetic diversity of human pathogenic members of the *Fusarium incarnatum-F. equiseti* and *F. chlamydosporum* species complexes within the United States. *J. Clin. Microbiol.* 47 3851–3861. 10.1128/JCM.01616-09 19828752 PMC2786663

[B65] O’Donnell K. O. Ward T. J. Aberra D. Kistler H. C. Aoki T. Orwig N. (2008b). Multilocus genotyping and molecular phylogenetics resolve a novel head blight pathogen within the *Fusarium graminearum* species complex from Ethiopia. *Fungal Genet. Biol.* 45 1514–1522. 10.1016/j.fgb.2008.09.002 18824240

[B66] O’Donnell K. O. Ward T. J. Robert V. A. R. G. Crous P. W. Geiser D. M. Kang S. (2015). DNA sequence-based identification of *Fusarium*: current status and future directions. *Phytoparasitica* 43 583–595. 10.1007/s12600-015-0484-z

[B67] Parry D. W. Jenkinson P. McLeod L. (1995). Fusarium ear blight (scab) in small grain cereals - a review. *Plant Pathol.* 44 207–238. 10.1111/j.1365-3059.1995.tb02773.x

[B68] Pasquali M. Beyer M. Logrieco A. Audenaert K. Balmas V. Basler R. (2016). A European database of *Fusarium graminearum* and *F. culmorum* trichothecene genotypes. *Front. Microbiol.* 7:406. 10.3389/fmicb.2016.00406 27092107 PMC4821861

[B69] R Core Team (2016). *R: A Language and Environment for Statistical Computing.* Available at: https://www.r-project.org/

[B70] Rastogi G. Sbodio A. Tech J. J. Suslow T. V. Coaker G. L. Leveau J. H. J. (2012). Leaf microbiota in an agroecosystem: spatiotemporal variation in bacterial community composition on field-grown lettuce. *ISME J.* 6 1812–1822. 10.1038/ismej.2012.32 22534606 PMC3446804

[B71] Reddy K. Salleh B. Saad B. Abbas H. Abel C. Shier W. (2010). An overview of mycotoxin contamination in foods and its implications for human health. *Toxin Rev.* 29 3–26. 10.3109/15569541003598553

[B72] Redford A. J. Bowers R. M. Knight R. Linhart Y. Fierer N. (2010). The ecology of the phyllosphere: geographic and phylogenetic variability in the distribution of bacteria on tree leaves. *Environ. Microbiol.* 12 2885–2893. 10.1111/j.1462-2920.2010.02258.x 20545741 PMC3156554

[B73] Rodland K. D. Russell P. J. (1982). Regulation of ribosomal RNA cistron number in a strain of *Neurospora crassa* with a duplication of the nucleolus organizer region. *Biochim. Biophys. Acta* 697 162–169. 10.1016/0167-4781(82)90072-0 6213268

[B74] Sampaio J. P. Gadanho M. Bauer R. Weiß M. (2003). Taxonomic studies in the Microbotryomycetidae: *Leucosporidium golubevii* sp. nov., *Leucosporidiella* gen. nov. and the new orders Leucosporidiales and Sporidiobolales. *Mycol. Prog.* 2 53–68. 10.1007/s11557-006-0044-5

[B75] Santamaria M. Vicario S. Pappadà G. Scioscia G. Scazzocchio C. Saccone C. (2009). Towards barcode markers in Fungi: an intron map of Ascomycota mitochondria. *BMC Bioinformatics* 10(Suppl. 6):S15. 10.1186/1471-2105-10-S6-S15 19534740 PMC2697638

[B76] Schlaeppi K. Bender S. F. Mascher F. Russo G. Patrignani A. Camenzind T. (2016). High-resolution community profiling of arbuscular mycorrhizal fungi. *New Phytol.* 212 780–791. 10.1111/nph.14070 27381250

[B77] Schlaeppi K. Dombrowski N. Oter R. G. Ver Loren van Themaat E. Schulze-Lefert P. (2014). Quantitative divergence of the bacterial root microbiota in *Arabidopsis thaliana* relatives. *Proc. Natl. Acad. Sci. U.S.A.* 111 585–592. 10.1073/pnas.1321597111 24379374 PMC3896156

[B78] Schloss P. D. Jenior M. L. Koumpouras C. C. Westcott S. L. Highlander S. K. (2016). Sequencing 16S rRNA gene fragments using the PacBio SMRT DNA sequencing system. *PeerJ* 4:e1869. 10.7717/peerj.1869 27069806 PMC4824876

[B79] Schloss P. D. Westcott S. L. Ryabin T. Hall J. R. Hartmann M. Hollister E. B. (2009). Introducing mothur: open-source, platform-independent, community-supported software for describing and comparing microbial communities. *Appl. Environ. Microbiol.* 75 7537–7541. 10.1128/AEM.01541-09 19801464 PMC2786419

[B80] Schoch C. L. Seifert K. A. Huhndorf S. Robert V. Spouge J. L. Levesque C. A. (2012). Nuclear ribosomal internal transcribed spacer (ITS) region as a universal DNA barcode marker for Fungi. *Proc. Natl. Acad. Sci. U.S.A.* 109 6241–6246. 10.1073/pnas.1117018109 22454494 PMC3341068

[B81] Schöneberg T. Martin C. Wettstein F. E. Bucheli T. D. Mascher F. Bertossa M. (2016). *Fusarium* and mycotoxin spectra in Swiss barley are affected by various cropping techniques. *Food Addit. Contam. Part A Chem. Anal. Control. Expo. Risk Assess.* 33 1608–1619. 10.1080/19440049.2016.1219071 27491813 PMC5215223

[B82] Seifert K. A. (2009). Progress towards DNA barcoding of fungi. *Mol. Ecol. Resour.* 9 83–89. 10.1111/j.1755-0998.2009.02635.x 21564968

[B83] Singer E. Bushnell B. Coleman-Derr D. Bowman B. Bowers R. M. Levy A. (2016). High-resolution phylogenetic microbial community profiling. *ISME J.* 10 2020–2032. 10.1038/ismej.2015.249 26859772 PMC5029162

[B84] Solomon P. S. Lowe R. G. T. Tan K. Waters O. D. C. Oliver R. P. (2006). *Stagonospora nodorum*: cause of *Stagonospora nodorum* blotch of wheat. *Mol. Plant Pathol.* 7 147–156. 10.1111/j.1364-3703.2006.00326.x 20507435

[B85] Stenglein S. A. (2009). *Fusarium poae*: a pathogen that needs more attention. *J. Plant Pathol.* 91 25–36.

[B86] Stȩpień Ł Koczyk G. Waśkiewicz A. (2011). Genetic and phenotypic variation of *Fusarium proliferatum* isolates from different host species. *J. Appl. Genet.* 52 487–496. 10.1007/s13353-011-0059-8 21796391 PMC3189322

[B87] Summerell B. A. Laurence M. H. Liew E. C. Y. Leslie J. F. (2010). Biogeography and phylogeography of *Fusarium*: a review. *Fungal Divers* 44 3–13. 10.1007/s13225-010-0060-2

[B88] Summerell B. A. Leslie J. F. (2011). Fifty years of *Fusarium*: how could nine species have ever been enough? *Fungal Divers* 50 135–144. 10.1007/s13225-011-0132-y

[B89] Tamura K. Stecher G. Peterson D. Filipski A. Kumar S. (2013). MEGA6: molecular evolutionary genetics analysis version 6.0. *Mol. Biol. Evol.* 30 2725–2729. 10.1093/molbev/mst197 24132122 PMC3840312

[B90] Tedersoo L. Tooming-Klunderud A. Anslan S. (2017). PacBio metabarcoding of Fungi and other eukaryotes: errors, biases and perspectives. *New Phytol.* 10.1111/nph.14776 [Epub ahead of print]. 28906012

[B91] Uhlig S. Jestoi M. Knutsen A. K. Heier B. T. (2006). Multiple regression analysis as a tool for the identification of relations between semi-quantitative LC-MS data and cytotoxicity of extracts of the fungus *Fusarium avenaceum* (syn. *F. arthrosporioides*). *Toxicon* 48 567–579. 10.1016/j.toxicon.2006.07.007 16908037

[B92] Vogelgsang S. Hecker A. Musa T. Dorn B. Forrer H. R. (2011). On-farm experiments over 5 years in a grain maize/winter wheat rotation: effect of maize residue treatments on *Fusarium graminearum* infection and deoxynivalenol contamination in wheat. *Mycotoxin Res.* 27 81–96. 10.1007/s12550-010-0079-y 23605700

[B93] Vogelgsang S. Jenny E. Hecker A. Baenziger I. Forrer H.-R. (2009). Fusaria and mycotoxins in wheat-monitoring of harvest samples from growers’ fields. *Agrarforschung* 16 238–243.

[B94] Vorholt J. A. (2012). Microbial life in the phyllosphere. *Nat. Rev. Microbiol.* 10 828–840. 10.1038/nrmicro2910 23154261

[B95] Waalwijk C. Koning J. R. A. de Baayen R. P. Gams W. (1996). Discordant groupings of *Fusarium* spp. from sections Elegans, Liseola and Dlaminia based on Ribosomal ITS1 and ITS2 Sequences. *Mycologia* 88 361–368. 10.2307/3760877

[B96] Waalwijk C. Van Der Heide R. De Vries I. Van Der Lee T. Schoen C. Costrel-de Corainville G. (2004). Quantitative detection of *Fusarium* species in wheat using TaqMan. *Eur. J. Plant Pathol.* 110 481–494. 10.1023/B:EJPP.0000032387.52385.13

[B97] Watanabe M. (2013). Molecular phylogeny and identification of *Fusarium* species based on nucleotide sequences. *Mycotoxins* 63 133–142. 10.2520/myco.63.133

[B98] Wittwer R. Dorn B. Jossi W. Van Der Heijden M. G. A. (2017). Cover crops support ecological intensification of arable cropping systems. *Sci. Rep.* 7:41911. 10.1038/srep41911 28157197 PMC5291223

[B99] Xu X. Nicholson P. (2009). Community ecology of fungal pathogens causing wheat head blight. *Annu. Rev. Phytopathol.* 47 83–103. 10.1146/annurev-phyto-080508-081737 19385728

[B100] Xu X. M. Parry D. W. Nicholson P. Thomsett M. A. Simpson D. Edwards S. G. (2005). Predominance and association of pathogenic fungi causing Fusarium ear blightin wheat in four European countries. *Eur. J. Plant Pathol.* 112 143–154. 10.1007/s10658-005-2446-7

[B101] Xue C. Tada Y. Dong X. Heitman J. (2007). The human hungal pathogen *Cryptococcus* can complete its sexual cycle during a pathogenic association with plants. *Cell Host Microbe* 1 263–273. 10.1016/j.chom.2007.05.005 18005707

[B102] Yli-Mattila T. Mach R. L. Alekhina I. A. Bulat S. A. Koskinen S. Kullnig-Gradinger C. M. (2004). Phylogenetic relationship of *Fusarium langsethiae* to *Fusarium poae* and *Fusarium sporotrichioides* as inferred by IGS, ITS, β-tubulin sequences and UP-PCR hybridization analysis. *Int. J. Food Microbiol.* 95 267–285. 10.1016/j.ijfoodmicro.2003.12.006 15337592

[B103] Yli-Mattila T. Paavanen-Huhtala S. Bulat S. A. Alekhina I. A. Nirenberg H. I. (2002). Molecular, morphological and phylogenetic analysis of the *Fusarium avenaceum*/*F. arthrosporioides*/*F. tricinctum* species complex – a polyphasic approach. *Mycol. Res.* 106 655–669. 10.1017/S0953756202006020

