# Supplementary material for: Community Profiling of Fusarium in Combination with Other Plant-Associated Fungi in Different Crop Species Using SMRT Sequencing
Source: Front Plant Sci. 2017 Nov 28;8:2019. doi: 10.3389/fpls.2017.02019 (PMC5712420; doi:10.3389/fpls.2017.02019)
Supplement: Supplementary file 1 [file Data_Sheet_1.ZIP › SupplementaryData1/Mthd_Rscript.rtf]

## R script for data analysis as presented in Figures:# - Fig 2# - Fig S3# - Fig S5# # 2017-09-14# Florian Walder, florianwalder@gmx.ch#### Load tools and data needed for the anaylsis ####rm(list=ls())##load all neededlibrary("ggplot2") library("plyr")library("reshape")library("reshape2")library("grid")library("gridExtra")library("scales")library("vegan")library("picante")library("gridExtra")library("phyloseq")library("RVAideMemoire")library("indicspecies")library(sciplot)source("https://bioconductor.org/biocLite.R")biocLite()source("~/Documents/R/Functions/vennDia.R")source("~/Documents/R/Functions/ggrare.R")source("~/Documents/R/Functions/errorbar.R")### upload map filedesignfile <- "MapFile.txt"alldesign <- read.table( designfile, header=T, na.strings = "NA", blank.lines.skip = FALSE)dim(alldesign)head(alldesign)length(rownames(alldesign))### upload otu tabledatafile <- "otu_table.txt"alldat <- as.matrix(read.table( paste( inputFolder, datafile, sep = "" ), row.names=1, sep="\t", header=T, blank.lines.skip = FALSE))rownames(alldat)<-gsub("denovo","otu", rownames(alldat)) #change denovo to OTUdim(alldat)head(alldat)length(colnames(alldat))### Binning of IsolateRefSeqs to OTUssort(rownames(alldat))#OTU F.ave|triF.avetri <- alldat["F.avenaceum(0379)",] + alldat["F.avenaceum(0380)",] + alldat["F.tricinctum(07015)",] + alldat["F.tricinctum(05009)",] alldat <- rbind(alldat, F.avetri)alldat <- alldat[ rownames(alldat)[! rownames(alldat) %in% c("F.avenaceum(0380)", "F.avenaceum(0379)", "F.tricinctum(05009)", "F.tricinctum(07015)") ], ]#OTU F.crookF.crook <- alldat["F.crookwellense(11080)",] + alldat["F.crookwellense(9703)",] + alldat["F.crookwellense(8125)",]alldat <- rbind(alldat, F.crook)alldat <- alldat[ rownames(alldat)[! rownames(alldat) %in% c("F.crookwellense(11080)", "F.crookwellense(9703)", "F.crookwellense(8125)") ], ]#OTU F.culF.cul <- alldat["F.culmorum(9712)",]alldat <- rbind(alldat, F.cul)alldat <- alldat[ rownames(alldat)[! rownames(alldat) %in% c("F.culmorum(9712)") ], ]#OTU F.equi_1F.equi_1 <- alldat["F.equiseti(05005)",]alldat <- rbind(alldat, F.equi_1)alldat <- alldat[ rownames(alldat)[! rownames(alldat) %in% c("F.equiseti(05005)") ], ]#OTU F.equi_2F.equi_2 <- alldat["F.equiseti(10015)",] + alldat["F.equiseti(11034)",]alldat <- rbind(alldat, F.equi_2)alldat <- alldat[ rownames(alldat)[! rownames(alldat) %in% c("F.equiseti(10015)", "F.equiseti(11034)") ], ]#OTU F.gramF.gram <- alldat["F.graminearum(0410)",]alldat <- rbind(alldat, F.gram)alldat <- alldat[ rownames(alldat)[! rownames(alldat) %in% c("F.graminearum(0410)") ], ]#OTU F.langF.lang <- alldat["F.langsethiae(0420)",]alldat <- rbind(alldat, F.lang)alldat <- alldat[ rownames(alldat)[! rownames(alldat) %in% c("F.langsethiae(0420)") ], ]#OTU F.oxyF.oxy <- alldat["F.oxysporum(07040)",]alldat <- rbind(alldat, F.oxy)alldat <- alldat[ rownames(alldat)[! rownames(alldat) %in% c("F.oxysporum(07040)") ], ]#OTU F.poaeF.poae <- alldat["F.poae(0378)",] + alldat["F.poae(0338)",] + alldat["F.poae(07027)",]alldat <- rbind(alldat, F.poae)alldat <- alldat[ rownames(alldat)[! rownames(alldat) %in% c("F.poae(0378)", "F.poae(0338)", "F.poae(07027)") ], ]#OTU F.prolF.prol <- alldat["F.proliferatum(05010)",] + alldat["F.proliferatum(7046)",] alldat <- rbind(alldat, F.prol)alldat <- alldat[ rownames(alldat)[! rownames(alldat) %in% c("F.proliferatum(05010)", "F.proliferatum(7046)") ], ]#OTU F.sporoF.sporo <-alldat["F.sporotrichoides(7044)",]alldat <- rbind(alldat, F.sporo)alldat <- alldat[ rownames(alldat)[! rownames(alldat) %in% c("F.sporotrichoides(7044)") ], ]#OTU F.subF.sub <- alldat["F.subglutinans(07038)",] + alldat["F.subglutinans(7043)",]alldat <- rbind(alldat, F.sub)alldat <- alldat[ rownames(alldat)[! rownames(alldat) %in% c("F.subglutinans(07038)", "F.subglutinans(7043)") ], ]#OTU F.venF.ven <- alldat["F.venenatum(11020)",]alldat <- rbind(alldat, F.ven)alldat <- alldat[ rownames(alldat)[! rownames(alldat) %in% c("F.venenatum(11020)") ], ]#OTU F.vertF.vert <- alldat["F.verticillioides(05007)",]alldat <- rbind(alldat, F.vert)alldat <- alldat[ rownames(alldat)[! rownames(alldat) %in% c("F.verticillioides(05007)") ], ]#Check if all Refseqs are binned to Fusarium taxasort(rownames(alldat))### remove low abundant otus (<0.5% => ND=0) in each sample >> abunDatdim(alldat)abunDat <- alldatsampleSums<-colSums(abunDat)for (i in 1:ncol(abunDat)){  abunDat[,i]<-ifelse(abunDat[,i]>0.005*sampleSums[i],abunDat[,i],0)}dim(abunDat)### identifying overlap samplesvd <- venndiagram(x=colnames(abunDat), y=rownames(alldesign), unique = T,                   labels=c("sequence data", "design file"),                   type ="2")samples_overlap <- vd$q1vd# only in sequencing data (not forseen in sampling design)t <- vd$q2colSums(abunDat)[t]# only in design file (no sequences)vd$q3### subsetting data and design files for general analysissamples_for_analysis <- rownames(alldesign)length(samples_overlap)# defining TechRep samplessamples_TechReps <- c("mthdF54R25", "mthdF55R25", "mthdF47R26")colSums(abunDat[, samples_TechReps])  # sample mthdF55R25 with most reads, other 2 samples removed for general analysis# defining samples for general analysis, removal of not used TechReps  samples_for_analysis <- samples_overlap[! samples_overlap %in% c("mthdF54R25", "mthdF47R26")]length(samples_for_analysis)# defining samples that samples for analysis contain a minimum of 100 sequencessamples_for_analysis_min <- samples_for_analysis[samples_for_analysis %in% colnames(abunDat)[colSums(abunDat) > 200]]length(samples_for_analysis_min)# subsetting data file dat <- abunDat[, samples_for_analysis_min]dat <- dat[rownames(dat)[rowSums(dat) > 1], ]sum(colSums(dat)) ##total number of reads sort(colSums(dat), decr=T)dim(dat)# subsetting design filedesign <- alldesign[samples_for_analysis_min,]dim(design)### upload unite taxonomy  datafile <- "unite_tax_assignments_4R.txt"unite_tax <- read.table( paste(datafile,sep= ""), row.names=1, sep="\t", header=F, blank.lines.skip = FALSE)dim(unite_tax)colnames(unite_tax) <- c("kingdom", "phylum", "class", "order", "family", "genus", "species")unite_tax$kingdom <-gsub("k__","", unite_tax$kingdom )unite_tax$phylum <-gsub("p__","", unite_tax$phylum )unite_tax$class <-gsub("c__","", unite_tax$class )unite_tax$order <-gsub("o__","", unite_tax$order )unite_tax$family <-gsub("f__","", unite_tax$family )unite_tax$genus <-gsub("g__","", unite_tax$genus )unite_tax$species <-gsub("s__","", unite_tax$species )head(unite_tax)##Rename OTUs in Taxonomy filerownames(unite_tax)<-gsub("denovo","otu", rownames(unite_tax)) #change denovo to OTUhead(unite_tax)as.matrix(unite_tax)rownames(unite_tax)#OTU F.ave|triF.avetri <- unite_tax["F.avenaceum(0379)",]rownames(F.avetri) <- c("F.avetri")unite_tax <- rbind(unite_tax, F.avetri)unite_tax <- unite_tax[ rownames(unite_tax)[! rownames(unite_tax) %in% c("F.avenaceum(0380)", "F.avenaceum(0379)", "F.tricinctum(07015)", "F.tricinctum(05009)") ], ]#OTU F.crookF.crook <- unite_tax["F.crookwellense(11080)",]rownames(F.crook) <- c("F.crook")unite_tax <- rbind(unite_tax, F.crook)unite_tax <- unite_tax[ rownames(unite_tax)[! rownames(unite_tax) %in% c("F.crookwellense(11080)","F.crookwellense(8125)", "F.crookwellense(9703)") ], ]#OTU F.culF.cul <- unite_tax["F.culmorum(9712)",]rownames(F.cul) <- c("F.cul")unite_tax <- rbind(unite_tax, F.cul)unite_tax <- unite_tax[ rownames(unite_tax)[! rownames(unite_tax) %in% c("F.culmorum(9712)") ], ]#OTU F.equi_1F.equi_1 <- unite_tax["F.equiseti(05005)",]rownames(F.equi_1) <- c("F.equi_1")unite_tax <- rbind(unite_tax, F.equi_1)unite_tax <- unite_tax[ rownames(unite_tax)[! rownames(unite_tax) %in% c("F.equiseti(05005)") ], ]#OTU F.equi_2F.equi_2 <- unite_tax["F.equiseti(10015)",]rownames(F.equi_2) <- c("F.equi_2")unite_tax <- rbind(unite_tax, F.equi_2)unite_tax <- unite_tax[ rownames(unite_tax)[! rownames(unite_tax) %in% c("F.equiseti(10015)", "F.equiseti(11034)") ], ]#OTU F.gramF.gram <- unite_tax["F.graminearum(0410)",]rownames(F.gram) <- c("F.gram")unite_tax <- rbind(unite_tax, F.gram)unite_tax <- unite_tax[ rownames(unite_tax)[! rownames(unite_tax) %in% c("F.graminearum(0410)") ], ]#OTU F.langF.lang <- unite_tax["F.langsethiae(0420)",]rownames(F.lang) <- c("F.lang")unite_tax <- rbind(unite_tax, F.lang)unite_tax <- unite_tax[ rownames(unite_tax)[! rownames(unite_tax) %in% c("F.langsethiae(0420)") ], ]#OTU F.oxyF.oxy <- unite_tax["F.oxysporum(07040)",]rownames(F.oxy) <- c("F.oxy")unite_tax <- rbind(unite_tax, F.oxy)unite_tax <- unite_tax[ rownames(unite_tax)[! rownames(unite_tax) %in% c("F.oxysporum(07040)") ], ]#OTU F.poaeF.poae <- unite_tax["F.poae(0378)",]rownames(F.poae) <- c("F.poae")unite_tax <- rbind(unite_tax, F.poae)unite_tax <- unite_tax[ rownames(unite_tax)[! rownames(unite_tax) %in% c("F.poae(0378)", "F.poae(0338)", "F.poae(07027)") ], ]#OTU F.prolF.prol <- unite_tax["F.proliferatum(05010)",]rownames(F.prol) <- c("F.prol")unite_tax <- rbind(unite_tax, F.prol)unite_tax <- unite_tax[ rownames(unite_tax)[! rownames(unite_tax) %in% c("F.proliferatum(05010)", "F.proliferatum(7046)") ], ]#OTU F.sporoF.sporo <-unite_tax["F.sporotrichoides(7044)",]rownames(F.sporo) <- c("F.sporo")unite_tax <- rbind(unite_tax, F.sporo)unite_tax <- unite_tax[ rownames(unite_tax)[! rownames(unite_tax) %in% c("F.sporotrichoides(7044)") ], ]#OTU F.subF.sub <- unite_tax["F.subglutinans(07038)",]rownames(F.sub) <- c("F.sub")unite_tax <- rbind(unite_tax, F.sub)unite_tax <- unite_tax[ rownames(unite_tax)[! rownames(unite_tax) %in% c("F.subglutinans(07038)", "F.subglutinans(7043)") ], ]#OTU F.venF.ven <- unite_tax["F.venenatum(11020)",]rownames(F.ven) <- c("F.ven")unite_tax <- rbind(unite_tax, F.ven)unite_tax <- unite_tax[ rownames(unite_tax)[! rownames(unite_tax) %in% c("F.venenatum(11020)") ], ]#OTU F.vertF.vert <- unite_tax["F.verticillioides(05007)",]rownames(F.vert) <- c("F.vert")unite_tax <- rbind(unite_tax, F.vert)unite_tax <- unite_tax[ rownames(unite_tax)[! rownames(unite_tax) %in% c("F.verticillioides(05007)") ], ]##Check if all Refseqs are binned to Fusarium taxasort(rownames(unite_tax))##Check ifor Plant OTUs and get rid of them in the dat filedim(dat)fungal_dat_otus <- !rownames(dat) %in% rownames(subset(unite_tax, kingdom == "Plantae"))dat  <- dat[fungal_dat_otus, ]dim(dat)##Remove OTUs from taxonomy file not included in the analysisunite_tax <- unite_tax[rownames(dat), ]dim(unite_tax)rownames(unite_tax)unite_tax <- as.data.frame(unite_tax)#OTUs assigned to Fusarium/Gebberellasubset(unite_tax, genus == "Fusarium")subset(unite_tax, genus == "Gibberella")# setting the environmentdir.create( file.path(outputFolder, "RelAbundance") )RA_outputFolder <- paste(outputFolder, "RelAbundance/", sep="")### Defining Fusarium OTUsrownames(dat)Fusarium_OTUs <- c("F.avetri", "F.crook", "F.cul", "F.equi_1", "F.equi_2", "F.gram", "F.lang", "F.poae",  "F.prol", "F.sporo", "F.sub", "F.vert" ) length(Fusarium_OTUs)# normalization by sample size and transformation in "/100"dat_norm <- t(t(dat)/colSums(dat)) * 100 colSums(dat_norm)Colors <- rep("white", length(rownames(dat_norm)))names(Colors) <- rownames(dat_norm)rownames(dat_norm)Fusarium_OTUslength(Fusarium_OTUs)Fusarium_OTUs_in_dat <- Fusarium_OTUs[Fusarium_OTUs %in% rownames(dat_norm)]Colors[Fusarium_OTUs_in_dat] <- "coral"Colors##### Figure 2  ####dir.create( file.path(RA_outputFolder, "mock") )mock_outputFolder <- paste(RA_outputFolder, "mock/", sep="")## mock equalmockE<-dat_norm[,design$type=="mock[e]"]mockE_mean <- apply(mockE,1,mean)mockE_se <- apply(mockE,1,se)mockE_mean <- sort(mockE_mean[mockE_mean > 0],decr=T)mockE_meanmockE_se <- mockE_se[names(mockE_mean)]mockE_expected <- c(10.01, 10, 10, 10, 10, 10, 10, 10, 10, 9.99)rbind(mockE_mean, mockE_expected)mockEforR <- as.data.frame(t(rbind(mockE_mean, mockE_expected)))# BarplotmockE<-barplot(mockE_mean, col="white", ylim=c(0,50), ylab="Relative Abundance (%)",               cex.axis=1.25, cex.names=1.25, cex.lab=1.25, las=2)errorbar(mockE, mockE_mean, mockE_se, length=0.05, lwd=1)barplot(mockE_expected, col=alpha("grey60",0.25), border= "grey60",        ylim=c(0,50), axes = F, add = T)# Goodness of fitcor.test(mockEforR$mockE_mean, mockEforR$mockE_expected, method="spearman", exact=FALSE)## mock staggeredmockS<-dat_norm[,design$type=="mock[s]"]mockS_mean <- apply(mockS,1,mean)mockS_se <- apply(mockS,1,se)mockS_mean <- sort(mockS_mean[mockS_mean > 0],decr=T)mockS_meanmockS_se <- mockS_se[names(mockS_mean)]mockS_expected <- c(38.17, 38.17, 3.817, 0.3817, 3.817, 3.817, 3.817, 3.817, 3.817, 0.3817)rbind(mockS_mean, mockS_expected)mockSforR <- as.data.frame(t(rbind(mockS_mean, mockS_expected)))#BarplotmockS<-barplot(mockS_mean, col="white", ylim=c(0,50), ylab="Relative Abundance (%)",               cex.axis=1.25, cex.names=1.25, cex.lab=1.25, las=2)errorbar(mockS, mockS_mean, mockS_se, length=0.05, lwd=1)barplot(mockS_expected, col=alpha("grey60",0.25), border= "grey60", ylim=c(0,50), axes = F, add = T)# Goodness of fitcor.test(mockSforR$mockS_mean, mockSforR$mockS_expected, method="spearman", exact=FALSE)##### Figure 3 ###### Load data of seed health testsSHT <- read.table(SHT.txt, row.names=1, sep="\t", header=T, blank.lines.skip = FALSE)SHT <- as.matrix(SHT)dim(SHT)## Harmonization of taxa names sort(rownames(SHT))sort(rownames(dat_norm))# Add taxa unique to dat file to SHTFlang <- matrix(c(0, 0, 0, 0, 0, 0, 0, 0, 0, 0, 0, 0, 0, 0, 0, 0, 0, 0, 0, 0, 0, 0, 0, 0, 0), nrow=1, ncol=25)rownames(Flang) <- "F.lang"SHT <- rbind(SHT, Flang)otu103 <- matrix(c(0, 0, 0, 0, 0, 0, 0, 0, 0, 0, 0, 0, 0, 0, 0, 0, 0, 0, 0, 0, 0, 0, 0, 0, 0), nrow=1, ncol=25)rownames(otu103) <- "otu103"SHT <- rbind(SHT, otu103)otu143 <- matrix(c(0, 0, 0, 0, 0, 0, 0, 0, 0, 0, 0, 0, 0, 0, 0, 0, 0, 0, 0, 0, 0, 0, 0, 0, 0), nrow=1, ncol=25)rownames(otu143) <- "otu143"SHT <- rbind(SHT, otu143)otu148 <- matrix(c(0, 0, 0, 0, 0, 0, 0, 0, 0, 0, 0, 0, 0, 0, 0, 0, 0, 0, 0, 0, 0, 0, 0, 0, 0), nrow=1, ncol=25)rownames(otu148) <- "otu148"SHT <- rbind(SHT, otu148)otu165 <- matrix(c(0, 0, 0, 0, 0, 0, 0, 0, 0, 0, 0, 0, 0, 0, 0, 0, 0, 0, 0, 0, 0, 0, 0, 0, 0), nrow=1, ncol=25)rownames(otu165) <- "otu165"SHT <- rbind(SHT, otu165)otu199 <- matrix(c(0, 0, 0, 0, 0, 0, 0, 0, 0, 0, 0, 0, 0, 0, 0, 0, 0, 0, 0, 0, 0, 0, 0, 0, 0), nrow=1, ncol=25)rownames(otu199) <- "otu199"SHT <- rbind(SHT, otu199)otu206 <- matrix(c(0, 0, 0, 0, 0, 0, 0, 0, 0, 0, 0, 0, 0, 0, 0, 0, 0, 0, 0, 0, 0, 0, 0, 0, 0), nrow=1, ncol=25)rownames(otu206) <- "otu206"SHT <- rbind(SHT, otu206)otu22 <- matrix(c(0, 0, 0, 0, 0, 0, 0, 0, 0, 0, 0, 0, 0, 0, 0, 0, 0, 0, 0, 0, 0, 0, 0, 0, 0), nrow=1, ncol=25)rownames(otu22) <- "otu22"SHT <- rbind(SHT, otu22)otu222 <- matrix(c(0, 0, 0, 0, 0, 0, 0, 0, 0, 0, 0, 0, 0, 0, 0, 0, 0, 0, 0, 0, 0, 0, 0, 0, 0), nrow=1, ncol=25)rownames(otu222) <- "otu222"SHT <- rbind(SHT, otu222)otu244 <- matrix(c(0, 0, 0, 0, 0, 0, 0, 0, 0, 0, 0, 0, 0, 0, 0, 0, 0, 0, 0, 0, 0, 0, 0, 0, 0), nrow=1, ncol=25)rownames(otu244) <- "otu244"SHT <- rbind(SHT, otu244)otu254 <- matrix(c(0, 0, 0, 0, 0, 0, 0, 0, 0, 0, 0, 0, 0, 0, 0, 0, 0, 0, 0, 0, 0, 0, 0, 0, 0), nrow=1, ncol=25)rownames(otu254) <- "otu254"SHT <- rbind(SHT, otu254)otu256 <- matrix(c(0, 0, 0, 0, 0, 0, 0, 0, 0, 0, 0, 0, 0, 0, 0, 0, 0, 0, 0, 0, 0, 0, 0, 0, 0), nrow=1, ncol=25)rownames(otu256) <- "otu256"SHT <- rbind(SHT, otu256)otu273 <- matrix(c(0, 0, 0, 0, 0, 0, 0, 0, 0, 0, 0, 0, 0, 0, 0, 0, 0, 0, 0, 0, 0, 0, 0, 0, 0), nrow=1, ncol=25)rownames(otu273) <- "otu273"SHT <- rbind(SHT, otu273)otu276 <- matrix(c(0, 0, 0, 0, 0, 0, 0, 0, 0, 0, 0, 0, 0, 0, 0, 0, 0, 0, 0, 0, 0, 0, 0, 0, 0), nrow=1, ncol=25)rownames(otu276) <- "otu276"SHT <- rbind(SHT, otu276)otu277 <- matrix(c(0, 0, 0, 0, 0, 0, 0, 0, 0, 0, 0, 0, 0, 0, 0, 0, 0, 0, 0, 0, 0, 0, 0, 0, 0), nrow=1, ncol=25)rownames(otu277) <- "otu277"SHT <- rbind(SHT, otu277)otu282 <- matrix(c(0, 0, 0, 0, 0, 0, 0, 0, 0, 0, 0, 0, 0, 0, 0, 0, 0, 0, 0, 0, 0, 0, 0, 0, 0), nrow=1, ncol=25)rownames(otu282) <- "otu282"SHT <- rbind(SHT, otu282)otu285 <- matrix(c(0, 0, 0, 0, 0, 0, 0, 0, 0, 0, 0, 0, 0, 0, 0, 0, 0, 0, 0, 0, 0, 0, 0, 0, 0), nrow=1, ncol=25)rownames(otu285) <- "otu285"SHT <- rbind(SHT, otu285)otu288 <- matrix(c(0, 0, 0, 0, 0, 0, 0, 0, 0, 0, 0, 0, 0, 0, 0, 0, 0, 0, 0, 0, 0, 0, 0, 0, 0), nrow=1, ncol=25)rownames(otu288) <- "otu288"SHT <- rbind(SHT, otu288)otu298 <- matrix(c(0, 0, 0, 0, 0, 0, 0, 0, 0, 0, 0, 0, 0, 0, 0, 0, 0, 0, 0, 0, 0, 0, 0, 0, 0), nrow=1, ncol=25)rownames(otu298) <- "otu298"SHT <- rbind(SHT, otu298)otu30 <- matrix(c(0, 0, 0, 0, 0, 0, 0, 0, 0, 0, 0, 0, 0, 0, 0, 0, 0, 0, 0, 0, 0, 0, 0, 0, 0), nrow=1, ncol=25)rownames(otu30) <- "otu30"SHT <- rbind(SHT, otu30)otu340 <- matrix(c(0, 0, 0, 0, 0, 0, 0, 0, 0, 0, 0, 0, 0, 0, 0, 0, 0, 0, 0, 0, 0, 0, 0, 0, 0), nrow=1, ncol=25)rownames(otu340) <- "otu340"SHT <- rbind(SHT, otu340)otu351 <- matrix(c(0, 0, 0, 0, 0, 0, 0, 0, 0, 0, 0, 0, 0, 0, 0, 0, 0, 0, 0, 0, 0, 0, 0, 0, 0), nrow=1, ncol=25)rownames(otu351) <- "otu351"SHT <- rbind(SHT, otu351)otu359 <- matrix(c(0, 0, 0, 0, 0, 0, 0, 0, 0, 0, 0, 0, 0, 0, 0, 0, 0, 0, 0, 0, 0, 0, 0, 0, 0), nrow=1, ncol=25)rownames(otu359) <- "otu359"SHT <- rbind(SHT, otu359)otu359 <- matrix(c(0, 0, 0, 0, 0, 0, 0, 0, 0, 0, 0, 0, 0, 0, 0, 0, 0, 0, 0, 0, 0, 0, 0, 0, 0), nrow=1, ncol=25)rownames(otu359) <- "otu359"SHT <- rbind(SHT, otu359)otu367 <- matrix(c(0, 0, 0, 0, 0, 0, 0, 0, 0, 0, 0, 0, 0, 0, 0, 0, 0, 0, 0, 0, 0, 0, 0, 0, 0), nrow=1, ncol=25)rownames(otu367) <- "otu367"SHT <- rbind(SHT, otu367)otu37 <- matrix(c(0, 0, 0, 0, 0, 0, 0, 0, 0, 0, 0, 0, 0, 0, 0, 0, 0, 0, 0, 0, 0, 0, 0, 0, 0), nrow=1, ncol=25)rownames(otu37) <- "otu37"SHT <- rbind(SHT, otu37)otu412 <- matrix(c(0, 0, 0, 0, 0, 0, 0, 0, 0, 0, 0, 0, 0, 0, 0, 0, 0, 0, 0, 0, 0, 0, 0, 0, 0), nrow=1, ncol=25)rownames(otu412) <- "otu412"SHT <- rbind(SHT, otu412)otu421 <- matrix(c(0, 0, 0, 0, 0, 0, 0, 0, 0, 0, 0, 0, 0, 0, 0, 0, 0, 0, 0, 0, 0, 0, 0, 0, 0), nrow=1, ncol=25)rownames(otu421) <- "otu421"SHT <- rbind(SHT, otu421)otu44 <- matrix(c(0, 0, 0, 0, 0, 0, 0, 0, 0, 0, 0, 0, 0, 0, 0, 0, 0, 0, 0, 0, 0, 0, 0, 0, 0), nrow=1, ncol=25)rownames(otu44) <- "otu44"SHT <- rbind(SHT, otu44)otu47 <- matrix(c(0, 0, 0, 0, 0, 0, 0, 0, 0, 0, 0, 0, 0, 0, 0, 0, 0, 0, 0, 0, 0, 0, 0, 0, 0), nrow=1, ncol=25)rownames(otu47) <- "otu47"SHT <- rbind(SHT, otu47)otu476 <- matrix(c(0, 0, 0, 0, 0, 0, 0, 0, 0, 0, 0, 0, 0, 0, 0, 0, 0, 0, 0, 0, 0, 0, 0, 0, 0), nrow=1, ncol=25)rownames(otu476) <- "otu476"SHT <- rbind(SHT, otu476)otu533 <- matrix(c(0, 0, 0, 0, 0, 0, 0, 0, 0, 0, 0, 0, 0, 0, 0, 0, 0, 0, 0, 0, 0, 0, 0, 0, 0), nrow=1, ncol=25)rownames(otu533) <- "otu533"SHT <- rbind(SHT, otu533)otu54 <- matrix(c(0, 0, 0, 0, 0, 0, 0, 0, 0, 0, 0, 0, 0, 0, 0, 0, 0, 0, 0, 0, 0, 0, 0, 0, 0), nrow=1, ncol=25)rownames(otu54) <- "otu54"SHT <- rbind(SHT, otu54)otu557 <- matrix(c(0, 0, 0, 0, 0, 0, 0, 0, 0, 0, 0, 0, 0, 0, 0, 0, 0, 0, 0, 0, 0, 0, 0, 0, 0), nrow=1, ncol=25)rownames(otu557) <- "otu557"SHT <- rbind(SHT, otu557)otu57 <- matrix(c(0, 0, 0, 0, 0, 0, 0, 0, 0, 0, 0, 0, 0, 0, 0, 0, 0, 0, 0, 0, 0, 0, 0, 0, 0), nrow=1, ncol=25)rownames(otu57) <- "otu57"SHT <- rbind(SHT, otu57)otu572 <- matrix(c(0, 0, 0, 0, 0, 0, 0, 0, 0, 0, 0, 0, 0, 0, 0, 0, 0, 0, 0, 0, 0, 0, 0, 0, 0), nrow=1, ncol=25)rownames(otu572) <- "otu572"SHT <- rbind(SHT, otu572)otu588 <- matrix(c(0, 0, 0, 0, 0, 0, 0, 0, 0, 0, 0, 0, 0, 0, 0, 0, 0, 0, 0, 0, 0, 0, 0, 0, 0), nrow=1, ncol=25)rownames(otu588) <- "otu588"SHT <- rbind(SHT, otu588)otu592 <- matrix(c(0, 0, 0, 0, 0, 0, 0, 0, 0, 0, 0, 0, 0, 0, 0, 0, 0, 0, 0, 0, 0, 0, 0, 0, 0), nrow=1, ncol=25)rownames(otu592) <- "otu592"SHT <- rbind(SHT, otu592)otu596 <- matrix(c(0, 0, 0, 0, 0, 0, 0, 0, 0, 0, 0, 0, 0, 0, 0, 0, 0, 0, 0, 0, 0, 0, 0, 0, 0), nrow=1, ncol=25)rownames(otu596) <- "otu596"SHT <- rbind(SHT, otu596)otu627 <- matrix(c(0, 0, 0, 0, 0, 0, 0, 0, 0, 0, 0, 0, 0, 0, 0, 0, 0, 0, 0, 0, 0, 0, 0, 0, 0), nrow=1, ncol=25)rownames(otu627) <- "otu627"SHT <- rbind(SHT, otu627)otu665 <- matrix(c(0, 0, 0, 0, 0, 0, 0, 0, 0, 0, 0, 0, 0, 0, 0, 0, 0, 0, 0, 0, 0, 0, 0, 0, 0), nrow=1, ncol=25)rownames(otu665) <- "otu665"SHT <- rbind(SHT, otu665)otu666 <- matrix(c(0, 0, 0, 0, 0, 0, 0, 0, 0, 0, 0, 0, 0, 0, 0, 0, 0, 0, 0, 0, 0, 0, 0, 0, 0), nrow=1, ncol=25)rownames(otu666) <- "otu666"SHT <- rbind(SHT, otu666)otu67 <- matrix(c(0, 0, 0, 0, 0, 0, 0, 0, 0, 0, 0, 0, 0, 0, 0, 0, 0, 0, 0, 0, 0, 0, 0, 0, 0), nrow=1, ncol=25)rownames(otu67) <- "otu67"SHT <- rbind(SHT, otu67)otu84 <- matrix(c(0, 0, 0, 0, 0, 0, 0, 0, 0, 0, 0, 0, 0, 0, 0, 0, 0, 0, 0, 0, 0, 0, 0, 0, 0), nrow=1, ncol=25)rownames(otu84) <- "otu84"SHT <- rbind(SHT, otu84)otu98 <- matrix(c(0, 0, 0, 0, 0, 0, 0, 0, 0, 0, 0, 0, 0, 0, 0, 0, 0, 0, 0, 0, 0, 0, 0, 0, 0), nrow=1, ncol=25)rownames(otu98) <- "otu98"SHT <- rbind(SHT, otu98)SHT <- SHT[order(rownames(SHT)), ]dim(SHT)head(SHT)length(colnames(SHT))## Add taxa unique to SHT file to dat filedat_norm_comp <- dat_normF.equi <- dat_norm_comp["F.equi_1",] + dat_norm_comp["F.equi_2", ]dat_norm_comp <- dat_norm_comp[ rownames(dat_norm_comp)[! rownames(dat_norm_comp) %in% c("F.equi_1", "F.equi_2") ], ]dat_norm_comp <- rbind(dat_norm_comp, F.equi)F.ave <- dat_norm_comp["F.avetri",]dat_norm_comp <- rbind(dat_norm_comp, F.ave)dat_norm_comp <- dat_norm_comp[ rownames(dat_norm_comp)[! rownames(dat_norm_comp) %in% c("F.avetri") ], ]F_sol <- matrix(c(0, 0, 0, 0, 0, 0, 0, 0, 0, 0, 0, 0, 0, 0, 0, 0, 0, 0, 0, 0, 0, 0, 0, 0, 0, 0, 0, 0, 0), nrow=1, ncol=29)rownames(F_sol) <- "F.sol"dat_norm_comp <- rbind(dat_norm_comp, F_sol)F_oxy <- matrix(c(0, 0, 0, 0, 0, 0, 0, 0, 0, 0, 0, 0, 0, 0, 0, 0, 0, 0, 0, 0, 0, 0, 0, 0, 0, 0, 0, 0, 0), nrow=1, ncol=29)rownames(F_oxy) <- "F.oxy"dat_norm_comp <- rbind(dat_norm_comp, F_oxy)sort(rownames(SHT))sort(rownames(dat_norm_comp))## Figure 3a/bFusarium_OTUs_in_Comp <- c("F.ave",   "F.crook", "F.cul",   "F.equi",  "F.gram",  "F.lang",  "F.oxy",   "F.poae",  "F.prol",  "F.sol",   "F.sporo", "F.sub",   "F.vert" )Colors_Comp <- rep("white", length(rownames(dat_norm_comp)))names(Colors_Comp) <- rownames(dat_norm_comp)rownames(dat_norm_comp)Colors_Comp[Fusarium_OTUs_in_Comp] <- "grey60"Colors_CompBorder_Comp <- rep("grey60", length(rownames(dat_norm_comp)))names(Border_Comp) <- rownames(dat_norm_comp)rownames(dat_norm_comp)Border_Comp[Fusarium_OTUs_in_Comp] <- "black"Border_Comp ### upload inoculation experiment design fileFu_designfile <- "MapFile_Fu.txt"Fu_design <- read.table( Fu_designfile, header=T, na.strings = "NA", blank.lines.skip = FALSE)dim(Fu_design)head(Fu_design)### identifying overlap samplesvd <- venndiagram(x=colnames(SHT), y=rownames(Fu_design), unique = T,                   labels=c("sequence data", "design file"),                   type ="2")samples_overlap <- vd$q1vd# only in sequencing data (not forseen in sampling design)t <- vd$q2colSums(alldat)[t]# only in design file (no sequences)vd$q3# subsetting data file SHT <- SHT[, samples_overlap]dim(SHT)# subsetting design fileFu_Inc_design <- Fu_design[samples_overlap,]dim(Fu_Inc_design)# control treatmentctrl_RA<-dat_norm_comp[,design$type=="ctrl"]ctrl_RA_mean <- apply(ctrl_RA,1,mean)ctrl_RA_se <- apply(ctrl_RA,1,se)ctrl_RA_mean <- sort(ctrl_RA_mean[ctrl_RA_mean > 0],decr=T)ctrl_RA_meanctrl_RA_se <- ctrl_RA_se[names(ctrl_RA_mean)]ctrl_Inc <-SHT[ , Fu_Inc_design$sample=="ctrl"]ctrl_Inc_mean <- apply(ctrl_Inc,1,mean)ctrl_Inc_se <- apply(ctrl_Inc,1,se)ctrl_Inc_meanctrl_Inc_mean <- c(ctrl_Inc_mean*-1)ctrl_Inc_mean <- ctrl_Inc_mean[names(ctrl_RA_mean)]ctrl_Inc_se <- ctrl_Inc_se[names(ctrl_Inc_mean)]# Plot Figure 3apar(mfrow=c(1,1))ctrl_RA<-barplot(ctrl_RA_mean, col=Colors_Comp[names(ctrl_RA_mean)], border= Border_Comp[names(ctrl_RA_mean)],                 horiz=TRUE, xlim=c(-50, 75), ylim=c(0,17),                 cex.axis=0.8, cex.names=0.8, las=2, xaxt="n", yaxt="n")arrows(ctrl_RA_mean - ctrl_RA_se, ctrl_RA, ctrl_RA_mean + ctrl_RA_se, ctrl_RA, code = 3, angle = 90, length = 0.05)ctrl_Inc<-barplot(ctrl_Inc_mean,  col=Colors_Comp[names(ctrl_Inc_mean)], density = 30, horiz=TRUE,        cex.axis=1.2, cex.names=1.2, las=2, xaxt="n", add=T)arrows(ctrl_Inc_mean - ctrl_Inc_se, ctrl_Inc, ctrl_Inc_mean + ctrl_Inc_se, ctrl_Inc, code = 3, angle = 90, length = 0.05)axis(1,at=c(-100, -75, -50, -25, 0, 25, 50, 75, 100),labels=c(100, 75, 50, 25, 0, 25, 50, 75, 100), cex.axis=1.25)mtext(c("Incidence [%]", "Rel. Abundance [%]"), side=1, line=3, cex=1.25, at=c(-25, 37.5))dev.off()# inoculation treatmentinoc_RA<-dat_norm_comp[,design$type=="inoc"]inoc_RA_mean <- apply(inoc_RA,1,mean)inoc_RA_se <- apply(inoc_RA,1,se)inoc_RA_mean <- sort(inoc_RA_mean[inoc_RA_mean > 0],decr=T)inoc_RA_meaninoc_RA_se <- inoc_RA_se[names(inoc_RA_mean)]inoc_Inc <-SHT[ , Fu_Inc_design$sample=="inoc"]inoc_Inc_mean <- apply(inoc_Inc,1,mean)inoc_Inc_se <- apply(inoc_Inc,1,se)inoc_Inc_meaninoc_Inc_mean <- c(inoc_Inc_mean*-1)inoc_Inc_mean <- inoc_Inc_mean[names(inoc_RA_mean)]inoc_Inc_se <- inoc_Inc_se[names(inoc_Inc_mean)]# Plot Figure 3bpar(mfrow=c(1,1))inoc_RA<-barplot(inoc_RA_mean, col=Colors_Comp[names(inoc_RA_mean)], border= Border_Comp[names(inoc_RA_mean)], horiz=TRUE,                 xlim=c(-50, 75), ylim=c(0,17),                 cex.axis=1.2, cex.names=1.2, las=2, xaxt="n", yaxt="n")arrows(inoc_RA_mean - inoc_RA_se, inoc_RA, inoc_RA_mean + inoc_RA_se, inoc_RA, code = 3, angle = 90, length = 0.05)inoc_Inc<-barplot(inoc_Inc_mean,  col=Colors_Comp[names(inoc_Inc_mean)], density=30, horiz=TRUE,                  cex.axis=1.2, cex.names=1.2, las=2, xaxt="n", add=T)arrows(inoc_Inc_mean - inoc_Inc_se, inoc_Inc, inoc_Inc_mean + inoc_Inc_se, inoc_Inc, code = 3, angle = 90, length = 0.05)axis(1,at=c(-100, -75, -50, -25, 0, 25, 50, 75, 100),labels=c(100, 75, 50, 25, 0, 25, 50, 75, 100), cex.axis=1.25)mtext(c("Incidence [%]", "Rel. Abundance [%]"), side=1, line=3, cex=1.25, at=c(-25, 37.5))# Gather all the rho values of the inoculation field experimentInocRhoValues <- matrix(nrow=0, ncol=3)RA.temp <- Fusarium_dat_norm[ , design$sample=="ctrl1"]In.temp <- SHT[ , "ctrl1"]R <- format(cor.test(RA.temp, In.temp, method="spearman", exact=FALSE)$estimate, digits=2)p <- format(cor.test(RA.temp, In.temp, method="spearman", exact=FALSE)$p.value, digits=3)treat <- c("ctrl")ctrl1 <- cbind(R,p)ctrl1 <- cbind(ctrl1, treat)rownames(ctrl1) <- c("ctrl1")InocRhoValues <- rbind(InocRhoValues, ctrl1)RA.temp <- Fusarium_dat_norm[ , design$sample=="ctrl2"]In.temp <- SHT[ , "ctrl2"]R <- format(cor.test(RA.temp, In.temp, method="spearman", exact=FALSE)$estimate, digits=2)p <- format(cor.test(RA.temp, In.temp, method="spearman", exact=FALSE)$p.value, digits=3)treat <- c("ctrl")ctrl2 <- cbind(R,p)ctrl2 <- cbind(ctrl2, treat)rownames(ctrl2) <- c("ctrl2")InocRhoValues <- rbind(InocRhoValues, ctrl2)RA.temp <- Fusarium_dat_norm[ , design$sample=="ctrl3"]In.temp <- SHT[ , "ctrl3"]R <- format(cor.test(RA.temp, In.temp, method="spearman", exact=FALSE)$estimate, digits=2)p <- format(cor.test(RA.temp, In.temp, method="spearman", exact=FALSE)$p.value, digits=3)treat <- c("ctrl")ctrl3 <- cbind(R,p)ctrl3 <- cbind(ctrl3, treat)rownames(ctrl3) <- c("ctrl3")InocRhoValues <- rbind(InocRhoValues, ctrl3)RA.temp <- Fusarium_dat_norm[ , design$sample=="ctrl4"]In.temp <- SHT[ , "ctrl4"]R <- format(cor.test(RA.temp, In.temp, method="spearman", exact=FALSE)$estimate, digits=2)p <- format(cor.test(RA.temp, In.temp, method="spearman", exact=FALSE)$p.value, digits=3)treat <- c("ctrl")ctrl4 <- cbind(R,p)ctrl4 <- cbind(ctrl4, treat)rownames(ctrl4) <- c("ctrl4")InocRhoValues <- rbind(InocRhoValues, ctrl4)RA.temp <- Fusarium_dat_norm[ , design$sample=="inoc1"]In.temp <- SHT[ , "inoc1"]R <- format(cor.test(RA.temp, In.temp, method="spearman", exact=FALSE)$estimate, digits=2)p <- format(cor.test(RA.temp, In.temp, method="spearman", exact=FALSE)$p.value, digits=3)treat <- c("inoc")inoc1 <- cbind(R,p)inoc1 <- cbind(inoc1, treat)rownames(inoc1) <- c("inoc1")InocRhoValues <- rbind(InocRhoValues, inoc1)RA.temp <- Fusarium_dat_norm[ , design$sample=="inoc2"]In.temp <- SHT[ , "inoc2"]R <- format(cor.test(RA.temp, In.temp, method="spearman", exact=FALSE)$estimate, digits=2)p <- format(cor.test(RA.temp, In.temp, method="spearman", exact=FALSE)$p.value, digits=3)treat <- c("inoc")inoc2 <- cbind(R,p)inoc2 <- cbind(inoc2, treat)rownames(inoc2) <- c("inoc2")InocRhoValues <- rbind(InocRhoValues, inoc2)RA.temp <- Fusarium_dat_norm[ , design$sample=="inoc3"]In.temp <- SHT[ , "inoc3"]R <- format(cor.test(RA.temp, In.temp, method="spearman", exact=FALSE)$estimate, digits=2)p <- format(cor.test(RA.temp, In.temp, method="spearman", exact=FALSE)$p.value, digits=3)treat <- c("inoc")inoc3 <- cbind(R,p)inoc3 <- cbind(inoc3, treat)rownames(inoc3) <- c("inoc3")InocRhoValues <- rbind(InocRhoValues, inoc3)RA.temp <- Fusarium_dat_norm[ , design$sample=="inoc4"]In.temp <- SHT[ , "inoc4"]R <- format(cor.test(RA.temp, In.temp, method="spearman", exact=FALSE)$estimate, digits=2)p <- format(cor.test(RA.temp, In.temp, method="spearman", exact=FALSE)$p.value, digits=3)treat <- c("inoc")inoc4 <- cbind(R,p)inoc4 <- cbind(inoc4, treat)rownames(inoc4) <- c("inoc4")InocRhoValues <- rbind(InocRhoValues, inoc4)df_InocRhoValues <- as.data.frame(InocRhoValues)df_InocRhoValues$R <- as.numeric(as.character(df_InocRhoValues$R))df_InocRhoValues$p <- as.numeric(as.character(df_InocRhoValues$p))tapply(df_InocRhoValues$R, list(df_InocRhoValues$treat), mean)tapply(df_InocRhoValues$R, list(df_InocRhoValues$treat), se)tapply(df_InocRhoValues$p, list(df_InocRhoValues$treat), mean)## Figure 3c: Screening Samples# Harmonization of taxa names Flang <- matrix(c(0, 0, 0, 0, 0, 0, 0, 0, 0, 0, 0, 0, 0, 0, 0, 0, 0, 0, 0, 0, 0, 0, 0, 0, 0), nrow=1, ncol=25)rownames(Flang) <- "F.lang"SHT <- rbind(SHT, Flang)SHT <- SHT[order(rownames(SHT)), ]dim(SHT)head(SHT)length(colnames(SHT))# Generate pure Fusarium RAFusarium_OTUs_in_dat_norm <- Fusarium_OTUs[Fusarium_OTUs %in% rownames(dat_norm)]Fusarium_dat_norm <- dat_norm[Fusarium_OTUs_in_dat_norm,]dim(Fusarium_dat_norm)# Harmonization of taxa names F.equi <- Fusarium_dat_norm["F.equi_1",] + Fusarium_dat_norm["F.equi_2", ]Fusarium_dat_norm <- Fusarium_dat_norm[ rownames(Fusarium_dat_norm)[! rownames(Fusarium_dat_norm) %in% c("F.equi_1", "F.equi_2") ], ]Fusarium_dat_norm <- rbind(Fusarium_dat_norm, F.equi)F.ave <- Fusarium_dat_norm["F.avetri",]Fusarium_dat_norm <- rbind(Fusarium_dat_norm, F.ave)Fusarium_dat_norm <- Fusarium_dat_norm[ rownames(Fusarium_dat_norm)[! rownames(Fusarium_dat_norm) %in% c("F.avetri") ], ]F_sol <- matrix(c(0, 0, 0, 0, 0, 0, 0, 0, 0, 0, 0, 0, 0, 0, 0, 0, 0, 0, 0, 0, 0, 0, 0, 0, 0, 0, 0, 0, 0), nrow=1, ncol=29)rownames(F_sol) <- "F.sol"Fusarium_dat_norm <- rbind(Fusarium_dat_norm, F_sol)F_oxy <- matrix(c(0, 0, 0, 0, 0, 0, 0, 0, 0, 0, 0, 0, 0, 0, 0, 0, 0, 0, 0, 0, 0, 0, 0, 0, 0, 0, 0, 0, 0), nrow=1, ncol=29)rownames(F_oxy) <- "F.oxy"Fusarium_dat_norm <- rbind(Fusarium_dat_norm, F_oxy)Fusarium_dat_norm <- Fusarium_dat_norm[order(rownames(Fusarium_dat_norm)), ]rownames(SHT)rownames(Fusarium_dat_norm)# Gather all the rho and p values of 15 screening samples# Mz1SumRhoValues <- matrix(nrow=0, ncol=3)RA.temp <- Fusarium_dat_norm[ , design$sample=="Mz1"]In.temp <- SHT[ , "Mz1"]R <- format(cor.test(RA.temp, In.temp, method="spearman", exact=FALSE)$estimate, digits=2)p <- format(cor.test(RA.temp, In.temp, method="spearman", exact=FALSE)$p.value, digits=3)crop <- c("maize")Mz1 <- cbind(R,p)Mz1 <- cbind(Mz1, crop)rownames(Mz1) <- c("Mz1")SumRhoValues <- rbind(SumRhoValues, Mz1)#Mz11RA.temp <- Fusarium_dat_norm[ , design$sample=="Mz11"]In.temp <- SHT[ , "Mz11"]R <- format(cor.test(RA.temp, In.temp, method="spearman", exact=FALSE)$estimate, digits=2)p <- format(cor.test(RA.temp, In.temp, method="spearman", exact=FALSE)$p.value, digits=3)crop <- c("maize")Mz11 <- cbind(R,p)Mz11 <- cbind(Mz11, crop)rownames(Mz11) <- c("Mz11")SumRhoValues <- rbind(SumRhoValues, Mz11)##Maize 18RA.temp <- Fusarium_dat_norm[ , design$sample=="Mz18"]In.temp <- SHT[ , "Mz18"]R <- format(cor.test(RA.temp, In.temp, method="spearman", exact=FALSE)$estimate, digits=2)p <- format(cor.test(RA.temp, In.temp, method="spearman", exact=FALSE)$p.value, digits=3)crop <- c("maize")Mz18 <- cbind(R,p)Mz18 <- cbind(Mz18, crop)rownames(Mz18) <- c("Mz18")SumRhoValues <- rbind(SumRhoValues, Mz18)##Maize 46RA.temp <- Fusarium_dat_norm[ , design$sample=="Mz46"]In.temp <- SHT[ , "Mz46"]R <- format(cor.test(RA.temp, In.temp, method="spearman", exact=FALSE)$estimate, digits=2)p <- format(cor.test(RA.temp, In.temp, method="spearman", exact=FALSE)$p.value, digits=3)crop <- c("maize")Mz46 <- cbind(R,p)Mz46 <- cbind(Mz46, crop)rownames(Mz46) <- c("Mz46")SumRhoValues <- rbind(SumRhoValues, Mz46)##Maize 56RA.temp <- Fusarium_dat_norm[ , design$sample=="Mz56"]In.temp <- SHT[ , "Mz56"]R <- format(cor.test(RA.temp, In.temp, method="spearman", exact=FALSE)$estimate, digits=2)p <- format(cor.test(RA.temp, In.temp, method="spearman", exact=FALSE)$p.value, digits=3)crop <- c("maize")Mz56 <- cbind(R,p)Mz56 <- cbind(Mz56, crop)rownames(Mz56) <- c("Mz56")SumRhoValues <- rbind(SumRhoValues, Mz56)##Barley 201RA.temp <- Fusarium_dat_norm[ , design$sample=="brl201"]In.temp <- SHT[ , "brl201"]R <- format(cor.test(RA.temp, In.temp, method="spearman", exact=FALSE)$estimate, digits=2)p <- format(cor.test(RA.temp, In.temp, method="spearman", exact=FALSE)$p.value, digits=3)crop <- c("barley")brl201 <- cbind(R,p)brl201 <- cbind(brl201, crop)rownames(brl201) <- c("brl201")SumRhoValues <- rbind(SumRhoValues, brl201)##Barley 88RA.temp <- Fusarium_dat_norm[ , design$sample=="brl88"]In.temp <- SHT[ , "brl88"]R <- format(cor.test(RA.temp, In.temp, method="spearman", exact=FALSE)$estimate, digits=2)p <- format(cor.test(RA.temp, In.temp, method="spearman", exact=FALSE)$p.value, digits=3)crop <- c("barley")brl88 <- cbind(R,p)brl88 <- cbind(brl88, crop)rownames(brl88) <- c("brl88")SumRhoValues <- rbind(SumRhoValues, brl88)##Barley 41RA.temp <- Fusarium_dat_norm[ , design$sample=="brl41"]In.temp <- SHT[ , "brl41"]R <- format(cor.test(RA.temp, In.temp, method="spearman", exact=FALSE)$estimate, digits=2)p <- format(cor.test(RA.temp, In.temp, method="spearman", exact=FALSE)$p.value, digits=3)crop <- c("barley")brl41 <- cbind(R,p)brl41 <- cbind(brl41, crop)rownames(brl41) <- c("brl41")SumRhoValues <- rbind(SumRhoValues, brl41)##Barley 27RA.temp <- Fusarium_dat_norm[ , design$sample=="brl27"]In.temp <- SHT[ , "brl27"]R <- format(cor.test(RA.temp, In.temp, method="spearman", exact=FALSE)$estimate, digits=2)p <- format(cor.test(RA.temp, In.temp, method="spearman", exact=FALSE)$p.value, digits=3)crop <- c("barley")brl27 <- cbind(R,p)brl27 <- cbind(brl27, crop)rownames(brl27) <- c("brl27")SumRhoValues <- rbind(SumRhoValues, brl27)##Barley 18RA.temp <- Fusarium_dat_norm[ , design$sample=="brl18"]In.temp <- SHT[ , "brl18"]R <- format(cor.test(RA.temp, In.temp, method="spearman", exact=FALSE)$estimate, digits=2)p <- format(cor.test(RA.temp, In.temp, method="spearman", exact=FALSE)$p.value, digits=3)crop <- c("barley")brl18 <- cbind(R,p)brl18 <- cbind(brl18, crop)rownames(brl18) <- c("brl18")SumRhoValues <- rbind(SumRhoValues, brl18)##Wheat 140RA.temp <- Fusarium_dat_norm[ , design$sample=="wht140"]In.temp <- SHT[ , "wht140"]R <- format(cor.test(RA.temp, In.temp, method="spearman", exact=FALSE)$estimate, digits=2)p <- format(cor.test(RA.temp, In.temp, method="spearman", exact=FALSE)$p.value, digits=3)crop <- c("wheat")wht140 <- cbind(R,p)wht140 <- cbind(wht140, crop)rownames(wht140) <- c("wht140")SumRhoValues <- rbind(SumRhoValues, wht140)##Wheat 103RA.temp <- Fusarium_dat_norm[ , design$sample=="wht103"]In.temp <- SHT[ , "wht103"]R <- format(cor.test(RA.temp, In.temp, method="spearman", exact=FALSE)$estimate, digits=2)p <- format(cor.test(RA.temp, In.temp, method="spearman", exact=FALSE)$p.value, digits=3)crop <- c("wheat")wht103 <- cbind(R,p)wht103 <- cbind(wht103, crop)rownames(wht103) <- c("wht103")SumRhoValues <- rbind(SumRhoValues, wht103)##Wheat 147RA.temp <- Fusarium_dat_norm[ , design$sample=="wht147"]In.temp <- SHT[ , "wht147"]R <- format(cor.test(RA.temp, In.temp, method="spearman", exact=FALSE)$estimate, digits=2)p <- format(cor.test(RA.temp, In.temp, method="spearman", exact=FALSE)$p.value, digits=3)crop <- c("wheat")wht147 <- cbind(R,p)wht147 <- cbind(wht147, crop)rownames(wht147) <- c("wht147")SumRhoValues <- rbind(SumRhoValues, wht147)##Wheat 187RA.temp <- Fusarium_dat_norm[ , design$sample=="wht187"]In.temp <- SHT[ , "wht187"]R <- format(cor.test(RA.temp, In.temp, method="spearman", exact=FALSE)$estimate, digits=2)p <- format(cor.test(RA.temp, In.temp, method="spearman", exact=FALSE)$p.value, digits=3)crop <- c("wheat")wht187 <- cbind(R,p)wht187 <- cbind(wht187, crop)rownames(wht187) <- c("wht187")SumRhoValues <- rbind(SumRhoValues, wht187)##Wheat 208RA.temp <- Fusarium_dat_norm[ , design$sample=="wht208"]In.temp <- SHT[ , "wht208"]R <- format(cor.test(RA.temp, In.temp, method="spearman", exact=FALSE)$estimate, digits=2)p <- format(cor.test(RA.temp, In.temp, method="spearman", exact=FALSE)$p.value, digits=3)crop <- c("wheat")wht208 <- cbind(R,p)wht208 <- cbind(wht208, crop)rownames(wht208) <- c("wht208")SumRhoValues <- rbind(SumRhoValues, wht208)df_SumRhoValues <- as.data.frame(SumRhoValues)df_SumRhoValues$R <- as.numeric(as.character(df_SumRhoValues$R))df_SumRhoValues$p <- as.numeric(as.character(df_SumRhoValues$p))Rho_mean <- tapply(df_SumRhoValues$R, list(df_SumRhoValues$crop), mean)Rho_sd <- tapply(df_SumRhoValues$R, list(df_SumRhoValues$crop), sd)sign <- c(2,2,1,2,2,1,2,2,2,2,2,2,2,2,2)df_SumRhoValues <- cbind(df_SumRhoValues,sign)df_SumRhoValues$sign <- as.factor(as.character(df_SumRhoValues$sign))screening <- c(rep("yes",15))df_SumRhoValues <- cbind(df_SumRhoValues,screening)Rho_wheat <- df_SumRhoValues[df_SumRhoValues$crop=="wheat",]Rho_wheat_ns <- Rho_wheat[Rho_wheat$sign=="1",]Rho_wheat_s <- Rho_wheat[Rho_wheat$sign=="2",]Rho_maize <- df_SumRhoValues[df_SumRhoValues$crop=="maize",]Rho_maize_ns <- Rho_maize[Rho_maize$sign=="1",]Rho_maize_s <- Rho_maize[Rho_maize$sign=="2",]Rho_barley <- df_SumRhoValues[df_SumRhoValues$crop=="barley",]Rho_barley_ns <- Rho_barley[Rho_barley$sign=="1",]Rho_barley_s <- Rho_barley[Rho_barley$sign=="2",]Rhos <- rbind(Rho_barley, Rho_maize)Rhos <- rbind(Rhos, Rho_wheat)tapply(Rhos$R, list(Rhos$screening), mean)tapply(Rhos$R, list(Rhos$screening), se)# Boxplot of rho valuesplot(R~screening, data = df_SumRhoValues, ylim=c(0,1), cex.axis=1.25, cex.lab=1.25, border="grey20",     ylab="Rho",  xlab = NULL, frame.plot = FALSE)stripchart(R~screening, data = Rho_wheat_ns, add = TRUE, cex= 1.5,           vertical = TRUE, method = "jitter", jitter=0.1,            pch = 0, col="palegreen4")stripchart(R~screening, data = Rho_wheat_s, add = TRUE, cex= 1.5,           vertical = TRUE, method = "jitter", jitter=0.1,           pch = 19, col="palegreen4")stripchart(R~screening, data = Rho_maize_ns, add = TRUE,cex= 1.5,           vertical = TRUE, method = "jitter", jitter=0.1,           pch = 1, col="gold3")stripchart(R~screening, data = Rho_maize_s, add = TRUE,cex= 1.5,           vertical = TRUE, method = "jitter", jitter=0.1,           pch = 19, col="gold3")stripchart(R~screening, data = Rho_barley_ns, add = TRUE,cex= 1.5,           vertical = TRUE, method = "jitter", jitter=0.1,           pch = 1, col="burlywood4")stripchart(R~screening, data = Rho_barley_s, add = TRUE,cex= 1.5,           vertical = TRUE, method = "jitter", jitter=0.1,           pch = 19, col="burlywood4")legend(0.5, 0.15, legend=c("wheat", "maize", "barley"),        bty="n", col=c("palegreen4", "gold3", "burlywood4"),       xpd=TRUE, pch=c(19), cex=1.25)##### Figure S1 ###### Figure 1Sa# export otu table to rare_output folderwrite.table(dat, "7_rarefy/otu_table_R.txt", sep="\t")# Rarefaction in Qiime### plotting OTU richnessobserved_OTUs <- "observed_species.txt"observed_OTUsdat <- read.table( paste( rare_inputFolder, "rare_abunDat_100-10000/alpha_collated/", observed_OTUs, sep = "" ), row.names=1, sep="\t", header=T, blank.lines.skip=FALSE, na.strings=c("n/a"))colnames(observed_OTUsdat)dim(observed_OTUsdat)#[3:43]observed_OTUsdat <- observed_OTUsdat[,c("sequences.per.sample", "iteration", colnames(observed_OTUsdat)[45000:34])]head(observed_OTUsdat)observed_OTUs_list <- split(observed_OTUsdat, observed_OTUsdat$sequences.per.sample)observed_OTUs_means <- lapply(observed_OTUs_list, colMeans)observed_OTUs_means_mat <- data.frame(observed_OTUs_means["100"])entries <- names(observed_OTUs_means)[! names(observed_OTUs_means) %in% "100"]for (i in entries){  x <- data.frame(observed_OTUs_means[names(observed_OTUs_means) == paste(i)])  observed_OTUs_means_mat <- cbind(observed_OTUs_means_mat, x)}colnames(observed_OTUs_means_mat) <- names(observed_OTUs_means)observed_OTUs_means_mat <- t(observed_OTUs_means_mat)dim(observed_OTUs_means_mat)head(observed_OTUs_means_mat)plot(x=observed_OTUs_means_mat[, 1], y=observed_OTUs_means_mat[,"mthdF52R27"], xlim=c(0, 2000), ylim=c(0,20), frame.plot=F, type="l",     lwd=1, cex.axis=1.25, cex.lab=1.25, col="grey20", ylab="OTUs observed", xlab="sequencing depth")lines(x=observed_OTUs_means_mat[,1], y=observed_OTUs_means_mat[,"mthdF47R27"], lwd=1, col="grey20")lines(x=observed_OTUs_means_mat[,1], y=observed_OTUs_means_mat[,"mthdF51R27"], lwd=1, col="grey20")lines(x=observed_OTUs_means_mat[,1], y=observed_OTUs_means_mat[,"mthdF52R25"], lwd=1, col="coral")lines(x=observed_OTUs_means_mat[,1], y=observed_OTUs_means_mat[,"mthdF53R26"], lwd=1, col="grey20")lines(x=observed_OTUs_means_mat[,1], y=observed_OTUs_means_mat[,"mthdF51R25"], lwd=1, col="coral")lines(x=observed_OTUs_means_mat[,1], y=observed_OTUs_means_mat[,"mthdF53R25"], lwd=1, col="coral")lines(x=observed_OTUs_means_mat[,1], y=observed_OTUs_means_mat[,"mthdF49R25"], lwd=1, col="coral")lines(x=observed_OTUs_means_mat[,1], y=observed_OTUs_means_mat[,"mthdF48R25"], lwd=1, col="coral")lines(x=observed_OTUs_means_mat[,1], y=observed_OTUs_means_mat[,"mthdF54R27"], lwd=1, col="grey20")lines(x=observed_OTUs_means_mat[,1], y=observed_OTUs_means_mat[,"mthdF54R28"], lwd=1, col="grey20")lines(x=observed_OTUs_means_mat[,1], y=observed_OTUs_means_mat[,"mthdF53R27"], lwd=1, col="grey20")lines(x=observed_OTUs_means_mat[,1], y=observed_OTUs_means_mat[,"mthdF47R25"], lwd=1, col="coral")lines(x=observed_OTUs_means_mat[,1], y=observed_OTUs_means_mat[,"mthdF55R25"], lwd=1, col="grey20")lines(x=observed_OTUs_means_mat[,1], y=observed_OTUs_means_mat[,"mthdF52R28"], lwd=1, col="grey20")lines(x=observed_OTUs_means_mat[,1], y=observed_OTUs_means_mat[,"mthdF51R25"], lwd=1, col="grey20")lines(x=observed_OTUs_means_mat[,1], y=observed_OTUs_means_mat[,"mthdF49R28"], lwd=1, col="grey20")lines(x=observed_OTUs_means_mat[,1], y=observed_OTUs_means_mat[,"mthdF48R27"], lwd=1, col="grey20")lines(x=observed_OTUs_means_mat[,1], y=observed_OTUs_means_mat[,"mthdF51R28"], lwd=1, col="grey20")### Figure S1b## Rarefaction analysis# plotting OTU richnessobserved_OTUs <- "observed_species.txt"observed_OTUsdat <- read.table( paste("rare_100-10000/alpha_collated/", observed_OTUs, sep = "" ), row.names=1, sep="\t", header=T, blank.lines.skip=FALSE, na.strings=c("n/a"))colnames(observed_OTUsdat)dim(observed_OTUsdat)observed_OTUsdat <- observed_OTUsdat[,c("sequences.per.sample", "iteration", colnames(observed_OTUsdat)[93000:34])]head(observed_OTUsdat)observed_OTUs_list <- split(observed_OTUsdat, observed_OTUsdat$sequences.per.sample)observed_OTUs_means <- lapply(observed_OTUs_list, colMeans)# binding mean values to dataframeobserved_OTUs_means_mat <- data.frame(observed_OTUs_means["100"])entries <- names(observed_OTUs_means)[! names(observed_OTUs_means) %in% "100"]for (i in entries){  x <- data.frame(observed_OTUs_means[names(observed_OTUs_means) == paste(i)])  observed_OTUs_means_mat <- cbind(observed_OTUs_means_mat, x)}colnames(observed_OTUs_means_mat) <- names(observed_OTUs_means)observed_OTUs_means_mat <- t(observed_OTUs_means_mat)dim(observed_OTUs_means_mat)head(observed_OTUs_means_mat)sort(colSums(alldat))plot(x=observed_OTUs_means_mat[, 1], y=observed_OTUs_means_mat[,"mthdF52R27"], xlim=c(0, 10000), ylim=c(0,200), frame.plot=F, type="l",     lwd=1, cex.axis=1.25, cex.lab=1.25, col="grey20", ylab="OTUs observed", xlab="sequencing depth")lines(x=observed_OTUs_means_mat[,1], y=observed_OTUs_means_mat[,"mthdF47R27"], lwd=1, col="grey20")lines(x=observed_OTUs_means_mat[,1], y=observed_OTUs_means_mat[,"mthdF51R27"], lwd=1, col="grey20")lines(x=observed_OTUs_means_mat[,1], y=observed_OTUs_means_mat[,"mthdF52R25"], lwd=1, col="coral")lines(x=observed_OTUs_means_mat[,1], y=observed_OTUs_means_mat[,"mthdF53R26"], lwd=1, col="grey20")lines(x=observed_OTUs_means_mat[,1], y=observed_OTUs_means_mat[,"mthdF51R25"], lwd=1, col="coral")lines(x=observed_OTUs_means_mat[,1], y=observed_OTUs_means_mat[,"mthdF53R25"], lwd=1, col="coral")lines(x=observed_OTUs_means_mat[,1], y=observed_OTUs_means_mat[,"mthdF49R25"], lwd=1, col="coral")lines(x=observed_OTUs_means_mat[,1], y=observed_OTUs_means_mat[,"mthdF48R25"], lwd=1, col="coral")lines(x=observed_OTUs_means_mat[,1], y=observed_OTUs_means_mat[,"mthdF54R27"], lwd=1, col="grey20")lines(x=observed_OTUs_means_mat[,1], y=observed_OTUs_means_mat[,"mthdF54R28"], lwd=1, col="grey20")lines(x=observed_OTUs_means_mat[,1], y=observed_OTUs_means_mat[,"mthdF53R27"], lwd=1, col="grey20")lines(x=observed_OTUs_means_mat[,1], y=observed_OTUs_means_mat[,"mthdF47R25"], lwd=1, col="coral")lines(x=observed_OTUs_means_mat[,1], y=observed_OTUs_means_mat[,"mthdF55R25"], lwd=1, col="grey20")lines(x=observed_OTUs_means_mat[,1], y=observed_OTUs_means_mat[,"mthdF52R28"], lwd=1, col="grey20")lines(x=observed_OTUs_means_mat[,1], y=observed_OTUs_means_mat[,"mthdF51R25"], lwd=1, col="grey20")lines(x=observed_OTUs_means_mat[,1], y=observed_OTUs_means_mat[,"mthdF49R28"], lwd=1, col="grey20")lines(x=observed_OTUs_means_mat[,1], y=observed_OTUs_means_mat[,"mthdF48R27"], lwd=1, col="grey20")lines(x=observed_OTUs_means_mat[,1], y=observed_OTUs_means_mat[,"mthdF51R28"], lwd=1, col="grey20")##### Figure S3 ##### normalization by sample size and transformation in "/100"dat_TR_norm <- t(t(dat_TR)/colSums(dat_TR)) * 100 colSums(dat_TR_norm)Cols <- rep("grey60", length(rownames(dat_TR_norm)))names(Cols) <- rownames(dat_TR_norm)rownames(dat_TR_norm)Fusarium_OTUs_in_TR <- Fusarium_OTUs[Fusarium_OTUs %in% rownames(dat_TR_norm)]Cols[Fusarium_OTUs_in_TR] <- "coral"Cols# Figure S3asort(apply(dat_TR_norm, 1, max), decr=F)pdf( paste(TR_outputFolder,"OTU_reproducibility_xyplot.pdf", sep=""), width=6, height=6)plot(x=dat_TR_norm[,"mthdF54R25"], y=dat_TR_norm[,"mthdF55R25"], pch=8, xlim=c(0.5,50), ylim=c(0.5,50), cex.axis=1.1, cex.lab =1.25,     bty="n",ylab="Relative Abundance [%]",xlab="Relative Abundance [%]", las=1, col=Cols, log="xy")points(x=dat_TR_norm[,"mthdF54R25"], y=dat_TR_norm[,"mthdF47R26"], pch=16, col=Cols)points(x=dat_TR_norm[,"mthdF55R25"], y=dat_TR_norm[,"mthdF47R26"], pch=17, col=Cols)combo16 <- cor(dat_TR_norm[,"mthdF54R25"], dat_TR_norm[,"mthdF55R25"], meth = "spearman")combo15 <- cor(dat_TR_norm[,"mthdF54R25"], dat_TR_norm[,"mthdF47R26"], meth = "spearman")combo56 <- cor(dat_TR_norm[,"mthdF55R25"], dat_TR_norm[,"mthdF47R26"], meth = "spearman")mean_spearman <- mean(c(combo16,combo15,combo56))mean_spearmanse(c(combo16,combo15,combo56))text(x=5, y=1.1, labels="Spearman coefficient:", adj=0.1,col="grey20", cex=1)text(x=5, y=0.85, labels=paste("barcode 1 vs. 2:  ", format(combo15, digits=3)), adj=0, col="grey20", cex=1)text(x=5, y=0.7, labels=paste("barcode 1 vs. 3:  ", format(combo16, digits=3)), adj=0, col="grey20", cex=1)text(x=5, y=0.575, labels=paste("barcode 2 vs. 3:  ", format(combo56, digits=3)), adj=0, col="grey20", cex=1)# Figure S3 bCols <- rep("white", length(rownames(dat_TR_norm)))names(Cols) <- rownames(dat_TR_norm)rownames(dat_TR_norm)Fusarium_OTUs_in_TR <- Fusarium_OTUs[Fusarium_OTUs %in% rownames(dat_TR_norm)]Cols[Fusarium_OTUs_in_TR] <- "coral"Colsalldat_TR_norm_means <- sort(apply(dat_TR_norm, 1, mean), decr=T)alldat_TR_norm_ses <- apply(dat_TR_norm, 1, se)[names(alldat_TR_norm_means)]Cols <- rep("white", length(rownames(dat_TR_norm)))names(Cols) <- rownames(dat_TR_norm)rownames(dat_TR_norm)length(Fusarium_OTUs)Fusarium_OTUs_in_TR <- Fusarium_OTUs[Fusarium_OTUs %in% rownames(dat_TR_norm)]Cols[Fusarium_OTUs_in_TR] <- "coral"Colsp <- barplot(alldat_TR_norm_means[1:20],              ylim=c(0,40),             col=Cols[names(alldat_TR_norm_means)],# ylim=c(0.5,50), log="y",             las=2,  cex.axis=1.25, cex.names=1.25, cex.lab=1.25, #main=" OTU abundance",              ylab="Relative Abundance [%]")errorbar(p, alldat_TR_norm_means[1:20], alldat_TR_norm_ses[1:20], length=0.05, lwd=1)
