# Supplementary material for: Community Profiling of Fusarium in Combination with Other Plant-Associated Fungi in Different Crop Species Using SMRT Sequencing
Source: Front Plant Sci. 2017 Nov 28;8:2019. doi: 10.3389/fpls.2017.02019 (PMC5712420; doi:10.3389/fpls.2017.02019)
Supplement: Supplementary file 2 [file Data_Sheet_2.ZIP › SupplementaryData2/OSC_Rscript.rtf]

## R script for data analysis as presented in Figures:# - Fig 5# - Fig 6# - Fig S5# # 2017-09-14# Florian Walder, florianwalder@gmx.ch# ## files needed: # - OSCAR_MapFile.txt# - OSCAR_otu_table.txt# - OSCAR_unite_tax_assignments_4R.txt# - OSCAR_RDP_tax_assignments_4R.txt##### Load tools and data used  ####rm(list=ls())library("ggplot2") library("ggrepel")library("plyr")library("reshape")library("reshape2")library("grid")library("gridExtra")library("scales")library("vegan")library("picante")library("gridExtra")library("phyloseq")library("RVAideMemoire")library("indicspecies")library(sciplot)library(PMCMR)library(Biostrings)library(gplots)library(RColorBrewer)source("https://bioconductor.org/biocLite.R")biocLite()source("~/Documents/R/Functions/vennDia.R")source("~/Documents/R/Functions/ggrare.R")source("~/Documents/R/Functions/errorbar.R")source("~/Documents/R/Functions/cpcoa_fun.R")source("~/Documents/R/Functions/plotOTU.R")source("~/Documents/R/Functions/maPalette.R")### upload map filedesignfile <- "OSCAR_MapFile.txt"alldesign <- read.table( designfile, header=T, na.strings = "NA", blank.lines.skip = FALSE)dim(alldesign)head(alldesign)length(rownames(alldesign))### upload otu tabledatafile <- "OSCAR_otu_table.txt"alldat <- as.matrix(read.table( paste(datafile, sep = "" ), row.names=1, sep="\t", header=T, blank.lines.skip = FALSE))rownames(alldat)<-gsub("denovo","otu", rownames(alldat)) #change denovo to OTUdim(alldat)head(alldat)length(colnames(alldat))### Binning of IsolateRefSeqs to OTUssort(rownames(alldat))#OTU F.ave|triF.avetri <- alldat["F.avenaceum(0379)",] + alldat["F.avenaceum(0380)",] + alldat["F.tricinctum(07015)",] + alldat["F.tricinctum(05009)",] alldat <- rbind(alldat, F.avetri)alldat <- alldat[ rownames(alldat)[! rownames(alldat) %in% c("F.avenaceum(0380)", "F.avenaceum(0379)", "F.tricinctum(05009)", "F.tricinctum(07015)") ], ]#OTU F.crookF.crook <- alldat["F.crookwellense(11080)",] + alldat["F.crookwellense(9703)",]# + alldat["F.crookwellense(8125)",]alldat <- rbind(alldat, F.crook)alldat <- alldat[ rownames(alldat)[! rownames(alldat) %in% c("F.crookwellense(11080)", "F.crookwellense(9703)") ], ]#OTU F.culF.cul <- alldat["F.culmorum(9712)",]alldat <- rbind(alldat, F.cul)alldat <- alldat[ rownames(alldat)[! rownames(alldat) %in% c("F.culmorum(9712)") ], ]#OTU F.equi_1F.equi_1 <- alldat["F.equiseti(05005)",]alldat <- rbind(alldat, F.equi_1)alldat <- alldat[ rownames(alldat)[! rownames(alldat) %in% c("F.equiseti(05005)") ], ]#OTU F.equi_2F.equi_2 <- alldat["F.equiseti(11034)",]alldat <- rbind(alldat, F.equi_2)alldat <- alldat[ rownames(alldat)[! rownames(alldat) %in% c("F.equiseti(11034)") ], ]#OTU F.gramF.gram <- alldat["F.graminearum(0410)",]alldat <- rbind(alldat, F.gram)alldat <- alldat[ rownames(alldat)[! rownames(alldat) %in% c("F.graminearum(0410)") ], ]#OTU F.langF.lang <- alldat["F.langsethiae(0420)",]alldat <- rbind(alldat, F.lang)alldat <- alldat[ rownames(alldat)[! rownames(alldat) %in% c("F.langsethiae(0420)") ], ]#OTU F.oxyF.oxy <- alldat["F.oxysporum(07040)",]alldat <- rbind(alldat, F.oxy)alldat <- alldat[ rownames(alldat)[! rownames(alldat) %in% c("F.oxysporum(07040)") ], ]#OTU F.poaeF.poae <- alldat["F.poae(0378)",] + alldat["F.poae(07027)",]alldat <- rbind(alldat, F.poae)alldat <- alldat[ rownames(alldat)[! rownames(alldat) %in% c("F.poae(0378)", "F.poae(07027)") ], ]#OTU F.prolF.prol <- alldat["F.proliferatum(05010)",] + alldat["F.proliferatum(7046)",] alldat <- rbind(alldat, F.prol)alldat <- alldat[ rownames(alldat)[! rownames(alldat) %in% c("F.proliferatum(05010)", "F.proliferatum(7046)") ], ]#OTU F.sporoF.sporo <-alldat["F.sporotrichoides(7044)",]alldat <- rbind(alldat, F.sporo)alldat <- alldat[ rownames(alldat)[! rownames(alldat) %in% c("F.sporotrichoides(7044)") ], ]#OTU F.subF.sub <- alldat["F.subglutinans(07038)",] + alldat["F.subglutinans(7043)",]alldat <- rbind(alldat, F.sub)alldat <- alldat[ rownames(alldat)[! rownames(alldat) %in% c("F.subglutinans(07038)", "F.subglutinans(7043)") ], ]#OTU F.venF.ven <- alldat["F.venenatum(11020)",]alldat <- rbind(alldat, F.ven)alldat <- alldat[ rownames(alldat)[! rownames(alldat) %in% c("F.venenatum(11020)") ], ]#OTU F.vertF.vert <- alldat["F.verticillioides(05007)",]alldat <- rbind(alldat, F.vert)alldat <- alldat[ rownames(alldat)[! rownames(alldat) %in% c("F.verticillioides(05007)") ], ]#Check if all Refseqs are binned to Fusarium taxasort(rownames(alldat))### remove low abundant otus (<0.5% => ND=0) in each sample >> abunDatdim(alldat)abunDat <- alldatsampleSums<-colSums(abunDat)for (i in 1:ncol(abunDat)){  abunDat[,i]<-ifelse(abunDat[,i]>0.005*sampleSums[i],abunDat[,i],0)}dim(abunDat)### identifying overlap samplesvd <- venndiagram(x=colnames(abunDat), y=rownames(alldesign), unique = T,                   labels=c("sequence data", "design file"),                   type ="2")samples_overlap <- vd$q1vd# only in sequencing data (not forseen in sampling design)t <- vd$q2colSums(abunDat)[t]# only in design file (no sequences)vd$q3### subsetting data and design files for general analysissamples_for_analysis <- rownames(alldesign)length(samples_overlap)# defining samples that samples for analysis contain a minimum of sequencessort(colSums(abunDat)) #480samples_for_analysis_min <- samples_for_analysis[samples_for_analysis %in% colnames(abunDat)[colSums(abunDat) > 480]]length(samples_for_analysis_min)# subsetting data file dat <- abunDat[, samples_for_analysis_min]dat <- dat[rownames(dat)[rowSums(dat) > 1], ]sum(colSums(dat)) ##total number of reads sort(colSums(dat), decr=T)dim(dat)# subsetting design filedesign <- alldesign[samples_for_analysis_min,]dim(design)## upload unite taxonomydatafile <- "OSCAR_unite_tax_assignments_4R.txt"unite_tax <- read.table( paste(datafile,sep= ""), row.names=1, sep="\t", header=F, blank.lines.skip = FALSE)dim(unite_tax)colnames(unite_tax) <- c("kingdom", "phylum", "class", "order", "family", "genus", "species")unite_tax$kingdom <-gsub("k__","", unite_tax$kingdom )unite_tax$phylum <-gsub("p__","", unite_tax$phylum )unite_tax$class <-gsub("c__","", unite_tax$class )unite_tax$order <-gsub("o__","", unite_tax$order )unite_tax$family <-gsub("f__","", unite_tax$family )unite_tax$genus <-gsub("g__","", unite_tax$genus )unite_tax$species <-gsub("s__","", unite_tax$species )head(unite_tax)##Rename OTUs in Taxonomy filerownames(unite_tax)<-gsub("denovo","otu", rownames(unite_tax)) #change denovo to OTUhead(unite_tax)as.matrix(unite_tax)rownames(unite_tax)#OTU F.ave|triF.avetri <- unite_tax["F.avenaceum(0379)",]rownames(F.avetri) <- c("F.avetri")unite_tax <- rbind(unite_tax, F.avetri)unite_tax <- unite_tax[ rownames(unite_tax)[! rownames(unite_tax) %in% c("F.avenaceum(0380)", "F.avenaceum(0379)", "F.tricinctum(07015)", "F.tricinctum(05009)") ], ]#OTU F.crookF.crook <- unite_tax["F.crookwellense(11080)",]rownames(F.crook) <- c("F.crook")unite_tax <- rbind(unite_tax, F.crook)unite_tax <- unite_tax[ rownames(unite_tax)[! rownames(unite_tax) %in% c("F.crookwellense(11080)","F.crookwellense(8125)", "F.crookwellense(9703)") ], ]#OTU F.culF.cul <- unite_tax["F.culmorum(9712)",]rownames(F.cul) <- c("F.cul")unite_tax <- rbind(unite_tax, F.cul)unite_tax <- unite_tax[ rownames(unite_tax)[! rownames(unite_tax) %in% c("F.culmorum(9712)") ], ]#OTU F.equi_1F.equi_1 <- unite_tax["F.equiseti(05005)",]rownames(F.equi_1) <- c("F.equi_1")unite_tax <- rbind(unite_tax, F.equi_1)unite_tax <- unite_tax[ rownames(unite_tax)[! rownames(unite_tax) %in% c("F.equiseti(05005)") ], ]#OTU F.equi_2F.equi_2 <- unite_tax["F.equiseti(10015)",]rownames(F.equi_2) <- c("F.equi_2")unite_tax <- rbind(unite_tax, F.equi_2)unite_tax <- unite_tax[ rownames(unite_tax)[! rownames(unite_tax) %in% c("F.equiseti(10015)", "F.equiseti(11034)") ], ]#OTU F.gramF.gram <- unite_tax["F.graminearum(0410)",]rownames(F.gram) <- c("F.gram")unite_tax <- rbind(unite_tax, F.gram)unite_tax <- unite_tax[ rownames(unite_tax)[! rownames(unite_tax) %in% c("F.graminearum(0410)") ], ]#OTU F.langF.lang <- unite_tax["F.langsethiae(0420)",]rownames(F.lang) <- c("F.lang")unite_tax <- rbind(unite_tax, F.lang)unite_tax <- unite_tax[ rownames(unite_tax)[! rownames(unite_tax) %in% c("F.langsethiae(0420)") ], ]#OTU F.oxyF.oxy <- unite_tax["F.oxysporum(07040)",]rownames(F.oxy) <- c("F.oxy")unite_tax <- rbind(unite_tax, F.oxy)unite_tax <- unite_tax[ rownames(unite_tax)[! rownames(unite_tax) %in% c("F.oxysporum(07040)") ], ]#OTU F.poaeF.poae <- unite_tax["F.poae(0378)",]rownames(F.poae) <- c("F.poae")unite_tax <- rbind(unite_tax, F.poae)unite_tax <- unite_tax[ rownames(unite_tax)[! rownames(unite_tax) %in% c("F.poae(0378)", "F.poae(0338)", "F.poae(07027)") ], ]#OTU F.prolF.prol <- unite_tax["F.proliferatum(05010)",]rownames(F.prol) <- c("F.prol")unite_tax <- rbind(unite_tax, F.prol)unite_tax <- unite_tax[ rownames(unite_tax)[! rownames(unite_tax) %in% c("F.proliferatum(05010)", "F.proliferatum(7046)") ], ]#OTU F.sporoF.sporo <-unite_tax["F.sporotrichoides(7044)",]rownames(F.sporo) <- c("F.sporo")unite_tax <- rbind(unite_tax, F.sporo)unite_tax <- unite_tax[ rownames(unite_tax)[! rownames(unite_tax) %in% c("F.sporotrichoides(7044)") ], ]#OTU F.subF.sub <- unite_tax["F.subglutinans(07038)",]rownames(F.sub) <- c("F.sub")unite_tax <- rbind(unite_tax, F.sub)unite_tax <- unite_tax[ rownames(unite_tax)[! rownames(unite_tax) %in% c("F.subglutinans(07038)", "F.subglutinans(7043)") ], ]#OTU F.venF.ven <- unite_tax["F.venenatum(11020)",]rownames(F.ven) <- c("F.ven")unite_tax <- rbind(unite_tax, F.ven)unite_tax <- unite_tax[ rownames(unite_tax)[! rownames(unite_tax) %in% c("F.venenatum(11020)") ], ]#OTU F.vertF.vert <- unite_tax["F.verticillioides(05007)",]rownames(F.vert) <- c("F.vert")unite_tax <- rbind(unite_tax, F.vert)unite_tax <- unite_tax[ rownames(unite_tax)[! rownames(unite_tax) %in% c("F.verticillioides(05007)") ], ]##Check if all Refseqs are binned to Fusarium taxasort(rownames(unite_tax))##Check ifor Plant OTUs and get rid of them in the dat filedim(dat)fungal_dat_otus <- rownames(dat) %in% rownames(subset(unite_tax, kingdom == "Fungi"))dat  <- dat[fungal_dat_otus, ]dim(dat)##Remove OTUs from taxonomy file not included in the analysisunite_tax <- unite_tax[rownames(dat), ]dim(unite_tax)rownames(unite_tax)unite_tax <- as.data.frame(unite_tax)#OTUs assigned to Fusarium/Gebberellasubset(unite_tax, genus == "Fusarium")subset(unite_tax, genus == "Gibberella")## upload RDP taxonomydatafile <- "OSCAR_RDP_tax_assignments_4R.txt"rdp_tax <- read.table( paste(datafile,sep= ""), row.names=1, sep="\t", header=F, blank.lines.skip = FALSE)dim(rdp_tax)head(rdp_tax)colnames(rdp_tax) <- c("domain", "kingdom", "subkingdom", "phylum", "subphylum",                        "class", "subclass", "order", "suborder", "family", "genus", "species")rdp_tax$kingdom <-gsub("D_1__","", rdp_tax$kingdom )rdp_tax$phylum <-gsub("D_3__","", rdp_tax$phylum )rdp_tax$class <-gsub("D_5__","", rdp_tax$class )rdp_tax$order <-gsub("D_7__","", rdp_tax$order )rdp_tax$family <-gsub("D_9__","", rdp_tax$family )rdp_tax$genus <-gsub("D_10__","", rdp_tax$genus )rdp_tax$species <-gsub("D_11__","", rdp_tax$species )head(rdp_tax)rdp_tax <- subset(rdp_tax, select=c(kingdom, phylum, class, order, family, genus, species))##Rename OTUs in Taxonomy filerownames(rdp_tax)<-gsub("denovo","otu", rownames(rdp_tax)) #change denovo to OTUhead(rdp_tax)as.matrix(rdp_tax)rownames(rdp_tax)#OTU F.ave|triF.avetri <- rdp_tax["F.avenaceum(0379)",]rownames(F.avetri) <- c("F.avetri")rdp_tax <- rbind(rdp_tax, F.avetri)rdp_tax <- rdp_tax[ rownames(rdp_tax)[! rownames(rdp_tax) %in% c("F.avenaceum(0380)", "F.avenaceum(0379)", "F.tricinctum(07015)", "F.tricinctum(05009)") ], ]#OTU F.crookF.crook <- rdp_tax["F.crookwellense(11080)",]rownames(F.crook) <- c("F.crook")rdp_tax <- rbind(rdp_tax, F.crook)rdp_tax <- rdp_tax[ rownames(rdp_tax)[! rownames(rdp_tax) %in% c("F.crookwellense(11080)","F.crookwellense(8125)", "F.crookwellense(9703)") ], ]#OTU F.culF.cul <- rdp_tax["F.culmorum(9712)",]rownames(F.cul) <- c("F.cul")rdp_tax <- rbind(rdp_tax, F.cul)rdp_tax <- rdp_tax[ rownames(rdp_tax)[! rownames(rdp_tax) %in% c("F.culmorum(9712)") ], ]#OTU F.equi_1F.equi_1 <- rdp_tax["F.equiseti(05005)",]rownames(F.equi_1) <- c("F.equi_1")rdp_tax <- rbind(rdp_tax, F.equi_1)rdp_tax <- rdp_tax[ rownames(rdp_tax)[! rownames(rdp_tax) %in% c("F.equiseti(05005)") ], ]#OTU F.equi_2F.equi_2 <- rdp_tax["F.equiseti(10015)",]rownames(F.equi_2) <- c("F.equi_2")rdp_tax <- rbind(rdp_tax, F.equi_2)rdp_tax <- rdp_tax[ rownames(rdp_tax)[! rownames(rdp_tax) %in% c("F.equiseti(10015)", "F.equiseti(11034)") ], ]#OTU F.gramF.gram <- rdp_tax["F.graminearum(0410)",]rownames(F.gram) <- c("F.gram")rdp_tax <- rbind(rdp_tax, F.gram)rdp_tax <- rdp_tax[ rownames(rdp_tax)[! rownames(rdp_tax) %in% c("F.graminearum(0410)") ], ]#OTU F.langF.lang <- rdp_tax["F.langsethiae(0420)",]rownames(F.lang) <- c("F.lang")rdp_tax <- rbind(rdp_tax, F.lang)rdp_tax <- rdp_tax[ rownames(rdp_tax)[! rownames(rdp_tax) %in% c("F.langsethiae(0420)") ], ]#OTU F.oxyF.oxy <- rdp_tax["F.oxysporum(07040)",]rownames(F.oxy) <- c("F.oxy")rdp_tax <- rbind(rdp_tax, F.oxy)rdp_tax <- rdp_tax[ rownames(rdp_tax)[! rownames(rdp_tax) %in% c("F.oxysporum(07040)") ], ]#OTU F.poaeF.poae <- rdp_tax["F.poae(0378)",]rownames(F.poae) <- c("F.poae")rdp_tax <- rbind(rdp_tax, F.poae)rdp_tax <- rdp_tax[ rownames(rdp_tax)[! rownames(rdp_tax) %in% c("F.poae(0378)", "F.poae(0338)", "F.poae(07027)") ], ]#OTU F.prolF.prol <- rdp_tax["F.proliferatum(05010)",]rownames(F.prol) <- c("F.prol")rdp_tax <- rbind(rdp_tax, F.prol)rdp_tax <- rdp_tax[ rownames(rdp_tax)[! rownames(rdp_tax) %in% c("F.proliferatum(05010)", "F.proliferatum(7046)") ], ]#OTU F.sporoF.sporo <-rdp_tax["F.sporotrichoides(7044)",]rownames(F.sporo) <- c("F.sporo")rdp_tax <- rbind(rdp_tax, F.sporo)rdp_tax <- rdp_tax[ rownames(rdp_tax)[! rownames(rdp_tax) %in% c("F.sporotrichoides(7044)") ], ]#OTU F.subF.sub <- rdp_tax["F.subglutinans(07038)",]rownames(F.sub) <- c("F.sub")rdp_tax <- rbind(rdp_tax, F.sub)rdp_tax <- rdp_tax[ rownames(rdp_tax)[! rownames(rdp_tax) %in% c("F.subglutinans(07038)", "F.subglutinans(7043)") ], ]#OTU F.venF.ven <- rdp_tax["F.venenatum(11020)",]rownames(F.ven) <- c("F.ven")rdp_tax <- rbind(rdp_tax, F.ven)rdp_tax <- rdp_tax[ rownames(rdp_tax)[! rownames(rdp_tax) %in% c("F.venenatum(11020)") ], ]#OTU F.vertF.vert <- rdp_tax["F.verticillioides(05007)",]rownames(F.vert) <- c("F.vert")rdp_tax <- rbind(rdp_tax, F.vert)rdp_tax <- rdp_tax[ rownames(rdp_tax)[! rownames(rdp_tax) %in% c("F.verticillioides(05007)") ], ]##Check if all Refseqs are binned to Fusarium taxasort(rownames(rdp_tax))##Check ifor Plant OTUs and get rid of them in the dat filedim(dat)fungal_dat_otus <- rownames(dat) %in% rownames(subset(rdp_tax, kingdom == "Fungi"))dat  <- dat[fungal_dat_otus, ]dim(dat)##Remove OTUs from taxonomy file not included in the analysisrdp_tax <- rdp_tax[rownames(dat), ]dim(rdp_tax)rownames(rdp_tax)rdp_tax <- as.data.frame(rdp_tax)#OTUs assigned to Fusarium/Gebberellasubset(rdp_tax, genus == "Fusarium")subset(rdp_tax, genus == "Gibberella")### sample depth overviewnrSeqs <-colSums(dat)pdf( paste(outputFolder,"nrSeqsSample.pdf", sep=""), width=5, height=5)barplot(nrSeqs, col="white",  cex.names=0.5, las=2)abline(h = 500, lty=3)dev.off()#### Figure 4 ####### Defining Fusarium OTUssort(rownames(dat))Fusarium_OTUs <- c("F.avetri", "F.crook",  "F.cul", "F.gram", "F.oxy", "F.poae", "F.prol", "F.sub", "F.vert" )length(Fusarium_OTUs)# normalization by sample size and transformation in "/100"dat_norm <- t(t(dat)/colSums(dat)) * 100colSums(dat_norm)dim(dat_norm)#F_ave abundance in residues, cover crops and maizeF.ave_dat_norm <- subset(dat_norm, rownames(dat_norm)=="F.avetri")sort(F.ave_dat_norm)F.ave_mean <- tapply(F.ave_dat_norm,list(design$subs, design$type),mean)F.ave_se <- tapply(F.ave_dat_norm,list(design$subs, design$type),se)sc <- c("ctrl", "radish", "vetch", "clover")cols <- c("grey60",  "gold", "darkgreen", "greenyellow")pdf(paste(RA_outputFolder, "F.avetri.pdf", sep=""))mids <- barplot(F.ave_mean,                space = c(0.2,0.6), #Returns the spaces of bars (within the group, between the group)                beside = T, legend = F, xlab = "Sample Type", ylab = "Rel. Abundance [%]", names.arg=c("Residues", "Subsidiary Crops", "Maize"),                cex.lab=1.25, ylim=c(0,50),                cex.axis=1, cex.names=1, col=cols)arrows(mids, F.ave_mean - F.ave_se, mids, F.ave_mean + F.ave_se, code = 3, angle = 90, length = 0.05)legend('topleft', legend=sc, pch=22, pt.bg=cols, bty='n', cex=1)dev.off()## Horizontal plotsF.ave_dat_norm#ResiduesF.ave_dat_norm_resid <- F.ave_dat_norm[,design$type=="1res"]F.ave_mean_resid <- tapply(F.ave_dat_norm_resid, residues_design$subs, mean)F.ave_se_resid <- tapply(F.ave_dat_norm_resid, residues_design$subs,se)pdf(paste(RA_outputFolder, "F.avetri_residues.pdf", sep=""), width=5, height=5)mids <- barplot(F.ave_mean_resid, legend = F, horiz = TRUE,                #xlab = "Rel. Abundance [%]",                names.arg=c("ctrl", "radish", "vetch", "clover"),                cex.names=1.25, xlim=c(0,37.5),                axes=FALSE,col=cols)axis(1,at=c(0,5,10),labels=c(0,5,10),cex.axis=1.25)arrows(F.ave_mean_resid - F.ave_se_resid, mids, F.ave_mean_resid + F.ave_se_resid, mids, code = 3, angle = 90, length = 0.05)#legend('topright', legend=sc, pch=22, pt.bg=cols, bty='n', cex=1)dev.off()#SubCropsF.ave_dat_norm_SubCrops <- F.ave_dat_norm[,design$type=="2sc"]F.ave_mean_SubCrops <- tapply(F.ave_dat_norm_SubCrops, SubCrops_design$subs, mean)F.ave_se_SubCrops <- tapply(F.ave_dat_norm_SubCrops, SubCrops_design$subs,se)pdf(paste(RA_outputFolder, "F.avetri_SubCrops.pdf", sep=""), width=5, height=5)mids <- barplot(F.ave_mean_SubCrops, legend = F, horiz = TRUE,                #xlab = "Rel. Abundance [%]",                cex.lab=1.25, xlim=c(0,37.5), axes=FALSE,                col=cols)axis(1,at=c(0,5,10,15,20),labels=c(0,5,10,15,20),cex.axis=1.25)arrows(F.ave_mean_SubCrops - F.ave_se_SubCrops, mids, F.ave_mean_SubCrops + F.ave_se_SubCrops, mids, code = 3, angle = 90, length = 0.05)dev.off()#maizeF.ave_dat_norm_maize <- F.ave_dat_norm[,design$type=="3maize"]F.ave_mean_maize <- tapply(F.ave_dat_norm_maize, list(maize_design$tillage, maize_design$subs), mean)F.ave_se_maize <- tapply(F.ave_dat_norm_maize, list(maize_design$tillage, maize_design$subs),se)till <- c("NT", "CT")new.plot()pdf(paste(RA_outputFolder, "F.avetri_maize.pdf", sep=""), width=5, height=5)mids <- barplot(F.ave_mean_maize, space = c(0.05,0.4),                beside = T, legend = F, horiz = TRUE,                #xlab = "Rel. Abundance [%]",                cex.lab=1.25, xlim=c(0,37.5),axes=FALSE,                density = c(25, 1000)[c(1,2,1,2,1,2,1,2)],                col=c(rep("grey",2), rep("gold",2), rep("darkgreen",2), rep("greenyellow",2)))axis(1,at=c(0,5,10,15,20,25),     labels=c(0,5,10,15,20,25),cex.axis=1.25)arrows(F.ave_mean_maize - F.ave_se_maize, mids, F.ave_mean_maize + F.ave_se_maize, mids, code = 3, angle = 90, length = 0.05)legend("bottomright", legend=till, col="black", density = c(1000, 25), bty='n', pt.cex=2)dev.off()#ANOVAs#Residuesmodel <- aov(F.ave_dat_norm_resid ~ residues_design$subs)#check for normality of the residualspar(mfrow = c(2,3))plot(model)hist(model$resid)shapiro.test(model$resid) asinTransform <- function(p) { asin(sqrt(p)) }F.ave_dat_norm_resid_Asin <- asinTransform((F.ave_dat_norm_resid/100))#Residuesmodel <- aov(F.ave_dat_norm_resid_Asin ~ residues_design$subs)#check for normality of the residualspar(mfrow = c(2,3))plot(model)hist(model$resid)shapiro.test(model$resid) #SubCropsmodel <- aov(F.ave_dat_norm_SubCrops ~ SubCrops_design$subs)#check for normality of the residualspar(mfrow = c(2,3))plot(model)hist(model$resid)shapiro.test(model$resid) #Post hocTukeyHSD(model, "SubCrops_design$subs")#Maizemodel <- aov(F.ave_dat_norm_maize ~ maize_design$subs*maize_design$tillage)#check for normality of the residualspar(mfrow = c(2,3))plot(model)hist(model$resid)shapiro.test(model$resid) asinTransform <- function(p) { asin(sqrt(p)) }F.ave_dat_norm_maize_Asin <- asinTransform((F.ave_dat_norm_maize/100))#Residuesmodel <- aov(F.ave_dat_norm_maize_Asin ~ maize_design$subs*maize_design$tillage)#check for normality of the residualspar(mfrow = c(2,3))plot(model)hist(model$resid)shapiro.test(model$resid) logitTransform <- function(p) { log(p/(1-p)) }F.ave_dat_norm_maize_log <- log2(F.ave_dat_norm_maize)#Residuesmodel <- aov(F.ave_dat_norm_maize_log ~ maize_design$subs*maize_design$tillage)#check for normality of the residualspar(mfrow = c(2,3))plot(model)hist(model$resid)shapiro.test(model$resid) non.para.model <- kruskal.test(F.ave_dat_norm_maize ~ maize_design$subs)non.para.modelnon.para.model <- kruskal.test(F.ave_dat_norm_maize ~ maize_design$tillage)non.para.modelColors <- rep("white", length(rownames(dat_norm)))names(Colors) <- rownames(dat_norm)rownames(dat_norm)Fusarium_OTUslength(Fusarium_OTUs)Fusarium_OTUs_in_dat <- Fusarium_OTUs[Fusarium_OTUs %in% rownames(dat_norm)]Colors[Fusarium_OTUs_in_dat] <- "coral"Colors#### Analysis of alpha and beta diversity in the OSCAR field trial #####otu_phyl <- otu_table(dat, taxa_are_rows=T)design_phyl <- sample_data(design)tax_phyl <- tax_table(as.matrix(unite_tax))phydat <- phyloseq(otu_phyl, design_phyl, tax_phyl)# Phyloseq Componentshead(otu_table(phydat))head(tax_table(phydat))sample_data(phydat)### Rarefyset.seed(123)dat_rar <- rarefy_even_depth(phydat, replace = TRUE, rngseed = 123)ntaxa(dat_rar)sample_sums(dat_rar)## Figure S4Apdf(paste(profiling_outputFolder,"aplpha_diversity_type.pdf", sep=""), width=5, height=5)plot_richness(dat_rar, color = "type", measures = c("Observed", "Shannon"), x = "type") +   theme_bw() + geom_boxplot() + geom_point() +   theme(axis.text.x = element_blank()) +  scale_fill_discrete(labels=c("residues", "cover crops", "maize"))dev.off()## Explore richness (alpha diversity)alpha.diversity <- estimate_richness(dat_rar, measures = c("Observed", "Chao1", "Shannon"))alpha.diversity## ANOVA on  richnessdat_rar.alpha <- cbind(sample_data(dat_rar), alpha.diversity)dat_rar.alpha.anova <- aov(Observed ~ subs*type, dat_rar.alpha)summary(dat_rar.alpha.anova) ##  highly significant type effect; no effect for subs on richnesscapture.output(summary(dat_rar.alpha.anova), file = paste(profiling_outputFolder,  "ANOVA_alphadiversiry_richness.txt", sep=""))TukeyHSD(dat_rar.alpha.anova, "type")capture.output(TukeyHSD(model), file=paste(profiling_outputFolder, "ANOVA_alphadiversiry_PostHoc.txt"))dat_rar.alpha.anova <- aov(Shannon ~ subs*type, dat_rar.alpha)summary(dat_rar.alpha.anova)  ##  highly significant type effect; no effect for subs on richnesscapture.output(summary(dat_rar.alpha.anova), file = paste(profiling_outputFolder,  "ANOVA_alphadiversiry_shannon.txt", sep=""))TukeyHSD(dat_rar.alpha.anova, "type")capture.output(TukeyHSD(model), file=paste(profiling_outputFolder, "ANOVA_shannon_PostHoc.txt"))## Figure 5A## constrained Analysis## PERMANOVAset.seed(232)dat_rar.sample <- t(get_sample(dat_rar))b.dist <- vegdist(dat_rar.sample, "bray") # help(vegdist)dataframe <- data.frame(sample_data(phydat))type <- as(sample_data(dat_rar), "data.frame")[ ,"type"]subs <- as(sample_data(dat_rar), "data.frame")[ ,"subs"]block <- as(sample_data(dat_rar), "data.frame")[ ,"Block"]## Adonis Testpaov <- adonis(b.dist ~ block + type*subs, permutations=9999)paovcapture.output(print(paov),  file= paste(profiling_outputFolder,"adonis_bray_dist.txt", sep=""))pairwise.perm.manova(b.dist, design$type, nperm=9999, p.method = "fdr")capture.output(pairwise.perm.manova(b.dist, design$type, nperm=9999, p.method = "fdr"),               file = paste(profiling_outputFolder, "pairwise.permanova.txt", sep=""))## CAP analysiscap <- ordinate(dat_rar,"CAP","bray", ~type * subs)cappermutest(cap,permutations=how(nperm=9999))plot_ordination(dat_rar,cap, shape="subs", color="type")+  geom_point(size=2.5)+  scale_shape_manual(values=c(8,15,17,19)) +  scale_fill_discrete(labels=c("Residues", "Sub. Crops", "Maize"))+  #ggtitle("constrained to type*subs+block")+  theme(legend.position="bottom",legend.title=element_blank(),legend.key = element_blank())+  guides(shape=guide_legend(nrow=2,byrow=TRUE))dat_rar.sample <- t(get_sample(dat_rar))## Indicator species corectedindicator_sp <- multipatt(as.data.frame(dat_rar.sample),design$type,func = "r.g",control=how(nperm=999))summary(indicator_sp,alpha=0.05,indvalcomp = T)indi_ssp <-indicator_sp$signindi_ssp$padj <- p.adjust(indicator_sp$sign$p.value,method="fdr")indi_spp.res <- as.matrix(indi_ssp[which(indi_ssp$index == 1 & indi_ssp$padj < 0.05),])indi_spp.res_tax <- unite_tax[rownames(indi_spp.res),]write.table(indi_spp.res_tax, file = paste(profiling_outputFolder, "indi_spp.res_tax.txt", sep=""), sep=",")indi_spp.res_rdp_tax <- rdp_tax[rownames(indi_spp.res),]write.table(indi_spp.res_rdp_tax, file = paste(profiling_outputFolder, "indi_spp.res_rdp_tax.txt", sep=""), sep=",")indi_spp.sc <- as.matrix(indi_ssp[which(indi_ssp$index == 2 & indi_ssp$padj < 0.05),])indi_spp.sc_tax <- unite_tax[rownames(indi_spp.sc),]write.table(indi_spp.sc_tax, file = paste(profiling_outputFolder, "indi_spp.sc_tax.txt", sep=""), sep=",")indi_spp.sc_rdp_tax <- rdp_tax[rownames(indi_spp.sc),]write.table(indi_spp.sc_rdp_tax, file = paste(profiling_outputFolder, "indi_spp.sc_rdp_tax.txt", sep=""), sep=",")indi_spp.maize <- as.matrix(indi_ssp[which(indi_ssp$index == 3 & indi_ssp$padj < 0.05),])indi_spp.maize_tax <- unite_tax[rownames(indi_spp.maize),]write.table(indi_spp.maize_tax, file = paste(profiling_outputFolder, "indi_spp.maize_tax.txt", sep=""), sep=",")indi_spp.maize_rdp_tax <- rdp_tax[rownames(indi_spp.maize),]write.table(indi_spp.maize_rdp_tax, file = paste(profiling_outputFolder, "indi_spp.maize_rdp_tax.txt", sep=""), sep=",")indi_spp.type <- rbind(indi_spp.res_tax, indi_spp.sc_tax)indi_spp.type <- rbind(indi_spp.type, indi_spp.maize_tax)length(rownames(indi_spp.type))### Biplot CAPsample_b.dist <- vegdist(dat_rar.sample, "bray")cap_sample <- capscale(sample_b.dist~type * subs,comm=dat_rar.sample,data=design)cap_sample_df <- data.frame(CAP1=scores(cap_sample)$sites[,1],CAP2=scores(cap_sample)$sites[,2],design)sp_cap <- envfit(cap_sample,dat_rar.sample,perm=999)sp_cap_vectors <- data.frame(scores(sp_cap,display="vectors"),pval=sp_cap$vectors$pvals)## extracting variation informationcentroids.dat <- cap_sample$CCA$centroids[, 1:2]# generate variance tablesvar_tbl.dat <- variability_table(cap_sample)var_tbl.datcap_var.dat <- cap_var_props(cap_sample)cap_var.dat# ANOVAperm_anova.dat <- anova.cca(cap_sample)print(perm_anova.dat)## defining plotting information# variation detailsCAP1 <- paste("Constrained PCoA 1 (", format(cap_var.dat[1] * 100, digits = 4), " %)", sep = "")CAP2 <- paste("Constrained PCoA 2 (", format(cap_var.dat[2] * 100, digits = 4), " %)", sep = "")title <- paste(" constrained to sample type * subsidary crop", ": [", format(var_tbl.dat["constrained", "proportion"] * 100, digits=2),                "% of variance; p = ",                format(perm_anova.dat[1, 4], digits = 2), "]",               # "; 95% CI = ", format(ci.dat[1] * 100, digits = 2),                # "%, ", format(ci.dat[2] * 100, digits = 2), "%]",               sep = "")cap_plot_sp <- ggplot(cap_sample_df)+  geom_point(aes(x=CAP1,y=CAP2,color=factor(type, labels = c("residues", "cover crops", "maize")),                 bg=factor(type, labels = c("residues", "cover crops", "maize")),                 shape=factor(subs, labels=c("control", "radish", "vetch", "clover"))),size=5, stroke=1.25)+  scale_shape_manual(values=c(21:24)) +  scale_color_manual(values=c("#a6cee3", "#b2df8a", "#1f78b4")) +  scale_fill_manual(values=c(alpha("#a6cee3",0.8), alpha( "#b2df8a", 0.8), alpha("#1f78b4", 0.8))) +  xlab(paste("CAP1 [", format(cap_var.dat[1] * 100, digits = 3), " %]", sep = ""))+  ylab(paste("CAP2 [", format(cap_var.dat[2] * 100, digits = 3), " %]", sep = ""))+  theme(axis.text= element_text(size=rel(1.25)), legend.text = element_text(size = rel(1.25)), axis.title = element_text(size = rel(1.25))) +  theme(legend.position="bottom",legend.title=element_blank(),legend.key = element_blank())+  guides(shape=guide_legend(nrow=3,byrow=TRUE)) + theme(legend.position="none")## Plot CAPplot.new()pdf(paste(profiling_outputFolder, "CAP_Bray-Curtis.pdf", sep=""), width=5, height=5)print(cap_plot_sp)dev.off()## Subset data: sample type specific## Residuesresidues <- subset_samples(phydat, type == "1res")residues <- prune_taxa(taxa_sums(residues) > 0, residues)print(residues) nsamples(residues) ntaxa(residues)sample_sums(residues)rank_names(residues)sample_variables(residues)residues_design <- subset(design, type == "1res")# Rarefyset.seed(456)residues_dat_rar <- rarefy_even_depth(residues, replace = TRUE, rngseed = 456)ntaxa(residues_dat_rar)sample_sums(residues_dat_rar)## Sub Cropssub_crops <- subset_samples(phydat, type == "2sc")sub_crops <- prune_taxa(taxa_sums(sub_crops) > 0, sub_crops)print(sub_crops) nsamples(sub_crops) ntaxa(sub_crops)sample_sums(sub_crops)rank_names(sub_crops)sample_variables(sub_crops)sub_crops_design <- subset(design, type == "2sc")# Rarefyset.seed(789)sub_crops_dat_rar <- rarefy_even_depth(sub_crops, replace = TRUE, rngseed = 789)ntaxa(sub_crops_dat_rar)sample_sums(sub_crops_dat_rar)## Maizemaize <- subset_samples(phydat, type == "3maize")print(maize) nsamples(maize) ntaxa(maize)sample_sums(maize)rank_names(maize)sample_variables(maize) maize_design <- subset(design, type == "3maize")# Rarefyset.seed(1011)maize_dat_rar <- rarefy_even_depth(maize, replace = TRUE, rngseed = 1011)ntaxa(maize_dat_rar)sample_sums(maize_dat_rar)dir.create( file.path(profiling_outputFolder, "Residues") )residues_outputFolder <- paste(profiling_outputFolder, "Residues/", sep="")sample_data(residues_dat_rar)### Explore Alpha diversity (Richness) in wheat residues## Figure S4Bpdf(paste(residues_outputFolder,"aplpha_diversity_subs.pdf", sep=""), width=5, height=5)plot_richness(residues_dat_rar, color = "subs", measures = c("Observed", "Shannon"),               x = "subs") + theme_bw() + geom_boxplot() + geom_point() + theme(axis.text.x = element_blank())dev.off()## Explore richness (alpha diversity)alpha.diversity <- estimate_richness(residues_dat_rar, measures = c("Observed", "Chao1", "Shannon"))alpha.diversity# ANOVA on  richnessdat_rar.alpha <- cbind(sample_data(residues_dat_rar), alpha.diversity)dat_rar.alpha.anova <- aov(Observed ~ subs, dat_rar.alpha)summary(dat_rar.alpha.anova) ##   significant subs effectcapture.output(summary(dat_rar.alpha.anova), file = paste(residues_outputFolder,  "ANOVA_alphadiversiry_richness.txt", sep=""))TukeyHSD(dat_rar.alpha.anova, "subs")capture.output(TukeyHSD(dat_rar.alpha.anova), file=paste(profiling_outputFolder, "ANOVA_alphadiversiry_PostHoc.txt"))dat_rar.alpha.anova <- aov(Shannon ~ subs, dat_rar.alpha)summary(dat_rar.alpha.anova)  ##  no effect for subs on richness (tendency)capture.output(summary(dat_rar.alpha.anova), file = paste(residues_outputFolder,  "ANOVA_alphadiversiry_shannon.txt", sep=""))### Explore Beta Diversity / Distance in wheat residuesresidues_dat_rar.sample <- t(get_sample(residues_dat_rar))### constrained Analysis## PERMANOVAset.seed(232)b.dist <- vegdist(residues_dat_rar.sample, "bray") # help(vegdist)dataframe <- data.frame(sample_data(residues))subs <- as(sample_data(residues_dat_rar), "data.frame")[ ,"subs"]block <- as(sample_data(residues_dat_rar), "data.frame")[ ,"Block"]## Adonis Testpaov <- adonis(b.dist ~block + subs, permutations=9999)paovcapture.output(print(paov),  file= paste(residues_outputFolder,"adonis_bray_dist.txt", sep=""))pairwise.perm.manova(b.dist, residues_design$subs, nperm=9999, p.method = "fdr")capture.output(pairwise.perm.manova(b.dist, residues_design$subs, nperm=9999, p.method = "fdr"),               file = paste(residues_outputFolder, "pairwise.permanova.txt", sep=""))## CAP analysiscap <- ordinate(residues_dat_rar,"CAP","bray", ~subs + Condition(block))cappermutest(cap,permutations=how(nperm=9999))## Indicator speciesindicator_sp <- multipatt(as.data.frame(residues_dat_rar.sample),residues_design$subs,func = "r.g",control=how(nperm=999))summary(indicator_sp,alpha=0.05,indvalcomp = T)indi_ssp <-indicator_sp$signindi_ssp$padj <- p.adjust(indicator_sp$sign$p.value,method="fdr")indi_spp.clover <- as.matrix(indi_ssp[which(indi_ssp$index == 4 & indi_ssp$padj < 0.05),])indi_spp.clover_tax <- unite_tax[rownames(indi_spp.clover),]write.table(indi_spp.clover_tax, file = paste(sub_crops_outputFolder, "indi_spp.clover_tax.txt", sep=""), sep=",")indi_spp.clover_rdp_tax <- rdp_tax[rownames(indi_spp.clover),]write.table(indi_spp.clover_rdp_tax, file = paste(sub_crops_outputFolder, "indi_spp.clover_rdp_tax.txt", sep=""), sep=",")indi_spp.vetch <- as.matrix(indi_ssp[which(indi_ssp$index == 3 & indi_ssp$padj < 0.05),])indi_spp.vetch_tax <- unite_tax[rownames(indi_spp.vetch),]write.table(indi_spp.vetch_tax, file = paste(sub_crops_outputFolder, "indi_spp.vetch_tax.txt", sep=""), sep=",")indi_spp.vetch_rdp_tax <- rdp_tax[rownames(indi_spp.vetch),]write.table(indi_spp.vetch_rdp_tax, file = paste(sub_crops_outputFolder, "indi_spp.vetch_rdp_tax.txt", sep=""), sep=",")indi_spp.radish <- as.matrix(indi_ssp[which(indi_ssp$index == 2 & indi_ssp$padj < 0.05),])indi_spp.radish_tax <- unite_tax[rownames(indi_spp.radish),]write.table(indi_spp.radish_tax, file = paste(sub_crops_outputFolder, "indi_spp.radish_tax.txt", sep=""), sep=",")indi_spp.radish_rdp_tax <- rdp_tax[rownames(indi_spp.radish),]write.table(indi_spp.radish_rdp_tax, file = paste(sub_crops_outputFolder, "indi_spp.radish_rdp_tax.txt", sep=""), sep=",")indi_spp.ctrl <- as.matrix(indi_ssp[which(indi_ssp$index == 1 & indi_ssp$padj < 0.05),])indi_spp.ctrl_tax <- unite_tax[rownames(indi_spp.ctrl),]write.table(indi_spp.ctrl_tax, file = paste(sub_crops_outputFolder, "indi_spp.ctrl_tax.txt", sep=""), sep=",")indi_spp.ctrl_rdp_tax <- rdp_tax[rownames(indi_spp.ctrl),]write.table(indi_spp.ctrl_rdp_tax, file = paste(sub_crops_outputFolder, "indi_spp.ctrl_rdp_tax.txt", sep=""), sep=",")indi_spp.residues <- rbind(indi_spp.clover, indi_spp.vetch)indi_spp.residues <- rbind(indi_spp.residues, indi_spp.radish)indi_spp.residues <- rbind(indi_spp.residues, indi_spp.ctrl)length(rownames(indi_spp.residues))### Figure 5Bsample_b.dist <- vegdist(residues_dat_rar.sample, "bray")cap_sample <- capscale(sample_b.dist~subs + Condition(block),comm=residues_dat_rar.sample,data=residues_design)cap_sample_df <- data.frame(CAP1=scores(cap_sample)$sites[,1],CAP2=scores(cap_sample)$sites[,2],residues_design)sp_cap <- envfit(cap_sample,residues_dat_rar.sample,perm=999)sp_cap_vectors <- data.frame(scores(sp_cap,display="vectors"),pval=sp_cap$vectors$pvals)## extracting variation information# get the centroids of the constrained factors (spp.)centroids.dat <- cap_sample$CCA$centroids[, 1:2]#rownames(centroids.dat) <- levels(design$type)#rownames(cap_BBN$CCA$centroids) <- levels(design_BBN$Group)# generate variance tablesvar_tbl.dat <- variability_table(cap_sample)var_tbl.datcap_var.dat <- cap_var_props(cap_sample)cap_var.dat# calculate confidence intervals for the variance of each constrained transf.ci.dat <-  cca_ci(cap_sample)ci.dat# ANOVAperm_anova.dat <- anova.cca(cap_sample)print(perm_anova.dat)## defining plotting information# variation detailsPCoA1 <- paste("Constrained PCoA 1 (", format(cap_var.dat[1] * 100, digits = 4), " %)", sep = "")PCoA2 <- paste("Constrained PCoA 2 (", format(cap_var.dat[2] * 100, digits = 4), " %)", sep = "")title <- paste(" constrained to subsidary crop", ": [", format(var_tbl.dat["constrained", "proportion"] * 100, digits=2),                "% of variance; p = ",                format(perm_anova.dat[1, 4], digits = 2), "]",               # "; 95% CI = ", format(ci.dat[1] * 100, digits = 2),                # "%, ", format(ci.dat[2] * 100, digits = 2), "%]",               sep = "")cap_plot_sp <- ggplot(cap_sample_df) +geom_point(aes(x=CAP1,y=CAP2,#color=factor(subs,labels=c("control", "radish", "vetch", "clover")),                                                     shape=factor(subs, labels=c("control", "radish", "vetch", "clover"))),color= "#a6cee3", bg= alpha("#a6cee3", 0.8), size=5, stroke = 1.25) +   #scale_color_manual(values=c(alpha("black", 1), alpha("gold2", 0.8), alpha("darkgreen", 0.8), alpha("yellowgreen", 0.8))) +  scale_shape_manual(values=c(21:24)) +  xlab(paste("CAP1 [", format(cap_var.dat[1] * 100, digits = 3), " %]", sep = ""))+  ylab(paste("CAP2 [", format(cap_var.dat[2] * 100, digits = 3), " %]", sep = ""))+  theme(legend.position="bottom",legend.title=element_blank(),legend.key = element_blank())+  guides(shape=guide_legend(nrow=1,byrow=TRUE)) +  theme(axis.text= element_text(size=rel(1.25)), legend.text = element_text(size = rel(1.25)), axis.title = element_text(size = rel(1.25))) + theme(legend.position="none")## Plot CAPplot.new()pdf(paste(residues_outputFolder, "CAP_BrayCurtis_red.pdf", sep=""), width=5, height=5)print(cap_plot_sp)dev.off()### Explore Alpha diversity (Richness) of cover crops## Figure S4Cpdf(paste(sub_crops_outputFolder,"aplpha_diversity_subs.pdf", sep=""), width=5, height=5)plot_richness(sub_crops_dat_rar, color = "subs", measures = c("Observed", "Shannon"),               x = "subs") + theme_bw() + geom_boxplot() + geom_point() + theme(axis.text.x = element_blank())dev.off()## Explore richness (alpha diversity)alpha.diversity <- estimate_richness(sub_crops_dat_rar, measures = c("Observed", "Chao1", "Shannon"))alpha.diversity# ANOVA on  richnessdat_rar.alpha <- cbind(sample_data(sub_crops_dat_rar), alpha.diversity)dat_rar.alpha.anova <- aov(Observed ~ subs + block, dat_rar.alpha)summary(dat_rar.alpha.anova) ##   significant subs effectcapture.output(summary(dat_rar.alpha.anova), file = paste(sub_crops_outputFolder,  "ANOVA_alphadiversiry_richness.txt", sep=""))TukeyHSD(dat_rar.alpha.anova, "subs")capture.output(TukeyHSD(dat_rar.alpha.anova), file=paste(profiling_outputFolder, "ANOVA_alphadiversiry_PostHoc.txt"))dat_rar.alpha.anova <- aov(Shannon ~ subs, dat_rar.alpha)summary(dat_rar.alpha.anova)  ##  no effect for subs on richness (tendency)capture.output(summary(dat_rar.alpha.anova), file = paste(sub_crops_outputFolder,  "ANOVA_richness_shannon.txt", sep=""))TukeyHSD(dat_rar.alpha.anova, "subs")capture.output(TukeyHSD(dat_rar.alpha.anova), file=paste(profiling_outputFolder, "ANOVA_shannon_PostHoc.txt"))### Explore Beta Diversity / Distance of cover cropssub_crops_dat_rar.sample <- t(get_sample(sub_crops_dat_rar))### constrained Analysis## PERMANOVAset.seed(232)b.dist <- vegdist(sub_crops_dat_rar.sample, "bray") # help(vegdist)dataframe <- data.frame(sample_data(sub_crops))subs <- as(sample_data(sub_crops_dat_rar), "data.frame")[ ,"subs"]block <- as(sample_data(sub_crops_dat_rar), "data.frame")[ ,"Block"]## Adonis Testpaov <- adonis(b.dist ~block + subs, permutations=9999)paovcapture.output(print(paov),  file= paste(sub_crops_outputFolder,"adonis_bray_dist.txt", sep=""))pairwise.perm.manova(b.dist, sub_crops_design$subs, nperm=9999, p.method = "fdr")capture.output(pairwise.perm.manova(b.dist, sub_crops_design$subs, nperm=9999, p.method = "fdr"),               file = paste(sub_crops_outputFolder, "pairwise.permanova.txt", sep=""))## CAP analysiscap <- ordinate(sub_crops_dat_rar,"CAP","bray", ~subs)cappermutest(cap,permutations=how(nperm=9999))## Indicator species corectedindicator_sp <- multipatt(as.data.frame(sub_crops_dat_rar.sample),sub_crops_design$subs,func = "r.g",control=how(nperm=999))summary(indicator_sp,alpha=0.05,indvalcomp = T)indi_ssp <-indicator_sp$signindi_ssp$padj <- p.adjust(indicator_sp$sign$p.value,method="fdr")indi_spp.clover <- as.matrix(indi_ssp[which(indi_ssp$index == 4 & indi_ssp$padj < 0.05),])indi_spp.clover_tax <- unite_tax[rownames(indi_spp.clover),]write.table(indi_spp.clover_tax, file = paste(sub_crops_outputFolder, "indi_spp.clover_tax.txt", sep=""), sep=",")indi_spp.clover_rdp_tax <- rdp_tax[rownames(indi_spp.clover),]write.table(indi_spp.clover_rdp_tax, file = paste(sub_crops_outputFolder, "indi_spp.clover_rdp_tax.txt", sep=""), sep=",")indi_spp.vetch <- as.matrix(indi_ssp[which(indi_ssp$index == 3 & indi_ssp$padj < 0.05),])indi_spp.vetch_tax <- unite_tax[rownames(indi_spp.vetch),]write.table(indi_spp.vetch_tax, file = paste(sub_crops_outputFolder, "indi_spp.vetch_tax.txt", sep=""), sep=",")indi_spp.vetch_rdp_tax <- rdp_tax[rownames(indi_spp.vetch),]write.table(indi_spp.vetch_rdp_tax, file = paste(sub_crops_outputFolder, "indi_spp.vetch_rdp_tax.txt", sep=""), sep=",")indi_spp.radish <- as.matrix(indi_ssp[which(indi_ssp$index == 2 & indi_ssp$padj < 0.05),])indi_spp.radish_tax <- unite_tax[rownames(indi_spp.radish),]write.table(indi_spp.radish_tax, file = paste(sub_crops_outputFolder, "indi_spp.radish_tax.txt", sep=""), sep=",")indi_spp.radish_rdp_tax <- rdp_tax[rownames(indi_spp.radish),]write.table(indi_spp.radish_rdp_tax, file = paste(sub_crops_outputFolder, "indi_spp.radish_rdp_tax.txt", sep=""), sep=",")indi_spp.ctrl <- as.matrix(indi_ssp[which(indi_ssp$index == 1 & indi_ssp$padj < 0.05),])indi_spp.ctrl_tax <- unite_tax[rownames(indi_spp.ctrl),]write.table(indi_spp.ctrl_tax, file = paste(sub_crops_outputFolder, "indi_spp.ctrl_tax.txt", sep=""), sep=",")indi_spp.ctrl_rdp_tax <- rdp_tax[rownames(indi_spp.ctrl),]write.table(indi_spp.ctrl_rdp_tax, file = paste(sub_crops_outputFolder, "indi_spp.ctrl_rdp_tax.txt", sep=""), sep=",")indi_spp.sub_crops <- rbind(indi_spp.clover, indi_spp.vetch)indi_spp.sub_crops <- rbind(indi_spp.radish, indi_spp.sub_crops)indi_spp.sub_crops <- rbind(indi_spp.ctrl, indi_spp.sub_crops)length(rownames(indi_spp.sub_crops))## Figure 6C# normalization by sample size and transformation in "/100"sub_crops_dat_norm <- t(sub_crops_dat_rar.sample/rowSums(sub_crops_dat_rar.sample)) * 100 colSums(sub_crops_dat_norm)dim(sub_crops_dat_norm)indi_species_counts_matrix <- sub_crops_dat_norm[rownames(indi_spp.sub_crops),]# cols <- heat.colors(10)cols <- maPalette(l="grey85",m="yellow",h="red", k=100)## Heatmap of indicator speciesbray_dist <- vegdist(sub_crops_dat_rar.sample)norm_clu <- hclust(bray_dist, "average")plot(norm_clu)norm_clu$heightclust_labs<-norm_clu$labelsclust_order<-norm_clu$orderclust_labs<-clust_labs[clust_order]treatments <- c("control", "clover", "radish", "control", "clover", "vetch", "vetch", "radish", "vetch", "radish", "clover",                "vetch", "radish", "control", "control", "clover")taxa <-  c("otu1771*","otu1380","otu299*","otu399*","otu469*","otu8","otu567*","otu2113*","otu1625","otu485","otu141",           "otu1835","otu613","otu310","otu1414*","otu1134*","otu2093","F.avetri*")plot.new()postscript(paste(sub_crops_outputFolder, "heatmap_indi_sp.eps", sep=""), width=5.5, height=5)heatmap.2(log2(indi_species_counts_matrix+1), col=cols, dendrogram="column", Rowv=F,Colv = as.dendrogram(norm_clu),          density.info="none",key.title=NA, scale="none", trace="none",  labCol=treatments,labRow=taxa,          cexRow = 1.25, cexCol = 1.25,          #sepcolor="grey95", sepwidth=c(0.0001,0.0001), colsep=1:ncol(indi_species_counts_matrix),rowsep=1:nrow(indi_species_counts_matrix),          margins=c(5,10),key.xlab=expression(paste("Relative OTU abundance"," (log"[2],")"))#,RowSideColors=otu_cols)dev.off()rownames <- paste(OTU_ID, unite_tax[OTU, "genus"])genus_unite <- paste(unite_tax[rownames(indi_spp.sub_crops), "genus"])genus_rdp <- paste(rdp_tax[rownames(indi_spp.sub_crops), "genus"])### Figure 6Asample_b.dist <- vegdist(sub_crops_dat_rar.sample, "bray")cap_sample <- capscale(sample_b.dist~subs,comm=sub_crops_dat_rar.sample,data=sub_crops_design)cap_sample_df <- data.frame(CAP1=scores(cap_sample)$sites[,1],CAP2=scores(cap_sample)$sites[,2],sub_crops_design)sp_cap <- envfit(cap_sample,sub_crops_dat_rar.sample,perm=999)sp_cap_vectors <- data.frame(scores(sp_cap,display="vectors"),pval=sp_cap$vectors$pvals)## extracting variation information# get the centroids of the constrained factors (spp.)centroids.dat <- cap_sample$CCA$centroids[, 1:2]# generate variance tablesvar_tbl.dat <- variability_table(cap_sample)var_tbl.datcap_var.dat <- cap_var_props(cap_sample)cap_var.dat# calculate confidence intervals for the variance of each constrained transf.ci.dat <-  cca_ci(cap_sample)ci.dat# ANOVAperm_anova.dat <- anova.cca(cap_sample)print(perm_anova.dat)## defining plotting information# variation detailsPCoA1 <- paste("Constrained PCoA 1 (", format(cap_var.dat[1] * 100, digits = 4), " %)", sep = "")PCoA2 <- paste("Constrained PCoA 2 (", format(cap_var.dat[2] * 100, digits = 4), " %)", sep = "")title <- paste(" constrained to subsidary crop", ": [", format(var_tbl.dat["constrained", "proportion"] * 100, digits=2),                "% of variance; p = ",                format(perm_anova.dat[1, 4], digits = 2), "]",               # "; 95% CI = ", format(ci.dat[1] * 100, digits = 2),                # "%, ", format(ci.dat[2] * 100, digits = 2), "%]",               sep = "")cap_plot_sp <- ggplot(cap_sample_df)+   geom_point(aes(x=CAP1,y=CAP2,color=factor(subs,labels=c("control", "radish", "vetch", "clover")),                 fill=factor(subs,labels=c("control", "radish", "vetch", "clover")),                 shape=factor(subs, labels=c("control", "radish", "vetch", "clover"))),             size=5) +   scale_color_manual(values=c(alpha("black", 1), alpha("gold2",1), alpha("darkgreen", 1), alpha("yellowgreen",1))) +  scale_fill_manual(values=c(alpha("black", 0.6), alpha("gold2", 0.8), alpha("darkgreen", 0.8), alpha("yellowgreen", 0.8))) +  scale_shape_manual(values=c(21:24)) +  geom_text_repel(data = sp_cap_vectors[rownames(indi_spp.clover),],                  aes(x = CAP1, y = CAP2, label = rownames(sp_cap_vectors[rownames(indi_spp.clover),])),                  color = alpha("yellowgreen",1), size = 4, show.legend =F, segment.colour = NA)+  geom_point(data = sp_cap_vectors[rownames(indi_spp.clover),],             aes(x = CAP1, y = CAP2), color= "yellowgreen", size = .75, show.legend =F)+  geom_text_repel(data = sp_cap_vectors[rownames(indi_spp.vetch),],                  aes(x = CAP1, y = CAP2, label = rownames(sp_cap_vectors[rownames(indi_spp.vetch),])),                  color = alpha("darkgreen",1), size = 4, show.legend =F, segment.colour = NA)+  geom_point(data = sp_cap_vectors[rownames(indi_spp.vetch),],             aes(x = CAP1, y = CAP2), color= "darkgreen", size = .75, show.legend =F)+  geom_text_repel(data = sp_cap_vectors[rownames(indi_spp.radish),],                  aes(x = CAP1, y = CAP2, label = rownames(sp_cap_vectors[rownames(indi_spp.radish),])),                  color = alpha("gold3",1), size = 4, show.legend =F, segment.colour = NA)+  geom_point(data = sp_cap_vectors[rownames(indi_spp.radish),],             aes(x = CAP1, y = CAP2), color= "gold3", size = .75, show.legend =F)+  geom_text_repel(data = sp_cap_vectors[rownames(indi_spp.ctrl),],                  aes(x = CAP1, y = CAP2, label = rownames(sp_cap_vectors[rownames(indi_spp.ctrl),])),                  color = alpha("grey30",1), size = 4, show.legend =F, segment.colour = NA)+  geom_point(data = sp_cap_vectors[rownames(indi_spp.ctrl),],             aes(x = CAP1, y = CAP2), color= "grey30", size = .75, show.legend =F)+  xlab(paste("CAP1 [", format(cap_var.dat[1] * 100, digits = 3), " %]", sep = ""))+  ylab(paste("CAP2 [", format(cap_var.dat[2] * 100, digits = 3), " %]", sep = ""))+  theme(legend.position="bottom",legend.title=element_blank(),legend.key = element_blank())+  guides(shape=guide_legend(nrow=1,byrow=TRUE))+  theme(axis.text= element_text(size=rel(1.25)), legend.text = element_text(size = rel(1.25)), axis.title = element_text(size = rel(1.25)))taxa <-  c("otu1771*","otu1380","otu299*","otu399*","otu469*","otu8","otu567*","otu2113*","otu1625","otu485","otu141",           "otu1835","otu613","otu310","otu1414*","otu1134*","otu2093","F.avetri*")## Plot CAPplot.new()pdf(paste(sub_crops_outputFolder, "CAP_Bray-Curtis_indi_sp.pdf", sep=""), width=5, height=5.25)print(cap_plot_sp)dev.off()### Explore Alpha diversity (Richness) in maize kernels## Figure S4Dpdf(paste(maize_outputFolder,"aplpha_diversity_subs.pdf", sep=""), width=5, height=5)plot_richness(maize_dat_rar, color = "subs", measures = c("Observed", "Shannon"),               x = "subs") + theme_bw() + geom_boxplot() + geom_point() + theme(axis.text.x = element_blank())dev.off()## Explore richness (alpha diversity)alpha.diversity <- estimate_richness(maize_dat_rar, measures = c("Observed", "Chao1", "Shannon"))alpha.diversity# ANOVA on  richnessdat_rar.alpha <- cbind(sample_data(maize_dat_rar), alpha.diversity)dat_rar.alpha.anova <- aov(Observed ~ subs*tillage, dat_rar.alpha)summary(dat_rar.alpha.anova) ##   no significant subs and till effectcapture.output(summary(dat_rar.alpha.anova), file = paste(maize_outputFolder,  "ANOVA_alphadiversiry_richness.txt", sep=""))dat_rar.alpha.anova <- aov(Shannon ~ subs*tillage, dat_rar.alpha)summary(dat_rar.alpha.anova)  ##  no significant subs and till effectcapture.output(summary(dat_rar.alpha.anova), file = paste(maize_outputFolder,  "ANOVA_alphadiversiry_shannon.txt", sep=""))### Explore Beta Diversity / Distance in maize kernelsmaize_dat_rar.sample <- t(get_sample(maize_dat_rar))### constrained Analysis## PERMANOVAset.seed(232)b.dist <- vegdist(maize_dat_rar.sample, "bray") # help(vegdist)dataframe <- data.frame(sample_data(maize))subs <- as(sample_data(maize_dat_rar), "data.frame")[ ,"subs"]tillage <- as(sample_data(maize_dat_rar), "data.frame")[ ,"tillage"]block <- as(sample_data(maize_dat_rar), "data.frame")[ ,"Block"]## Adonis Testpaov <- adonis(b.dist ~block + subs*tillage, permutations=9999)paovcapture.output(print(paov),  file= paste(maize_outputFolder,"adonis_bray_dist.txt", sep=""))## Indicator speciesindicator_sp <- multipatt(as.data.frame(maize_dat_rar.sample),maize_design$subs,func = "r.g",control=how(nperm=999))summary(indicator_sp,alpha=0.05,indvalcomp = T)indi_ssp <-indicator_sp$signindi_ssp$padj <- p.adjust(indicator_sp$sign$p.value,method="fdr")indi_spp.clover <- as.matrix(indi_ssp[which(indi_ssp$index == 4 & indi_ssp$padj < 0.05),])indi_spp.clover_tax <- unite_tax[rownames(indi_spp.clover),]write.table(indi_spp.clover_tax, file = paste(sub_crops_outputFolder, "indi_spp.clover_tax.txt", sep=""), sep=",")indi_spp.clover_rdp_tax <- rdp_tax[rownames(indi_spp.clover),]write.table(indi_spp.clover_rdp_tax, file = paste(sub_crops_outputFolder, "indi_spp.clover_rdp_tax.txt", sep=""), sep=",")indi_spp.vetch <- as.matrix(indi_ssp[which(indi_ssp$index == 3 & indi_ssp$padj < 0.05),])indi_spp.vetch_tax <- unite_tax[rownames(indi_spp.vetch),]write.table(indi_spp.vetch_tax, file = paste(sub_crops_outputFolder, "indi_spp.vetch_tax.txt", sep=""), sep=",")indi_spp.vetch_rdp_tax <- rdp_tax[rownames(indi_spp.vetch),]write.table(indi_spp.vetch_rdp_tax, file = paste(sub_crops_outputFolder, "indi_spp.vetch_rdp_tax.txt", sep=""), sep=",")indi_spp.radish <- as.matrix(indi_ssp[which(indi_ssp$index == 2 & indi_ssp$padj < 0.05),])indi_spp.radish_tax <- unite_tax[rownames(indi_spp.radish),]write.table(indi_spp.radish_tax, file = paste(sub_crops_outputFolder, "indi_spp.radish_tax.txt", sep=""), sep=",")indi_spp.radish_rdp_tax <- rdp_tax[rownames(indi_spp.radish),]write.table(indi_spp.radish_rdp_tax, file = paste(sub_crops_outputFolder, "indi_spp.radish_rdp_tax.txt", sep=""), sep=",")indi_spp.ctrl <- as.matrix(indi_ssp[which(indi_ssp$index == 1 & indi_ssp$padj < 0.05),])indi_spp.ctrl_tax <- unite_tax[rownames(indi_spp.ctrl),]write.table(indi_spp.ctrl_tax, file = paste(sub_crops_outputFolder, "indi_spp.ctrl_tax.txt", sep=""), sep=",")indi_spp.ctrl_rdp_tax <- rdp_tax[rownames(indi_spp.ctrl),]write.table(indi_spp.ctrl_rdp_tax, file = paste(sub_crops_outputFolder, "indi_spp.ctrl_rdp_tax.txt", sep=""), sep=",")indi_spp.maize <- rbind(indi_spp.clover, indi_spp.vetch)indi_spp.maize <- rbind(indi_spp.radish, indi_spp.maize)indi_spp.maize <- rbind(indi_spp.ctrl, indi_spp.maize)length(rownames(indi_spp.maize))indi_spp.ct <- as.matrix(indi_ssp[which(indi_ssp$index == 1 & indi_ssp$p.value < 0.05),])indi_spp.ct_tax <- unite_tax[rownames(indi_spp.ct),]write.table(indi_spp.ct_tax, file = paste(maize_outputFolder, "indi_spp.ct_tax.txt", sep=""), sep=",")indi_spp.nt <- as.matrix(indi_ssp[which(indi_ssp$index == 2 & indi_ssp$p.value < 0.05),])indi_spp.nt_tax <- unite_tax[rownames(indi_spp.nt),]write.table(indi_spp.nt_tax, file = paste(maize_outputFolder, "indi_spp.nt_tax.txt", sep=""), sep=",")### Figure 5Csample_b.dist <- vegdist(maize_dat_rar.sample, "bray")cap_sample <- capscale(sample_b.dist~subs*tillage,comm=maize_dat_rar.sample,data=maize_design)cap_sample_df <- data.frame(CAP1=scores(cap_sample)$sites[,1],CAP2=scores(cap_sample)$sites[,2],maize_design)sp_cap <- envfit(cap_sample,maize_dat_rar.sample,perm=999)sp_cap_vectors <- data.frame(scores(sp_cap,display="vectors"),pval=sp_cap$vectors$pvals)## extracting variation information# get the centroids of the constrained factors (spp.)centroids.dat <- cap_sample$CCA$centroids[, 1:2]# generate variance tablesvar_tbl.dat <- variability_table(cap_sample)var_tbl.datcap_var.dat <- cap_var_props(cap_sample)cap_var.dat# calculate confidence intervals for the variance of each constrained transf.ci.dat <-  cca_ci(cap_sample)ci.dat# ANOVAperm_anova.dat <- anova.cca(cap_sample)print(perm_anova.dat)## defining plotting information# variation detailsPCoA1 <- paste("Constrained PCoA 1 (", format(cap_var.dat[1] * 100, digits = 4), " %)", sep = "")PCoA2 <- paste("Constrained PCoA 2 (", format(cap_var.dat[2] * 100, digits = 4), " %)", sep = "")title <- paste(" constrained to subsidary crop*tillage", ": [", format(var_tbl.dat["constrained", "proportion"] * 100, digits=2),                "% of variance; p = ",                format(perm_anova.dat[1, 4], digits = 2), "]",               # "; 95% CI = ", format(ci.dat[1] * 100, digits = 2),                # "%, ", format(ci.dat[2] * 100, digits = 2), "%]",               sep = "")cap_plot_sp <- ggplot(cap_sample_df)+  geom_point(aes(x=CAP1,y=CAP2,fill=factor(tillage, labels=c("ct", "nt")),                 shape=factor(subs, labels=c("control", "radish", "vetch", "clover"))), color= "#1f78b4", size=5, stroke = 1.25)+  scale_shape_manual(values=c(21:24)) +  scale_fill_manual(values=c(alpha("#1f78b4",0.8), alpha("#1f78b4",0.01))) +  xlab(paste("CAP1 [", format(cap_var.dat[1] * 100, digits = 3), " %]", sep = ""))+  ylab(paste("CAP2 [", format(cap_var.dat[2] * 100, digits = 3), " %]", sep = ""))+  theme(legend.position="bottom",legend.title=element_blank(),legend.key = element_blank())+  guides(shape=guide_legend(nrow=2,byrow=TRUE))+  theme(axis.text= element_text(size=rel(1.25)), legend.text = element_text(size = rel(1.25)), axis.title = element_text(size = rel(1.25))) + theme(legend.position="none")## Plot CAPplot.new()pdf(paste(maize_outputFolder, "CAP_Bray-Curtis_blue_nl.pdf", sep=""), width=5, height=5)print(cap_plot_sp)dev.off()scale_shape_manual(values=c(21:24)) +  scale_color_manual(values=c("#F8766D", "#00BA38", "#619CFF")) +  scale_fill_manual(values=c(alpha("#F8766D",0.8), alpha( "#00BA38", 0.8), alpha("#619CFF", 0.8))) +    #### Figure S5 ######Wheat residuesresidues_dat_norm <-dat_norm[,design$type=="1res"]residues_design <-droplevels(design[design$type=="1res",])residue_mean <- apply(residues_dat_norm,1,mean)residue_se <- apply(residues_dat_norm,1,se)residue_mean <- sort(residue_mean[residue_mean > 0],decr=T)residue_meanFusarium_OTUs %in% names(residue_mean)residue_se <- residue_se[names(residue_mean)]pdf(paste(RA_outputFolder, "residue.pdf", sep=""), width=6, height=6)residue<-barplot(residue_mean[1:25], col=Colors[names(residue_mean)], ylim=c(0,50), ylab="Relative Abundance [%]",               cex.axis=1.25, cex.names=1.1, cex.lab=1.25, las=2)errorbar(residue[1:25], residue_mean[1:25], residue_se[1:25], length=0.02, lwd=1)dev.off()##Cover cropsSubCrops_dat_norm <-dat_norm[,design$type=="2sc"]SubCrops_design <-droplevels(design[design$type=="2sc",])SubCrops_mean <- apply(SubCrops_dat_norm,1,mean)SubCrops_se <- apply(SubCrops_dat_norm,1,se)SubCrops_mean <- sort(SubCrops_mean[SubCrops_mean > 0],decr=T)SubCrops_meanFusarium_OTUs %in% names(SubCrops_mean)SubCrops_se <- SubCrops_se[names(SubCrops_mean)]pdf(paste(RA_outputFolder, "SubCrops.pdf", sep=""), width=6, height=6)SubCrops<-barplot(SubCrops_mean[1:25], col=Colors[names(SubCrops_mean)], ylim=c(0,50), ylab="Relative Abundance [%]",                 cex.axis=1.25, cex.names=1.1,cex.lab=1.25, las=2)errorbar(SubCrops[1:25], SubCrops_mean[1:25], SubCrops_se[1:25], length=0.02, lwd=1)dev.off()##Maizemaize_dat_norm <-dat_norm[,design$type=="3maize"]maize_design <-droplevels(design[design$type=="3maize",])maize_mean <- apply(maize_dat_norm,1,mean)maize_se <- apply(maize_dat_norm,1,se)maize_mean <- sort(maize_mean[maize_mean > 0],decr=T)maize_meanFusarium_OTUs %in% names(maize_mean)maize_se <- maize_se[names(maize_mean)]pdf(paste(RA_outputFolder, "maize.pdf", sep=""), width=6, height=6)maize<-barplot(maize_mean[1:25], col=Colors[names(maize_mean)], ylim=c(0,50), ylab="Relative Abundance [%]",                 cex.axis=1.25, cex.names=1.1, cex.lab=1.25, las=2)errorbar(maize[1:25], maize_mean[1:25], maize_se[1:25], length=0.02, lwd=1)dev.off()
