# Supplementary material for: Community Profiling of Fusarium in Combination with Other Plant-Associated Fungi in Different Crop Species Using SMRT Sequencing
Source: Front Plant Sci. 2017 Nov 28;8:2019. doi: 10.3389/fpls.2017.02019 (PMC5712420; doi:10.3389/fpls.2017.02019)
Supplement: Supplementary file 3 [file Supplementary_Information.docx]

Supplementary Methods

*Cultivation of* Fusarium *isolates*

All *Fusarium* single conidia isolates were maintained as stock cultures in 50% glycerol at −80°C at Agroscope (Zürich, Switzerland). Fungal mycelium was produced for DNA extraction by placing individual agar plugs with mycelium and conidia from stock cultures onto potato dextrose agar (PDA; Oxoid, Hampshire, Germany) in Petri dishes (∅ 9cm). After incubation for 14 days at 19±1°C with a photoperiod of 12 h dark/12 h near-UV light, the mycelium was harvested, freeze-dried for 24h and used for DNA extraction.

*DNA extraction and PCR cycling for Sanger sequencing*

Fungal mycelium of single conidia cultures (~20 mg dry weight) was used for DNA extraction using 600 mL of Nucleo spin lysis buffer PL1 for 15 min at 65° C followed by the Nucleo spin plant II extraction kit protocol (Macherey & Nagel, Düren, Germany). Polymerase chain reactions (PCR; 20 µL) contained 1 U Phusion High Fidelity DNA Polymerase, 1x HF buffer, 200 µM dNTPs, 30nM of the primers, and 3 µL of each DNA of the different *Fusarium* isolates. All reactions were performed in an iCycler instrument (BioRad, Hercules, 135 CA, USA) with the following cycling conditions; 5 min initial denaturation at 95°C, 30 cycles of 30 s denaturation at 95°C, 30 s annealing at 55°C and 90 s elongation at 72°C, and a final elongation of 15 min. PCR amplicons were verified on a 1.5 % agarose gel (Agarose; Sigma-Aldrich, St. Louis, USA). The reactions were purified with a PCR reaction clean-up kit (Macherey-Nagel, Hilden, Germany) and sequenced using the Sanger method with *ITS1F, ITS2, LR2R_fus* and *LR6* (see Table S2 for details and references) as sequencing primers by Microsynth AG (Balgach, Switzerland).

*DNA extraction, PCR cycling and SMRT sequencing for community profiling*

Plant material (~20 mg dry weight) was used for DNA extraction using 600 mL of Nucleo spin lysis buffer PL1 for 15 min at 65° C followed by the Nucleo spin plant II extraction kit protocol (Macherey & Nagel, Düren, Germany). The DNA samples including the *Fusarium* mock communities were amplified with different primers targeting the ITS region and the 5’-prime end. The forward and reverse primers were synthesized with a 5-nucleotide-long padding sequence (see guidelines for SMRT Sequencing of PacBio) followed by an 8-nt barcode tag at the 5’ end to allow multiplexing of samples within a single sequencing run. PCR was performed using the Phusion High-Fidelity DNA polymerase system (Finnzymes, Espoo, Finland). We used 1 and 2 ng of template DNA for pure *Fusarium* samples (mock communities) and plant field samples, respectively. Reactions were prepared in a volume of 20 μ*l* and triplicate reactions per DNA sample were performed to minimize stochastic PCR effects of individual reactions. Thermal cycling was performed on a Biorad PCR Instrument (Biorad, Hamburg, Germany) with the following conditions: 5 min initial denaturation at 94°C, 30 cycles of 30 s denaturation at 94°C, 30 s annealing at 55°C and 1 min elongation at 72°C and a final elongation of 10 min. The cycle number was kept as low as possible to minimize chimera formation and to be able to interpret sequence abundance in a semi-quantitative manner (Lindahl et al. 2013). Each PCR triplicate was loaded on a 1.5% agarose gel for inspection of PCR efficiency and the lack of PCR amplicons in non-template control reactions. In case of contamination with smear, primer dimers or unspecific amplicon, the target amplicon bands (defined by a size of ~1000-2500 bp) were excised from the gel using the Gel and PCR reaction clean-up kit (Macherey-Nagel, Hilden, Germany). Replicates were sample-wise pooled and the concentration of amplicon DNA was determined for each sample using PicoGreen (Molecular Probes Inc., Eugene, Oregon, USA) measured in a fluorescence plate reader Varian Turbo GTI (Varian Inc., Poalo Alto, CA, USA). Finally, we build amplicon libraries of ~2 μg by pooling of the barcoded DNA samples. Amplicon libraries were purified with the Agencourt AMPure XP PCR Purification system (1:1.8 library/AMPure beads; Beckman Coulter, CA, USA) and thereby concentrated to a final volume of 100 μl (eluted in 10 mM Tris-HCl, pH 7.5). The hairpin sequencing adapters (SMRTbellTM templates, DNA Template Prep Kit 2.0 (250bp-<3Kb), p/n 001-540-726) were ligated to the purified amplicon libraries following the blunt-end ligation protocol of PacBio (2kb library preparation) by the Functional Genomic Centre Zurich (FGCZ; http://www.fgcz.ch Zurich, Switzerland). Sequencing libraries were purified with AMPure, assessed for quality Bioanalyzer (Agilent, G2940CA) and quantity (Qubit 1.0, q32857 Life Technologies) and were finalized for sequencing by annealing the sequencing primers to the SMRTbells and binding of the DNA polymerase to the template complex. The sequencing libraries were prepared using P6/C4 chemistry (DNA/Polymerase Binding Kit P6 (p/n 100-372-700), DNA Sequencing Reagent 4.0 (p/n 100-356-200)) on the PacBio® RS II Instrument (p/n 100-210-100). In total, 11 SMRT®Cells (V3.0 p/n 100-171-800) were sequenced depending on the number of samples in a library and/or the aimed sequencing depth (Table S4). All kits were purchased from Pacific Bioscience and used according to the manufacturers’ protocols.

*Sequence data processing*

The SMRT Portal (v1.4) was used to extract from the raw data the circular consensus sequences (CCS) of at least five passes (CCS reads present the consensus of at least five subreads) in fastq format using default parameters for consensus base calling. The CCS reads were processed in mother (v.1.35.0; (Schloss et al. 2009) and we largely followed the specific recommendations for PacBio data by Schloss *et al.* (2016). The bioinformatics script including all individual parameters and support files used are provided in the Supplementary Database 1 and 2. We converted the data from fastq to fasta and qual formats using the command fastq.info with the option pacbio=T. With the command trim.seqs we selected reads ranging from 1 to 2.2 kb in length (minlength=1000, maxlength=2200) and filtered for quality sequences consisting of reads that do not contain ambiguous base calls (maxambig=0) and a maximum of homoploymers of 8 (maxhomop=8). The CCS reads contain forward and reverse-complement oriented sequences of the amplicon insert because the blunt-end ligation of the sequencing adaptor to the PCR amplicon is not directional. Hence, we reverse complemented (using the command reverse.seqs in mother) and doubled (bash code: cat sequences.fasta sequences.rc.fasta > sequences.doubled.fasta) the quality reads for subsequent demultiplexing. Reverse complementing and doubling of the quality reads permitted to assign the reads in immediate proper orientation to their respective barcodes and samples. Quality reads were demultiplexed based on the barcode-primer sequences allowing a threshold of one mismatch (Schloss et al. 2016) using flexbar (Dodt et al. 2012). Flexbar was set to trim the barcode and primer sequences from the quality reads upon demulitplexing.

We used the same custom BASH code to name each sequence passing quality filtering and demultiplexing with a unique header containing the information to which sample it belongs as in Schlaeppi *et al.* (2016). We added the identifiers of both barcodes (e.g. ‘F2’ and ‘R3’) to the headers of the fasta sequences and additionally the library name (e.g. ‘Mthd1’) and a unique sequence number (e.g. ‘456’). Hence, each quality sequence was named with a unique header (e.g. ‘>Mthd1F2R3_456) containing the information to which sample it belongs, which permitted to pool all sequences of different libraries for common analysis (see below). The three libraries of the method evaluation and the two libraries including samples of the OSCAR field trial were process independently (Table S4, Supplementary Data 1 & 2).

We utilized QIIME 1.8 (Caporaso et al. 2010) for further downstream read processing. *De novo* chimera detection was performed on quality reads using the abundance-based algorithm implemented in UCHIME (Edgar et al. 2011). Reads were clustered into OTUs according to the open reference protocol (http://qiime.org/scripts/pick_open _reference_otus.html) using UCLUST (Edgar 2010) within QIIME applying a sequence similarity threshold of 98%. This involves first clustering reads against the hand-curated *Fusarium* reference sequence database. The reads clustered to the *Fusarium* reference dataset were subsequently binned to *Fusarium* taxa with a custom R code provided in the Supplementary Database 1 and 2 (Figure 1A). Reads that failed to assign to the reference database were clustered *de novo* into operation taxonomic units (OTUs) with UCLUST. Based on the OTU table, first sequences with a global abundance of less than 0.1% were removed, subsequently low abundant OTUs containing less than 0.5% sequences of a sample were removed and filtered from the data set yielding in a data set of just abundant community members. *Fusarium* taxa and OTUs were classified taxonomically using a QIIME-based wrapper of BLAST (Altschul et al. 1990) against the UNITE database (version 7, 01.08.2015)(Kõljalg et al. 2013) and the Ribosomal Database Project (RDP) classifier against the fungal LSU training set (version 11)(Cole et al. 2014). The OTU and taxonomy tables were filtered to exclude OTUs classified as non-fungal with the custom R code. Results of mock communities were used to define optimal settings for quality filtering, demulitplexing and cut-off for low abundant OTUs (Bokulich et al. 2012).

Fusarium *seed health test*

In SHTs, *Fusarium* species from small grain samples are determined based on species-specific morphological characteristics. Briefly, grains were surface-sterilized (10min, Chloramine T solution (1%, Riedel-de Haën, Germany)) and 100 grains were plated on PDA with 10 grains per Petri plate (∅ 9cm). The plates were incubated for 7 to 10 days at 19 ± 1°C with a photoperiod of 12 h dark/12 h near-UV light. Since it is not always possible to distinguish different *Fusarium* species on the PDA medium, all colonies resembling *Fusarium* species were transferred onto ‘Speziell nährstoffarmer Agar’ (SNA) containing a filter paper, prepared as described by Singh (1991). *Fusarium* species were identified using morphological characteristics of conidia or conidiophores according to Nelson *et al.* (1983) and Leslie and Summerell (2008). The incidence of each *Fusarium* species was expressed in percentage.

*Design of the* Fusarium *inoculation field experiment*

The experiment was carried out on the experimental farm of the research station Agroscope in Zürich, Switzerland where winter wheat (*Triticum aestivum* L. cv. ʻTitlis’) was grown on 1.3 × 5.5 m plots for each treatment. Treatments consisted of inoculations with a water-based conidial suspension of *F. poae* with Tween 20 (0.0125%) or with Tween 20 (0.0125%) alone, serving as the control treatment. Each treatment was replicated four times. The final suspensions contained 2.5 × 10^5^ conidia per milliliter and were applied at a total volume of 730 l ha^−1^ using a back-pack sprayer (width 1.5 m, 3 bar, Birchmeier M125, Birchmeier Sprühtechnik AG, Stetten, Switzerland) covering the entire plot surface. Plots were combine-harvested in July 2010. Grains were passed through a grain cleaning machine (aspiration cleaner KF12, Kongskilde, Sorø, Denmark) to remove harvest by-products. To establish subsamples with a random distribution of different grain fractions, samples were further processed using a grain divider (sample splitter RT6.5, Retsch GmbH, Haan, Germany).

*Design and sampling in the OSCAR field trial*

The investigations on diversity and dispersal of *Fusarium* fungi in different plant species along crop sequence were conducted on the OSCAR field trial during the years 2014-15 at the research station Agroscope in Tänikon, Switzerland. The field trial exhibiting a wheat-maize crop sequence was arranged in a strip-split-plot design with four replicates. On the main plots, two different tillage intensities were applied: i) conventional inversion tillage by mouldboard ploughing at about 20 cm soil depth (ct) and ii) no-tillage (nt), both applied before maize in the second year of the experiment (Figure 4A). In addition, four cover crop treatments were applied on the sub plots: i) subterranean clover (*Trifolium subterraneum* cv. Campeda) undersown in winter wheat and re-sown after wheat harvest (clover), ii) hairy vetch (*Vicia villosa* cv. Hungvillosa) as legume cover crop (vetch), iii) oilseed radish (*Raphanus sativus* cv. Pegletta) as non-legume cover crop (radish), and ii) fallow (control; Figure 4A). The second crossed subplot consisted of two N-fertilization levels (full and half dose) but only the full fertilization treatment was included in this study. In total 32 plots were considered for the current study.

Forage pea (*Pisum sativum* subsp. arvense) was grown prior to the start of the experiment, and, after ploughing the whole experimental fields, winter wheat (*Triticum aestivum* L. cv. ʻCH Claroʼ) was sown either as pure crop (vetch, radish and control cover crop treatments) or intercropped with subterranean clover (clover treatment). Weed control in wheat was performed by herbicide application (8.25 g ha-1 active ingredient (a.i.) iodosulfuron and mesosulfuron each + 180 g ha-1 a.i. fluroxypyr) in the pure wheat plots (vetch, radish and control cover crop treatments), whereas no weed control was performed in the intercropped wheat (clover treatment). The field trial was fertilized with a total of 140 kg N ha-1 in three applications (70/30/40).

After wheat harvest, all three winter cover crops (clover, vetch and radish) were sown after a shallow stubble cultivation (rotary cultivator at 5 cm soil depth). In the next spring (end of April/begin of May), cover crops were either terminated by tillage (ct) or by applying 1.44 kg ha-1 a.i. glyphosate in the nt treatment. Cover crop biomass was additionally mulched prior to tillage operations in the ct treatment.

Maize (*Zea mays* L. cv. ʻ LG 30.222ʼ) was then sowed end of May combined with an underfoot starter-fertilization of 30 kg N ha-1. Additionally, the field trial was fertilized with 60 kg N ha-1 at maize growth stage BBCH15-17. Weeds in maize were controlled by herbicides (105 g ha-1 a.i. mesotrione + 495 g ha-1 a.i. terbuthylazine + 36 g ha-1 a.i. nicosulfuron + 96 g ha-1 a.i. dicamba).

*Statistical analysis*

All analyses were performed using R (v3.3, (R Core Team 2016), and the specific R and Bioconductor packages (as indicated below). The entire statistical analysis is encoded in R script files available together with all input files required for replication of the analysis as Supplementary Database 1 and 2. Only samples with a minimum of 200 sequences were considered for analysis resulting in the removal of a single sample. Additionally, only the data of abundant community members was considered (if not otherwise mentioned). Abundance of individual taxa was normalized by the sampling depth of each sample and expressed as percentage relative abundance. For comparison of *Fusarium* communities assessed by SHT and by sequencing, *Fusarium* taxa were subtracted from the OTU table and taxa names were matched to each other. The Fusarium OTU F.ave|tri was renamed to F.ave, and the Fusarium OTUs F.equi_1 and F.equi_2 were pooled to F.equi. *Fusarium* community profiles assessed by sequencing and SHT were statistically compared based on the Spearman rank correlation.

Rarefaction analysis was performed in QIIME on the by abundance filtered OTU table (exported from R for this purpose) and on the original OTU table from 100 to 10,000 sequences with a step size of 100 and 100 iterations at each sequencing depth (Figure S1). The analysis of α- and β-diversity along the crop sequence in the OSCAR field experiment were performed using the Bioconductor package phyloseq (McMurdie and Holmes 2013). First, the OTU table was rarefied to the minimal read number among samples of a certain group (over all sample types and among residue samples 483 reads, among cover crop samples 677 reads and among maize samples 955 reads; Figure S1C). Differences in α-diversity measures (OTU richness and Shannon Index) were tested using One- or Two-way ANOVA implementing the factors *sample type*, *cover crop* and *tillage* if applicable for the group of samples (the first only for comparison over all samples, the latter only for maize samples).

To quantify the major variance factors of β-diversity along the crop rotation, we performed a multivariate analysis of fungal diversity including a testing for the experimental factors by permutational analysis of variance (PERMANOVA) followed in case of significant effects by a constrained canonical analysis of principal coordinates (CAP; (Anderson and Willis 2003)) and a characterization of the taxa responsible for the multivariate patterns based indicator species analysis (Cáceres and Legendre 2009). All p-values were adjusted for multiple testing with the FDR correction using the Benjamin-Hochberg method (Benjamini and Hochberg 1995).

Supplementary Results

*Characteristics of the sequencing method*

The three method libraries ran on 5 cells generated 162’503 raw reads of which 36 % passed quality filtering and demultiplexing (Table S4), yielding in the method dataset composed of 29 samples and 56 abundant taxa. To validate the technical reproducibility of OTU quantification, we prepared three replicate profiles of a barley grain sample by amplifying the same DNA sample in parallel reactions using three separately barcoded PCR primers. The three replicate community profiles were rarefied to a sequencing depth of 350 sequences. OTU abundances were reliably quantified as revealed in the small variation in the mean OTU abundance and pair-wise comparisons of profiles from separately barcoded replicates (Spearman rank correlation coefficient 0.72 ± 0.02 (mean ± SE, n=3); Figure S3). The two OSCAR libraries ran on 6 cells generated 272’754 raw reads of which 48 % passed quality filtering and demultiplexing (Table S4), yielding in the OSCAR dataset composed of 63 samples and 213 abundant taxa. Rarefaction analysis revealed that *c.* 500 – 1’000 sequences are needed to capture the diversity of abundant fungal taxa associated to plants a range including the sequencing depth thresholds used for rarefying in the OSCAR dataset (Figure S1).

*Fungal community composition of winter wheat residues, cover crops and maize grains*

We investigated the effect of cover crop and tillage treatments (the latter only for maize) on fungal communities separately for the different sample types. We wanted to test how the presence of different cover crops influenced the fungal communities in wheat residues collected at the end of cover cropping period. However, the fungal communities were only slightly affected by cover crop treatments in wheat residues. We found a rather low species richness in clover compared to the other cover crop treatments, but only significantly differing from vetch (Figure S4B; Table S5). Spatial variability (block effect) and not the cover crop treatments contributed most to the detected variance in β-diversity (Table S7). Although PERMANOVA revealed also cover crops to be a significant driver for differences between fungal communities, the communities could not be differentiated significantly based on pairwise comparison (Tables S7). In this context, also CAP, conditioned for the significant block effect, showed only weak importance of cover crop treatments for fungal community composition in wheat residues as only 23 % of the variation is explained by the constrained factor (Figure 5B).

Further, we investigated in more detail the phyllosphere fungal communities of the different cover crops and we found that different cover crop species harboured species-specific fungal communities (Figure 6A). However, differences in α-diversity were rather low (Figure S4C; Table S5). Nonetheless, radish exhibited the lowest richness in comparison with the other cover crop species. The differences in β-diversity between cover crops were much evident and statistically supported by PERMANOVA (Figure 6A; Table S7). Fungal communities of the two legume cover crops clover and vetch clustered close together, whereas radish and weeds occurring on the control plots exhibited distinct fungal communities.

In maize grain samples, we did not find significant support for an effect of the factors cover crop nor tillage to have an influence on the fungal communities (Figure 5C, Tables S7). Also, spatial variabilities (block effect) contributed not significantly to the detected variance in β-diversity in maize samples (Table S7).

*Indicator species along the wheat-maize sequence*

We further identified sample type- and treatment-sensitive OTUs based on indicator statistics. We found 55 fungal OTUs that were significantly associated with a specific sample type (wheat residues, cover crop shoots or maize grains), of which 25 OTUs were associated with cover crops (Table S8). Remarkably, we found only treatment-sensitive taxa in the cover crop samples. In detail, 18 fungal OTUs that were significantly associated with specific cover crop treatments (Figure 6, Table 1). Vetch and weeds sampled on the control plots harboured several OTUs linked to plant pathogens. The OTUs *F.ave/tri*, 1414 and 1134, assigned to the wheat pathogens *F. avenaceum/F. trincinctum, Zymoseptoria tritici* (formerly known as *Mycosphaerella graminicola*, synonym *Septoria tritici*) and to *Parastagonospora nodorum* (formerly known as *Septoria nodorum*) were more abundant in the vetch samples (Figure 6B). The control plots harboured also several potential plant pathogens: OTU 469, also assigned to *Z. tritici* was found to be associated to the control treatment. Other potentially phytopathological OTUs associated to the control treatments were assigned to the genus *Cryptococcus* and the species *Oculimacula yallundae* (Figure 6B, Table 1).

Supplementary Figures

**Figure S1** Sample-based rarefaction curves displaying number of observed operational taxonomic units (OTUs) against sequencing depth (number of reads). Random subsamplings were conducted for sequencing depths 100 to 10’0000 sequences in steps of 100 sequences. For each sequencing depth, 1’000 iterations were executed. Samples with a sequencing depth of more 1’000 than reads are presented. Data sets of the method development (A,B) and of the OSCAR field experiment (C,D) were treated separately. Both, rarefaction curves of abundant community members (A,C; only OTUs with a higher relative abundance than 0.5% were considered) and of the unfiltered dataset (B,D) are presented. Orange lines represent the samples of mock communities. Dashed lines indicate rarefaction depths used for diversity analyses in the OSCAR field trial (thresholds: 482, 677 and 955 reads).

**Figure S2**  Relative abundance of taxa determined by amplicon sequencing as influenced by different primer pairs. Amplicons were generated by the primer pairs *ITS1f:LR5* (1), *ITS1f:LR6* (2), *fITS7:LR5* (3) and *fITS7:LR6* (4). Three different samples types were used for the primer testing; i) maize grains heavily infested by *Fusarium graminearum*, ii) a composite sample of weeds and iii) wheat shoots. Bars represent relative (rel.) abundance of plant and fungal taxa on kingdom (A) and fungal taxa on genus (B) level.

**Figure S3**  Technical reproducibility. Three parallel reactions from the same DNA sample extracted of barley grains were produced using three separately barcoded PCR primers. Community profiles are normalized by their sequence number and expressed as percentage relative abundance. (A) Pair-wise comparisons of OTU abundances from differentially barcoded primers (circle: barcode 1 vs. 2; triangle: barcode 1 vs. 3 and asterisk: barcode 2 vs. 3) and corresponding Spearman rank correlation coefficients. (B) Ranked mean OTU abundances (±SE) of the 20 most abundant OTUs. *Fusarium* OTUs are highlighted in orange.

**Figure S4** Effects of sample type and cover crop treatment on OTU richness (Observed) and Shannon Evenness. The boxplots show the α-diversity measures for the rarefied dataset overall sample types (A; residues (1res), cover crops (2sc), maize grains (3maize)), of wheat residues (B), of cover crops (C) and of maize grains (D). Significant differences between means are indicated with letters (p < 0.05).

**Figure S5**  Fungal communities associated in different sample types in a wheat maize rotation. Ranked mean OTU abundances (±SE) of the 25 most abundant OTUs in winter wheat residues (A), cover crop shoots (B) and maize grains (C) in the OSCAR field trial. *Fusarium* OTUs are highlighted in orange.

**Table S1** *Fusarium* single conidia isolates surveyed for genetic variability among species.

| Fusarium species | Isolate ID | Ref. sequence | Host plant | Canton | Year |
| --- | --- | --- | --- | --- | --- |
| *F. avenaceum* | 0379 | * | winter wheat | VD | 2003 |
| *F. avenaceum* | 0380 | * | spring wheat | ZH | 2003 |
| *F. avenaceum* | 0648 |  | winter wheat | BE | 2006 |
| *F. crookwellense* | 11080 | * | maize | AG | 2011 |
| *F. crookwellense* | 11131 |  | maize | TI | 2011 |
| *F. crookwellense* | 8125 | * | winter wheat | BE | 2008 |
| *F. culmorum* | 11132 |  | maize | TI | 2011 |
| *F. culmorum* | 9712 | * | winter wheat | ZH | 1997 |
| *F. culmorum* | 0646 |  | winter wheat | VS | 2006 |
| *F. equiseti* | 10015 | * | winter wheat | ZH | 2010 |
| *F. equiseti* | 05005 | * | maize | BE | 2005 |
| *F. equiseti* | 11071 |  | maize | BE | 2011 |
| *F. equiseti* | 11077 |  | maize | AG | 2011 |
| *F. equiseti* | 11034 | * | maize | BE | 2011 |
| *F. graminearum* | 0410 | * | winter wheat | SH | 2004 |
| *F. graminearum* | 8121 |  | winter wheat | BE | 2008 |
| *F. graminearum* | 11076 |  | maize | AG | 2011 |
| *F. graminearum* | 11127 |  | maize | VD | 2011 |
| *F. langsethiae* | 0420 | * | winter wheat | AG | 2004 |
| *F. langsethiae* | 13014 |  | oat | JU | 2013 |
| *F. langsethiae* | 13005 |  | oat | SH | 2013 |
| *F. oxysporum* | 07040 | * | maize | AG | 2007 |
| *F. oxysporum* | 10012 |  | maize | AG | 2010 |
| *F. oxysporum* | 08011 |  | maize |  | 2008 |
| *F. poae* | 0338 | * | winter wheat | BE | 2003 |
| *F. poae* | 0378 | * | winter wheat | BE | 2003 |
| *F. poae* | 07021 |  | winter wheat | BE | 2007 |
| *F. poae* | 07027 | * | winter wheat | LU | 2007 |
| *F. poae* | 07151 |  | winter wheat | AG | 2007 |
| *F. proliferatum* | 05010 | * | maize | ZH | 2005 |
| *F. proliferatum* | 07046 | * | maize | TI | 2007 |
| *F. sporotrichioides* | 07010 |  | maize | BE | 2007 |
| *F. sporotrichioides* | 07021 |  | maize | ZH | 2007 |
| *F. sporotrichioides* | 07044 | * | maize | AG | 2007 |
| *F. subglutinans* | 07038 | * | maize | TI | 2007 |
| *F. subglutinans* | 07043 | * | maize | CH | 2007 |
| *F. subglutinans* | 07058 |  | maize | CH | 2007 |
| *F. tricinctum* | 07015 | * | maize | BE | 2007 |
| *F. tricinctum* | 05009 | * | maize | ZH | 2005 |
| *F. venenatum* | 11020 |  | maize | BE | 2005 |
| *F. venenatum* | 07047 |  | maize | AG | 2007 |
| *F. verticillioides* | 05007 | * | maize | BE | 2005 |
| *F. verticillioides* | 07022 |  | maize | ZH | 2007 |
| *F. verticillioides* | 07074 |  | maize | AG | 2007 |
| *Microdochium majus* | 0327 |  | winter wheat | ZH | 2003 |

Forty-four Swiss *Fusarium* single conidia isolates representing 14 different species (*sensu lato*) were used for a survey on genetic variability of the ribosomal DNA. Genetically unique isolates, or one isolate of each genetically identical groups were selected for reference sequence data set (indicated by a star). Host plant, Swiss district of origin (Canton) and sampling year are indicated for each isolate.

**Table S2 Primer used in this study for amplification and extraction of segments of the ribosomal operon**

| **Primer** | **Sequence (5'>3')** | **Gene/locus** | **Direction** | **Target** | **Utilisation** | **Reference** |
| --- | --- | --- | --- | --- | --- | --- |
| *ITS1F* | CTTGGTCATTTAGAGGAAGTAA | ITS | fwd | Fungi | PacBio/Sanger | Gardes & Bruns 1993 |
| *ITS5* | GGAAGTAAAAGTCGTAACAAGG | ITS | fwd | Universal | *In Silico* | White et al. 1990 |
| *fITS7* | GTGARTCATCGAATCTTTG | ITS | fwd | Fungi | PacBio | Ihrmark et al. 2012 |
| *ITS2* | GCTGCGTTCTTCATCGATGC | ITS | rev | Universal | Sanger | White et al. 1990 |
| *ITS4* | TCCTCCGCTTATTGATATGC | ITS | rev | Universal | *In Silico* | White et al. 1990 |
| *LR0R* | ACCCGCTGAACTTAAGC | LSU | fwd | Universal | *In Silico* | Hopple & Vilgalys 1994 |
| *LR2R_fus* | AAGCACTTTGAAAAGAG | LSU | fwd | Fusarium | Sanger | this study |
| *LR3* | CCGTGTTTCAAGACGGG | ITS/LSU | rev | Universal | *In Silico* | Hopple & Vilgalys 1994 |
| *LR5* | TCCTGAGGGAAACTTCG | LSU | rev | Universal | PacBio | Hopple & Vilgalys 1994 |
| *LR6* | CGCCAGTTCTGCTTACC | LSU | rev | Universal | PacBio/Sanger | Hopple & Vilgalys 1994 |

Primers were used either for amplification of PCR products for subsequent SMRT sequencing (PacBio) or Sanger sequencing or for *in silico* extraction of segments of the ribosomal operon for subsequent analysis.

Table S3 Samples used for methodical comparison of *Fusarium* community profiling

| Sample ID | Crop | Variety | Canton | Year | In the frame of |
| --- | --- | --- | --- | --- | --- |
| wht103 | winter wheat | Drifter | LU | 2007 | *Fusarium* Monitoring |
| wht140 | winter wheat | Runal | ZH | 2008 | *Fusarium* Monitoring |
| wht147 | winter wheat | Siala | SH | 2008 | *Fusarium* Monitoring |
| wht187 | winter wheat | Ludwig | SG | 2008 | *Fusarium* Monitoring |
| wht208 | winter wheat | Caphorn | JU | 2008 | *Fusarium* Monitoring |
| brl18 | barley | Caravan | ZG | 2013 | *Fusarium* Monitoring |
| brl27 | barley | Meridian | SG | 2013 | *Fusarium* Monitoring |
| brl41 | barley | Semper | AG | 2013 | *Fusarium* Monitoring |
| brl88 | barley | Semper | TG | 2013 | *Fusarium* Monitoring |
| br201 | barley | Eunova | LU | 2013 | *Fusarium* Monitoring |
| mz1 | maize | Birko | ZH | 2012 | Variety trial |
| mz11 | maize | Ricardinio | ZH | 2012 | Variety trial |
| mz18 | maize | Cassilas | ZH | 2012 | Variety trial |
| mz46 | maize | Ricardinio | VD | 2012 | Variety trial |
| mz47 | maize | Ricardinio | VD | 2012 | Variety trial |

Table S4 Sequencing library overview

| Library | Nr of cells | Raw reads | Reads after processing | | | % qual reads |
| --- | --- | --- | --- | --- | --- | --- |
|  |  |  | quality | R-primer | F-primer |  |
| Mthd1 | 1 | 19264 | 17030 | 12048 | 7108 | 36.9 |
| Mthd2 | 1 | 29003 | 15139 | 10301 | 4714 | 16.3 |
| Mthd3 | 3 | 114236 | 91394 | 68321 | 47313 | 41.4 |
|  |  | 162503 |  |  | 59135 | 36.4 |
| OSC1 | 3 | 101927 | 76736 | 60399 | 44670 | 43.8 |
| OSC2 | 3 | 170827 | 138463 | 112190 | 88117 | 51.6 |
|  |  | 272754 |  |  | 132787 | 48.7 |

Overview of the sequencing efforts for the present study. The table is split into the two independent data sets method (Mthd) and OSCAR (OSC), for each of which total number of raw and processed reads as well as the proportion of quality reads are given in the undermost row of each section.

Table S5 Effects of sample types and treatments on fungal α-diversity

|  | OTU Richness | |  | Shannon Evenness | |
| --- | --- | --- | --- | --- | --- |
| Factors | F | *p* |  | F | *p* |
| Sample type (F_2,63_) | 65.524 | 8.10E-15 |  | 42.597 | 1.32E-11 |
| Cover Crops (F_3,63_) | 2.437 | 0.0752 |  | 1.396 | 0.2547 |
| Sample type * Cover crops (F_6,63_) | 2.652 | 0.0257 |  | 2.513 | 0.0331 |
|  |  |  |  |  |  |
| Wheat residues |  |  |  |  |  |
|  | OTU Richness | |  | Shannon Evenness | |
| Factor | F | *p* |  | F | *p* |
| Cover crops (F_3,16_) | 4.717 | 0.0213 |  | 3.724 | 0.0421 |
|  |  |  |  |  |  |
| Cover crops |  |  |  |  |  |
|  | OTU Richness | |  | Shannon Evenness | |
| Factor | F | *p* |  | F | *p* |
| Cover crops (F_3,16_) | 7.214 | 0.00602 |  | 8.971 | 0.00216 |
|  |  |  |  |  |  |
| Maize grains |  |  |  |  |  |
|  | OTU Richness | |  | Shannon Evenness | |
| Factors | F | *p* |  | F | *p* |
| Cover crops (F_3,31_) | 0.664 | 0.583 |  | 0.699 | 0.562 |
| Tillage (F_1,31_) | 0.225 | 0.64 |  | 0.001 | 0.977 |
| Cover crops * Tillage (F_3,31_) | 0.4 | 0.754 |  | 0.387 | 0.763 |

Table S6 Effects of sample types and cover crop treatment on fungal β–diversity

|  | F | *R^2^* | *p* | |  |
| --- | --- | --- | --- | --- | --- |
| Block (F_1,63_) | 1.3 | 0.011 | 0.2043 | |  |
| Sample type (F_2_,_63_) | 23.46 | 0.385 | < 0.001 | |  |
| Cover crops (F_3,63_) | 2.5 | 0.062 | < 0.001 | |  |
| Type * Cover crops (F_6,63_) | 2.68 | 0.132 | < 0.001 | |  |
|  |  |  |  | |  |
| Sample types |  | *p* _adjusted_ | |  | |
| Residues vs Cover crops | | < 0.001 | |  | |
| Residues vs Maize | | < 0.001 | |  | |
| Cover crops vs Maize | | < 0.001 | |  | |

Table S7 Effects of treatments on fungal β–diversity in wheat residues, cover crops and maize

| Wheat residues |  | | | |  | | | |  | |
| --- | --- | --- | --- | --- | --- | --- | --- | --- | --- | --- |
|  | F | | | | *R^2^* | | | | *p* | |
| Block (F_1,16_) | 2.428 | | | | 0.135 | | | | < 0.001 | |
| Cover crops (F_3,16_) | 1.588 | | | | 0.262 | | | | 0.0246 | |
| Pairwise comparison | | *p* _adjusted_ | | | |  | | | |  |
| Control vs radish | 0.8 | | | | |  | |  |  |  |
| Control vs clover | 0.17 | | | | |  | |  |  |  |
| Control vs vetch | 0.65 | | | | |  | |  |  |  |
| Radish vs clover | 0.17 | | | | |  | |  |  |  |
| Radish vs vetch | 0.16 | | | | |  | |  |  |  |
| Clover vs vetch | 0.17 | | | | |  | |  |  |  |
| Cover crops |  | | | |  | | | |  | |
|  | F | | | | *R^2^* | | | | *p* | |
| Block (F_1,16_) | 1.4792 | | | | 0.02703 | | | | 0.2024 | |
| Cover crops (F_3,16_) | 14.0824 | | | | 0.77197 | | | | < 0.001 | |
| Pairwise comparison | *p* _adjusted_ | | | | |  | |  | | |
| Control vs radish | 0.031 | | |  | | |  |  |  |  |
| Control vs clover | 0.031 | | |  | | |  |  |  |  |
| Control vs vetch | 0.031 | | |  | | |  |  |  |  |
| Radish vs clover | 0.031 | | |  | | |  |  |  |  |
| Radish vs vetch | 0.031 | | |  | | |  |  |  |  |
| Clover vs vetch | 0.031 | | |  | | |  |  |  |  |
| Maize grains |  | |  | | | | |  | | |
|  | F | | | | *R^2^* | | | | *p* | |
| Block (F_1,31_) | 1.11515 | | | | 0.03737 | | | | 0.3339 | |
| Cover crops (F_3,31_) | 1.37332 | | | | 0.13807 | | | | 0.1771 | |
| Tillage (F_1,31_) | 0.27504 | | | | 0.00922 | | | | 0.9726 | |
| Cover crops * tillage (F_3,31_) | 0.77618 | | | | 0.07804 | | | | 0.6957 | |

Table S8A Taxonomic assignments of sample type sensitive taxa in wheat residues

|  | phylum | class | order | family | genus | species | OTU |
| --- | --- | --- | --- | --- | --- | --- | --- |
| Wheat residues | Ascomycota | Dothideomycetes | Pleosporales | Phaeosphaeriaceae | *Parastagonospora* | *Parastagonospora nodorum* | otu327* |
|  |  |  |  |  |  |  | otu1506* |
|  |  |  |  |  |  |  | otu1691* |
|  |  |  |  |  |  |  | otu590* |
|  |  |  |  |  |  |  | otu646* |
|  |  |  |  |  |  |  | otu1253* |
|  |  |  |  |  |  |  | otu1808* |
|  |  |  |  |  |  |  | otu2093* |
|  |  |  |  |  |  |  | otu1100* |
|  | Ascomycota | Dothideomycetes | Pleosporales | Pleosporaceae | *Pyrenophora* | *Pyrenophora seminiperda* | otu662* |
|  |  |  |  |  |  |  | otu2128* |
|  | Ascomycota | Dothideomycetes | Pleosporales | Pleosporaceae |  |  | otu507 |
|  | Ascomycota | Dothideomycetes | Pleosporales |  |  |  | otu1389 |
|  |  |  |  |  |  |  | otu1007 |
|  | Ascomycota | Eurotiomycetes | Chaetothyriales | Herpotrichiellaceae | *Exophiala* | *Exophiala opportunistica* | otu1740 |
|  | Ascomycota | Leotiomycetes | Helotiales | Incertae sedis | *Oculimacula* | *Oculimacula yallundae* | otu299 |
|  |  |  |  |  |  |  | otu399 |
|  | Ascomycota | Sordariomycetes |  |  |  |  | otu1295 |
|  |  |  |  |  |  |  | otu558 |
|  | Basidiomycota | Agaricomycetes | Corticiales | Corticiaceae | *Laetisaria* | *Laetisaria fuciformis* | otu2015 |
|  |  |  |  |  |  |  | otu780 |
|  |  |  |  |  |  |  | otu635 |
|  | Basidiomycota | Agaricostilbomycetes |  |  |  |  | otu1859 |
|  |  |  |  |  |  |  | otu574 |

Taxonomy was assigned by making use of the UNITE or RDP (indicated with *) database (for details see Material and Methods).

Table S8B Taxonomic assignments of sample type sensitive taxa in cover crops and maize grains

|  | phylum | class | order | family | genus | species | OTU |
| --- | --- | --- | --- | --- | --- | --- | --- |
| Cover crops | Ascomycota | Dothideomycetes | Capnodiales | Mycosphaerellaceae | *Zymoseptoria* | *Zymoseptoria tritici* | otu1414* |
|  | Ascomycota | Dothideomycetes | Pleosporales | Pleosporaceae |  |  | otu1384* |
|  | Ascomycota | Dothideomycetes | Pleosporales |  |  |  | otu485 |
|  | Ascomycota | Dothideomycetes | Pleosporales |  |  |  | otu8 |
|  | Ascomycota | Leotiomycetes | Erysiphales | Erysiphaceae | *Leveillula* | *Leveillula elaeagni* | otu576 |
|  | Ascomycota | Leotiomycetes | Helotiales | Hyaloscyphaceae | *Hyphodiscus* | *Hyphodiscus hymeniophilus* | otu474* |
|  |  |  |  |  |  |  | otu310* |
|  | Ascomycota | Leotiomycetes | Helotiales | Incertae sedis | *Tetracladium* | *Tetracladium maxilliforme* | otu1610 |
|  |  |  |  |  |  |  | otu1546 |
|  | Ascomycota | Sordariomycetes | Xylariales | Diatrypaceae | *Eutypa* | *Eutypa lata* | otu856* |
|  | Ascomycota | Sordariomycetes | Glomerellales |  |  |  | otu1134* |
|  | Basidiomycota | Agaricomycetes | Cantharellales | Ceratobasidiaceae | *Rhizoctonia* | *Rhizoctonia solani* | otu1603* |
|  | Basidiomycota | Microbotryomycetes | Leucosporidiales | Leucosporidiaceae | *Mastigobasidium* | *Mastigobasidium intermedium* | otu613 |
|  | Basidiomycota | Microbotryomycetes | Leucosporidiales | Leucosporidiaceae | *Leucosporidium* | *Leucosporidium golubevii* | otu1380 |
|  | Basidiomycota | Microbotryomycetes | Leucosporidiales |  |  |  | otu302 |
|  | Basidiomycota | Tremellomycetes | Cystofilobasidiales | Cystofilobasidiaceae | *Udeniomyces* | *Udeniomyces pannonicus* | otu670 |
|  | Basidiomycota | Tremellomycetes | Cystofilobasidiales | Incertae sedis | *Mrakiella* | *Mrakiella aquatica* | otu648 |
|  | Basidiomycota | Tremellomycetes | Cystofilobasidiales | Cystofilobasidiaceae | *Itersonilia* | *Itersonilia perplexans* | otu1366 |
|  | Basidiomycota | Tremellomycetes | Tremellales | Incertae sedis | *Cryptococcus* |  | otu1771 |
|  | Basidiomycota | Tremellomycetes | Tremellales | Incertae sedis | *Cryptococcus* | *Cryptococcus chernovii* | otu1163 |
|  | Basidiomycota | Tremellomycetes | Tremellales | Incertae sedis | *Cryptococcus* | *Cryptococcus victoriae* | otu567 |
|  | Basidiomycota | Tremellomycetes | Tremellales | Incertae sedis | *Filobasidiella* | *Cryptococcus neoformans* | otu2113* |
|  | Basidiomycota | Tremellomycetes | Tremellales | Cystofilobasidiaceae | *Cystofilobasidium* | *Cystofilobasidium capitatum* | otu141 |
|  | Basidiomycota | Tremellomycetes | Tremellales |  |  |  | otu1644 |
|  | Basidiomycota | Tremellomycetes |  |  |  |  | otu1021 |
|  |  |  |  |  |  |  |  |
| Maize grains | Ascomycota | Dothideomycetes | Capnodiales | Davidiellaceae | *Cladosporium* | *Cladosporium cladosporioides* | otu168* |
|  | Ascomycota | Dothideomycetes | Pleosporales | unidentified | *unidentified* | *Pleosporales sp* | otu1149 |
|  | Basidiomycota | Incertae sedis | Malasseziales | Malasseziaceae | *Malassezia* | *Malassezia restricta* | otu1328 |
|  | Zygomycota | Incertae sedis | Mucorales | Lichtheimiaceae | *Rhizomucor* | *Rhizomucor miehei* | otu1664 |
|  |  |  |  |  |  |  | otu348 |
|  | Zygomycota | Incertae sedis | Mucorales | Lichtheimiaceae | *Rhizomucor* | *Rhizomucor pusillus* | otu2021 |

Taxonomy was assigned by making use of the UNITE or RDP (indicated with *) database (for details see Material and Methods).

References:

Altschul SF, Gish W, Miller W, et al (1990) Basic local alignment search tool. J Mol Biol 215:403–410. doi: 10.1016/S0022-2836(05)80360-2

Anderson MJ, Willis TJ (2003) Canonical analysis of principal coordinates: a useful method of constrained ordination for ecology. Ecology 84:511–525.

Benjamini Y, Hochberg Y (1995) Controlling the false discovery rate: a practical and powerful approach to multiple testing. J R Stat Soc Ser B 289–300.

Bokulich NA, Subramanian S, Faith JJ, et al (2012) Quality-filtering vastly improves diversity estimates from Illumina amplicon sequencing. Nat Methods 10:57–59. doi: 10.1038/nmeth.2276

Cáceres M De, Legendre P (2009) Associations between species and groups of sites: inindices and statistical inference. Ecology 90:3566–3574. doi: 10.1890/08-1823.1

Caporaso JG, Kuczynski J, Stombaugh J, et al (2010) QIIME allows analysis of high-throughput community sequencing data. Nat Methods 7:335–6. doi: 10.1038/nmeth.f.303

Cole JR, Wang Q, Fish JA, et al (2014) Ribosomal Database Project: Data and tools for high throughput rRNA analysis. Nucleic Acids Res 42:633–642. doi: 10.1093/nar/gkt1244

Dodt M, Roehr JT, Ahmed R, Dieterich C (2012) FLEXBAR- Flexible barcode and adapter processing for next-generation sequencing platforms. Biology (Basel) 1:895–905. doi: 10.3390/biology1030895

Edgar RC (2010) Search and clustering orders of magnitude faster than BLAST. Bioinformatics 26:2460–1. doi: 10.1093/bioinformatics/btq461

Edgar RC, Haas BJ, Clemente JC, et al (2011) UCHIME improves sensitivity and speed of chimera detection. Bioinformatics 27:2194–2200. doi: 10.1093/bioinformatics/btr381

Kõljalg U, Nilsson RH, Abarenkov K, et al (2013) Towards a unified paradigm for sequence-based identification of fungi. Mol Ecol 22:5271–7. doi: 10.1111/mec.12481

Leslie JF, Summerell BA (2008) The Fusarium laboratory manual. John Wiley & Sons

Lindahl BD, Nilsson RH, Tedersoo L, et al (2013) Fungal community analysis by high-throughput sequencing of amplified markers-a user ’s guide. New Phytol 199:288–299.

McMurdie PJ, Holmes S (2013) Phyloseq: an R package for reproducible interactive analysis and graphics of microbiome census data. PLoS One. doi: 10.1371/journal.pone.0061217

Nelson PE, Toussoun TA, Marasas WFO (1983) Fusarium species: an illustrated manual for identification.

R Core Team (2016) R: A language and environment for statistical computing.

Schlaeppi K, Bender SF, Mascher F, et al (2016) High-resolution community profiling of arbuscular mycorrhizal fungi. New Phytol 212:780–791. doi: 10.1111/nph.14070

Schloss PD, Jenior ML, Koumpouras CC, et al (2016) Sequencing 16S rRNA gene fragments using the PacBio SMRT DNA sequencing system. PeerJ 4:e1869. doi: 10.7717/peerj.1869

Schloss PD, Westcott SL, Ryabin T, et al (2009) Introducing mothur: Open-source, platform-independent, community-supported software for describing and comparing microbial communities. Appl Environ Microbiol 75:7537–7541. doi: 10.1128/AEM.01541-09

Singh K (1991) An illustrated manual on identification of some seed-borne Aspergilli, Fusaria, Penicillia and their mycotoxins. Danish Government Institute of Seed Pathology for Developing Countries
